# Effect of SY009, a novel SGLT1 inhibitor, on the plasma metabolome and bile acids in patients with type 2 diabetes mellitus

**DOI:** 10.3389/fendo.2025.1487058

**Published:** 2025-01-28

**Authors:** Haoyi Yang, Yuwen Zhang, Yuxin Hong, Yuan Wei, Yuning Zhu, Lei Huang, Yuanxun Yang, Runbin Sun, Juan Li

**Affiliations:** ^1^ Department of Phase I Clinical Trials Unit, Nanjing Drum Tower Hospital Clinical College of Nanjing University of Chinese Medicine, Nanjing, China; ^2^ Department of Phase I Clinical Trials Unit, China Pharmaceutical University Nanjing Drum Tower Hospital, Nanjing, China; ^3^ Phase I Clinical Trials Unit, Nanjing Drum Tower Hospital, Affiliated Hospital of Medical School, Nanjing University, Nanjing, China

**Keywords:** SY-009, SGLT1 inhibitor, type 2 diabetes mellitus, metabolomics, bile acids

## Abstract

**Context:**

As a novel SGLT1 inhibitor, SY-009 has been preliminarily confirmed in a phase Ib clinical study for its ability to reduce postprandial blood glucose in patients with type 2 diabetes mellitus (T2DM). However, the effects of SY-009 on human plasma metabolomics are still unknown.

**Objective:**

This study aimed to explore the effects of SY-009 on plasma metabolomics in patients with T2DM and the potential metabolic regulatory mechanism involved.

**Study design:**

In the phase Ib study, a total of 50 participants with T2DM were enrolled and randomly assigned to the 0.5 mg BID, 1 mg BID, 2 mg BID, 1 mg QD, and 2 mg QD dose groups, with a 4:1 random allocation within each group to receive either the SY-009 capsule or placebo. We conducted untargeted and targeted metabolomics analyses on plasma samples from the phase Ib clinical study.

**Results:**

Untargeted metabolomics revealed that, after SY009 treatment, there were differences in metabolic pathways, including primary bile acid biosynthesis; biosynthesis of unsaturated fatty acid; steroid hormone biosynthesis; purine metabolism; phenylalanine, tyrosine and tryptophan biosynthesis. In particular, the increase in bile acid-related metabolites in the 2 mg BID group was significantly greater than that in the placebo group, and unsaturated fatty acid-related metabolites decreased in both the 2 mg BID group and the placebo group, but there was no significant difference between the two groups. After comprehensive consideration, bile acids were taken as our target for accurate quantification via targeted metabolomics. Compared with those in the placebo group, the levels of several bile acids were significantly greater in the SY-009-treated groups. Moreover, the proportion of free bile acids decreased significantly, the proportion of glycine-conjugated bile acids increased significantly, the proportion of taurine-conjugated bile acids tended to be stable, and PBA/SBA significantly increased after SY-009 administration.

**Conclusions:**

SY-009 caused a series of postprandial plasma metabolite changes in patients with T2DM, especially significant changes in the bile acid profile, which provides a new perspective on the mechanism by which SY-009 lowers blood glucose.

**Clinical trial registration:**

https://www.clinicaltrials.gov, identifier NCT04345107.

## 1 Introduction

Diabetes is a common chronic metabolic disease characterized by hyperglycaemia, with type 2 diabetes mellitus (T2DM) being the most common form, accounting for more than 90% of all diabetes cases worldwide. According to the statistical analysis of the International Diabetes Federation (IDF), the number of diabetic patients worldwide will reach 783 million in 2045 (1). The epidemic of diabetes has become a major global issue. There is an urgent need to explore new targets and optimize glycaemic control strategies.

Recently, the sodium-glucose cotransporter (SGLT) has emerged as a hot new target because of its unique hypoglycaemic mechanism. There are six main subtypes of SGLTs: SGLT1, SGLT2, SGLT3, SGLT4, SGLT5, and SGLT6. However, only the SGLT1 and SGLT2 subtypes have been extensively studied (2). SGLT1 is primarily responsible for absorbing glucose and galactose in the intestines, whereas SGLT2 is responsible for reabsorbing glucose in the kidneys (3). Both operate through active transport mechanisms that depend on sodium cooperative transport systems (4). In the first stage, glucose or galactose enters the cell from the lumen through SGLTs and accumulates. In the second stage, facilitated diffusion occurs from the cell into the blood via glucose transporter 2 (GLUT2), which islocated in the basolateral membrane.

In recent years, SGLT2 inhibitors and SGLT1/2 dual-target inhibitors have been marketed and approved for treating T2DM (5, 6). Currently, SGLT2 inhibitors have become a new mainstay in the treatment of T2DM (7), especially in combination with cardiovascular and renal disease, where SGLT2 inhibitors offer important benefits (8, 9). Compared with SGLT2 inhibitors alone, SGLT1/2 dual-target inhibitors have a greater effect on lowering blood glucose (10). SGLT1 inhibitors have not yet been marketed. However, research on SGLT1 inhibitors continues actively. For example, a novel nonabsorbable SGLT1 inhibitor, LX2761, which was developed by modifying Sotagliflozin, has demonstrated the ability to delay intestinal glucose absorption, thereby improving glycaemic control (11). In addition, SGLT1 inhibition has been shown to delay postprandial intestinal glucose absorption and increase the plasma levels of GLP-1 and GIP in healthy volunteers (12). In addition, for diabetic patients with renal insufficiency, SGLT1 inhibitors that act on the intestine may be better treatment options.

Since it was first proposed by J.K. Nicholson et al. in 1999 (13), metabolomics has become a powerful tool for screening disease biomarkers and studying the mechanisms of disease occurrence and development. Xing Chen et al. revealed the metabonomics-based renoprotective mechanism of empagliflozin in obese mice for the first time (14), which was the first application of metabolomics in SGLT inhibitors. In addition, many studies on T2DM have been based on metabolomics technology (15). Xiaoyi Yu et al., on the basis of GC-MS, reported that metabolic disorders of the tricarboxylic acid cycle (TCA) may have an important relationship with diabetic nephropathy (16). Chang Young Ha et al. reported that decanoyl carnitine and lysoPC (C14:0) are the best metabolites for predicting the risk of developing T2DM via UPLC-TOF-MS (17). Additionally, a series of potential biomarkers have been identified, including bile acid, 3-hydroxybutyric acid with ketogenesis, 2-hydroxybutyric acid, branched-chain amino acids, and aromatic amino acids (18–21). In summary, diabetes metabolomics provides researchers with a new perspective to uncover global changes in T2DM and better understand its pathophysiological mechanisms.

SY-009 is a novel SGLT1 inhibitor, that is not listed at home or abroad. With the permission of Eli Lilly Company, Suzhou Yabao Pharmaceutical Research and Development Co., Ltd., initiated the clinical study as the sponsor. Currently, a phase Ib clinical study has been completed, indicating that SY-009 can significantly reduce postprandial blood glucose (22). However, research on SGLT1 inhibitors in human plasma metabolomics is currently lacking. Therefore, this study aimed to use metabolomics technology to clarify the effects of SY-009 on the plasma metabolome profile of patients with T2DM, search for potential biomarkers, and explore the potential metabolic regulatory mechanisms of SY-009 in T2DM.

## 2 Materials and methods

### 2.1 Participants and study design

In this study, plasma samples were collected from “A randomized, double-blind, placebo-controlled, dose-escalation Phase Ib study to evaluate the safety, tolerability, and PK/PD profile of SY-009 in patients with T2DM” conducted at the Nanjing Drum Tower Hospital Phase I Clinical Trial Center.

In the phase Ib study, 50 participants with T2DM were randomly assigned to the 1 mg, 2 mg, or 4 mg daily dose groups (**Supplementary Figure S1**). The 1 mg daily dose group was divided into two administration groups, 0.5 mg BID and 1 mg QD; the 2 mg daily dose group was divided into two administration groups, 1 mg BID and 2 mg QD; and the 4 mg daily dose group was administered 2 mg BID. Ten participants were enrolled in each of these dosing groups and randomly assigned to receive SY-009 capsules or placebo at a ratio of 4:1 to be taken orally immediately before meals (QD before breakfast, BID before breakfast and dinner). Subjects were first dosed before breakfast on Day 1, followed by continuous daily dosing until dinner on Day 7.

Only blood samples from Day 1 and Day 7 were collected (**Supplementary Figure S1**). The blood collection points were 10 min before administration and 10 min, 0.5 h, 1 h, 2 h, and 4 h after administration. After blood collection, the collection tubes were placed in a precooled low-temperature centrifuge and centrifuged at 4°C and 1500 × g for 10 min to separate the plasma. Before metabolomics analysis, the plasma samples were stored at -80°C.

### 2.2 Untargeted metabolomics

#### 2.2.1 Sample preparation

The sample preparation method was as follows: 50 μL of plasma was taken into a centrifuge tube, followed by the addition of 150 μL of methanol solution containing the internal standard (4-chlorophenylalanine, 1 μg/mL). The samples were shaken for 3 min and then centrifuged at 18000 rpm for 5 min at 4°C. Next, 150 μL of the supernatant was transferred to an EP tube. The mixture was subsequently centrifuge again in the same way, after which 100 μL of the supernatant was transferred into a sample vial. Subsequently, 30 μl of each sample was mixed to prepare the quality control (QC) samples. QC samples are essential for system regulation and quality control processes, which help to obtain reliable and high-quality metabolomics data.

#### 2.2.2 LC-QTOF/MS instrument conditions

Liquid chromatography coupled with a quadrupole time-of-flight tandem mass spectrometer (AB SCIEX TripleTOF^®^5600 LC-QTOF/MS, Foster City, Canada) was used to determine the changes in the plasma metabolome before and after drug administration. The instrument conditions were as follows: the chromatographic column used was a Waters HSS T3 column (1.8 μm, 2.1×100 mm). The flow rate was 0.3 mL/min, and the column temperature was 40°C. The mobile phase was A: aqueous phase (0.1% formic acid-water) and B: organic phase (acetonitrile). The organic phase gradient elution procedure was as follows: 0-1.5 min, 5% B; 1.5-6 min, 5%-60% B; 6-9.5 min, 60%-95% B; 9.5-12 min, 95% B; 12-12.5 min, 95%-5% B; 12.5-15.5 min, 5% B. In the mass spectrometry detection, Turbo V electrospray ionization (ESI) was used for scan analysis. The parameters were set as follows: ion source gas 1 (GS1), 60 psi; ion source gas 2 (GS2), 60 psi; curtain gas (CUR), 35 psi; temperature (TEM), 550 °C; ionSpray voltage floating (ISVF), 5500 V in positive ion mode and -4500 V in negative ion mode; declustering potential (DP), 60 V in positive ion mode and -60 V in negative ion mode; collision energy (CE), 35 eV in positive ion mode and -10 eV in negative ion mode; and mass spectrum scanning range, 50-1200 Da.

#### 2.2.3 Data processing

On the basis of the untargeted raw metabolomics data (wiff format) obtained via LC-QTOF-MS, we used MSConvet and R (version 4.2.3) to convert and process the data, respectively. The steps were as follows: First, the primary mass spectrum (MS1) information of the raw data was converted to MZxml format by MSConvert, and the secondary mass spectrum (MS2) information was converted to mgf format. Then, using TidyMass package developed by Shen Xiaotao’s team (23), a series of operations, such as peak area extraction and alignment, noise signal removal, missing value filling, and metabolite identification, were carried out. The missing values were filled by the KNN method, and metabolites were identified on the basis of the KEGG and HMDB databases. Finally, the cleaned peak area data were calibrated with the MetNormalizer package for further analysis.

### 2.3 Targeted metabolomics

#### 2.3.1 Sample preparation

First, 16 bile acids, including cholic acid (CA), glycocholic acid (GCA), taurocholic acid (TCA), chenodesoxycholic acid (CDCA), glycochenodeoxycholic acid (GCDCA), taurochenodeoxycholic acid (TCDCA), deoxycholic acid (DCA), glycodesoxycholic acid (GDCA), taurodeoxycholic acid (TDCA), ursodeoxycholic acid (UDCA), glycoursodeoxycholic acid (GUDCA), tauroursodeoxycholic acid (TUDCA), hyodeoxycholic acid (HDCA), glycohyodeoxycholic acid (GHDCA), taurohyodeoxycholic acid (THDCA) and lithocholic acid (LCA) were processed for the drawing of standard curves. The mass−charge ratio (m/z) was determined according to previous methods (24).

The bile acid standard mixture and plasma samples were processed similarly. Fifty-microlitre samples were added to 200 μL of methanol solution containing an internal standard (0.1 μg/mL 2,2,4,4-D_4_-cholic acid, taurocholic-2,2,4,4-D_4_ acid, and glycocholic-2,2,4,4-D_4_ acid). Then, the mixture was shaken for 3 min and centrifuged at 18000 rpm for 5 min at 4°C. Next, 150 μL of the supernatant was transferred into an EP tube. The mixture was subsequently centrifuged again in the same way, and 100 μL of the supernatant was transferred to a sample vial. Finally, 5 μl of the sample was injected for targeted detection.

#### 2.3.2 LC-MS/MS instrument conditions

An ultra-performance liquid chromatography system with a 5500 mass spectrometer (AB Sciex, Toronto, Canada) was used to determine the changes in bile acids before and after drug administration. A Waters Atlantis T3 column (2.1×100 mm, 3 μm) was used for chromatographic separation. The mobile phase consisted of aqueous phase A and organic phase B. A: 0.1% formic acid: water, and B: methanol with the following gradient: 0-2 min 60% B, 2-10 min 60%-90% B, 10-15 min 90% B, 15-15.1 min 90%-60% B, 15.1-20 min 60% B.

#### 2.3.3 Data processing

After the plasma samples were analysed by LC-MS/MS, Analyst software**^®^** 1.7.1 was used to identify peaks corresponding to bile acids in the samples. The bile acid concentrations were subsequently obtained on the basis of standard curves and the concentrations of internal standard compounds.

### 2.4 Statistical analysis

For untargeted metabolomics analysis, principal component analysis (PCA) was used to detect the stability of the QC samples to determine whether the metabolic data were reliable. Partial least squares discrimination analysis (PLS-DA), which provides better separation than PCA does, was used to understand the difference in the total metabolites before and after administration. In addition, we combined the fold change (FC) values of metabolites before and after administration with the variable importance in projection (VIP) values obtained from PLS-DA for the preliminary screening of differentially abundant metabolites. The default criteria were as follows: VIP > 1, FC ≥ 1.5 or FC ≤ 0.67, and P < 0.05. Hierarchical cluster analysis (HCA) was performed to evaluate the similarities and differences among the potential biomarkers. Metabolic pathway enrichment analysis was performed via the KEGG database.

A paired t test was used to compare differences before and after drug administration. Multiple comparisons were performed via one-way analysis of variance (ANOVA) to analyse the differences between the SY-009 and placebo groups. Statistical analysis and data visualization were performed via MetaboAnalyst 6.0 (https://www.metaboanalyst.ca/), MetWare Cloud (https://cloud.metware.cn), Genes Cloud (https://www.genescloud.cn), Omic Studio (https://www.omicstudio.cn/tool), GraphPad Prism 9, and SPSS 27. All the data are presented as the means ± standard deviations, and P < 0.05 was considered statistically significant.

### 2.5 Study approval

This study was approved by the Ethics Committee of Nanjing Drum Tower Hospital with the ethics number 2022-187-02. The Phase Ib clinical study was registered on the website of ClinicalTrials.gov (https://www.clinicaltrials.gov/) with the identifier: NCT04345107. This study strictly complied with the Declaration of Helsinki and relevant policies and regulations, and all participants provided written informed consent before undergoing the study procedure. The criteria for inclusion and exclusion, as well as the demographic data of the participants, are presented in the **Supplementary Materials**, and the demographic and baseline characteristics were generally balanced among the groups.

## 3 Results

### 3.1 SY-009 induced changes in plasma metabolites in patients with T2DM, as determined via untargeted metabolomics

#### 3.1.1 Pattern recognition analysis revealed significant changes in the plasma metabolites of patients with T2DM after SY-009 administration

**Figure 1:**
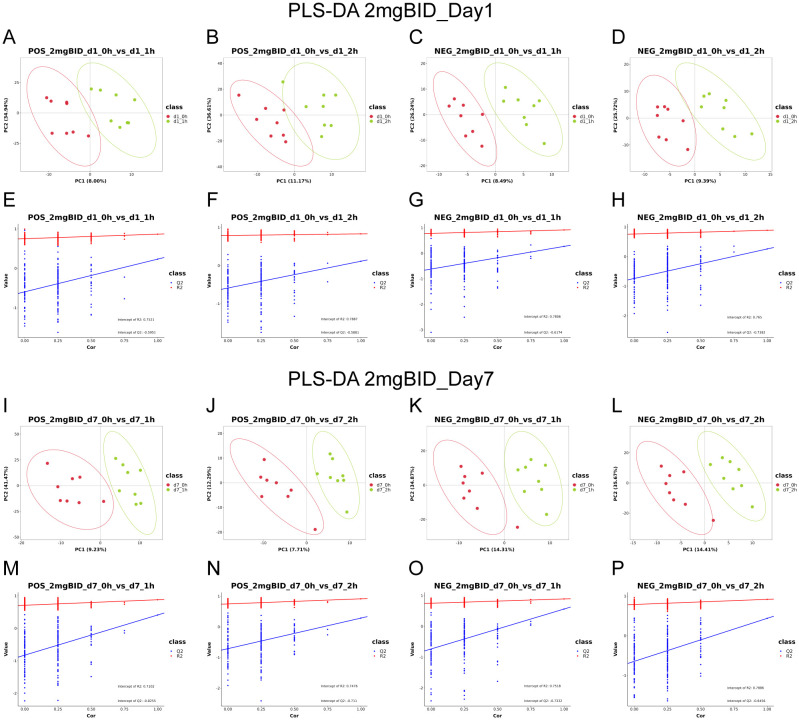
**(A–H)** are PLS-DA in positive or negative ion mode on day 1. **(I–P)** are PLS-DA in positive or negative ion mode on day 7.

For the untargeted metabolomics analysis, the representative total ion chromatograms (TICs) are shown in **Supplementary Figure S2**. After R Studio processing, firstly, PCA diagrams were first generated using the metabolic data of the QC samples and some of the plasma samples to assess data reliability. The tight clustering observed among the QC samples confirmed the accuracy and stability of the instrument (**Supplementary Figure S3**). Given that the hypoglycaemic effect of SY-009 is dose-dependent, with 2 mg BID showing the most pronounced effect (22), we initially focused on the highest-dose group, the 2 mg BID group. As shown in **Figure 1**, the PLS-DA results indicated a significant separation trend on Days 1 and 7 at 1h or 2h postadministration of SY-009 compared with preadministration levels, and there was no “overfitting” phenomenon. The model parameters are shown in **Supplementary Table S1**. These results suggest that SY-009 had a significant effect on the plasma metabolism profile of patients with T2DM.

#### 3.1.2 A series of differentially abundant metabolites were observed after SY-009 administration

Next, volcano plots of the 2 mg BID group were constructed to observe the changes in the metabolites. As shown in **Supplementary Figure S4**, on Days 1 and 7, changes in the contents of many metabolites occurred 1 h or 2 h after administration. After identification via the KEGG and HMDB databases, screening of differentially abundant metabolites was conducted basis of the following conditions: VIP>1, FC≥1.5 or FC ≤ 0.67, and P<0.05. As shown in **Supplementary Table S2**, **Supplementary Table S3**, the contents of 64 and 57 metabolites changed on Days 1 and 7, respectively. Among them, 25 and 36 metabolites with P < 0.05 were considered potential biomarkers.

#### 3.1.3 Metabolic pathways were significantly altered after SY-009 administration

**Figure 2:**
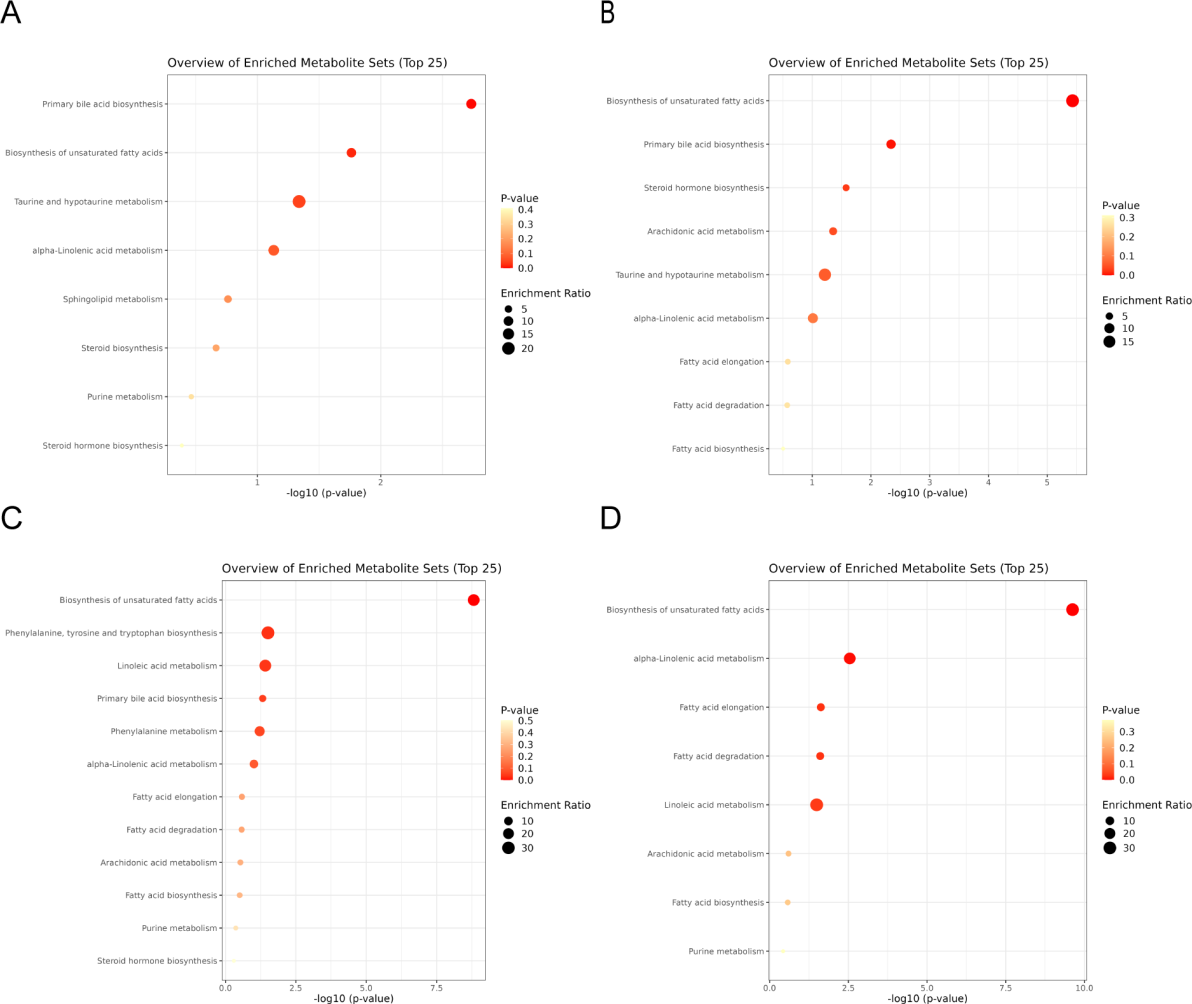
**(A, B)** are the differential metabolite pathway enrichment maps of the 2 mg BID group on day 1 and day 7, respectively. **(C, D)** are the differential metabolite pathway enrichment maps of the placebo group on day 1 and day 7, respectively.

MetaboAnalyst was utilized for metabolic pathway enrichment analysis of potential biomarkers on Days 1 and 7. As shown in **Figures 2A, B**, the biosynthesis of unsaturated fatty acids and primary bile acid biosynthesis were the two most important pathways on both Day 1 and Day 7. Therefore, these two metabolic pathways were the focus of subsequent analyses.

#### 3.1.4 Compared with the placebo, SY-009 significantly changed the metabolic profile of patients with T2DM

**Figure 3:**
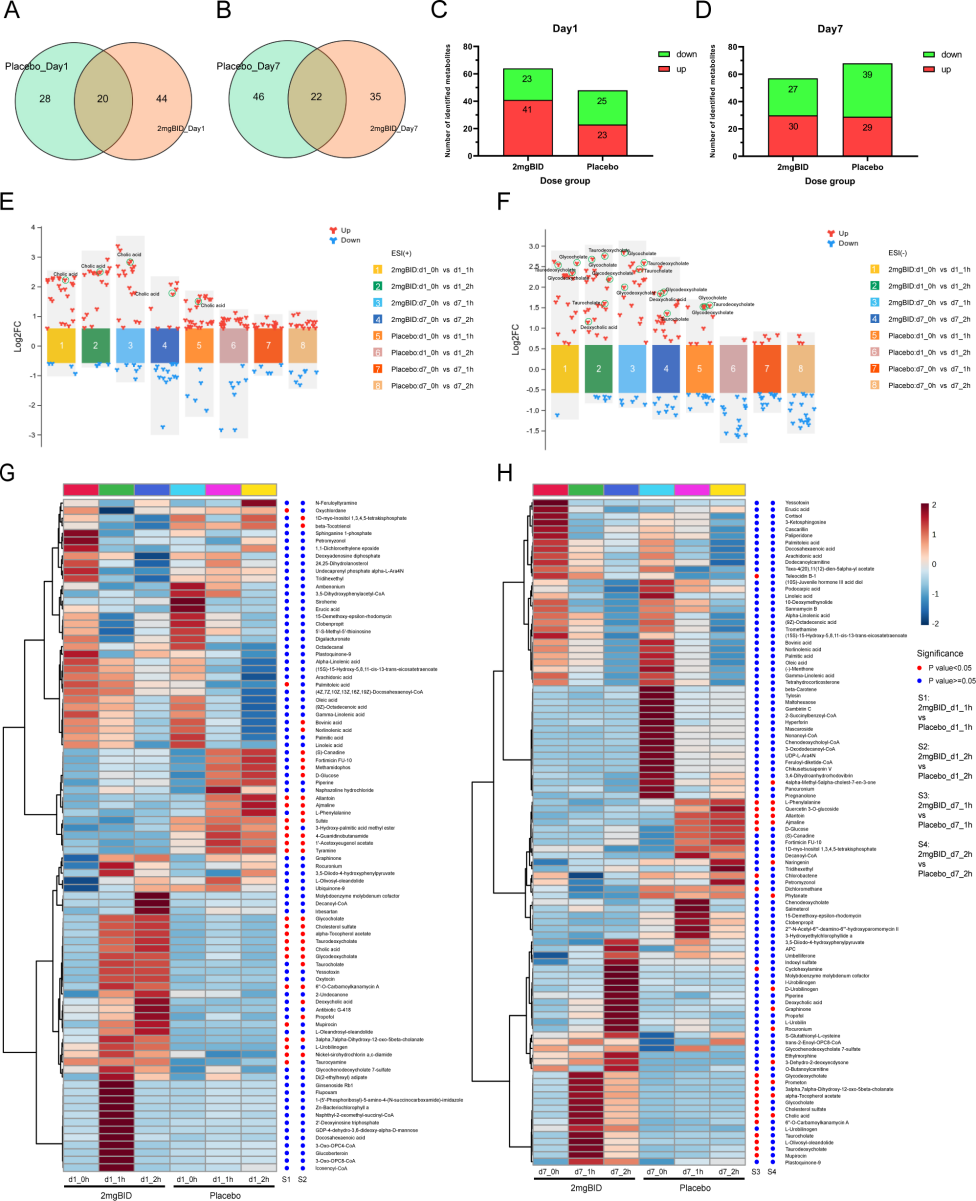
**(A, B)** Venn diagram of 2 mg BID group and placebo group on day 1 and day 7. **(C, D)** Histogram of the number of upregulated or down-regulated metabolites in the 2 mg BID group and placebo group on day 1 and day 7. **(E, F)** Multicomponent difference scatter plot of SY-009 before and after administration in positive and negative ion modes. **(G, H)** Heat maps of the 2 mg BID group and the placebo group, with dots on the right of the heat maps indicating whether the difference between the two groups was statistically significant. A red dot means P<0.05, and a blue dot means P≥0.05.

In the placebo group, differentially abundant metabolite screening and identification (**Supplementary Tables S4**, **S5**) and metabolic pathway enrichment analysis (**Figures 2C, D**) were also performed. **Figures 3A–D** show the Venn diagrams for the placebo group and the 2 mg BID group, as well as bar charts depicting the number of upregulated and downregulated metabolites. The results of the metabolic pathway enrichment analysis (**Figures 2C, D**) revealed that the biosynthesis of unsaturated fatty acids and primary bile acid biosynthesis were also enriched in the placebo group.

**Figure 4:**
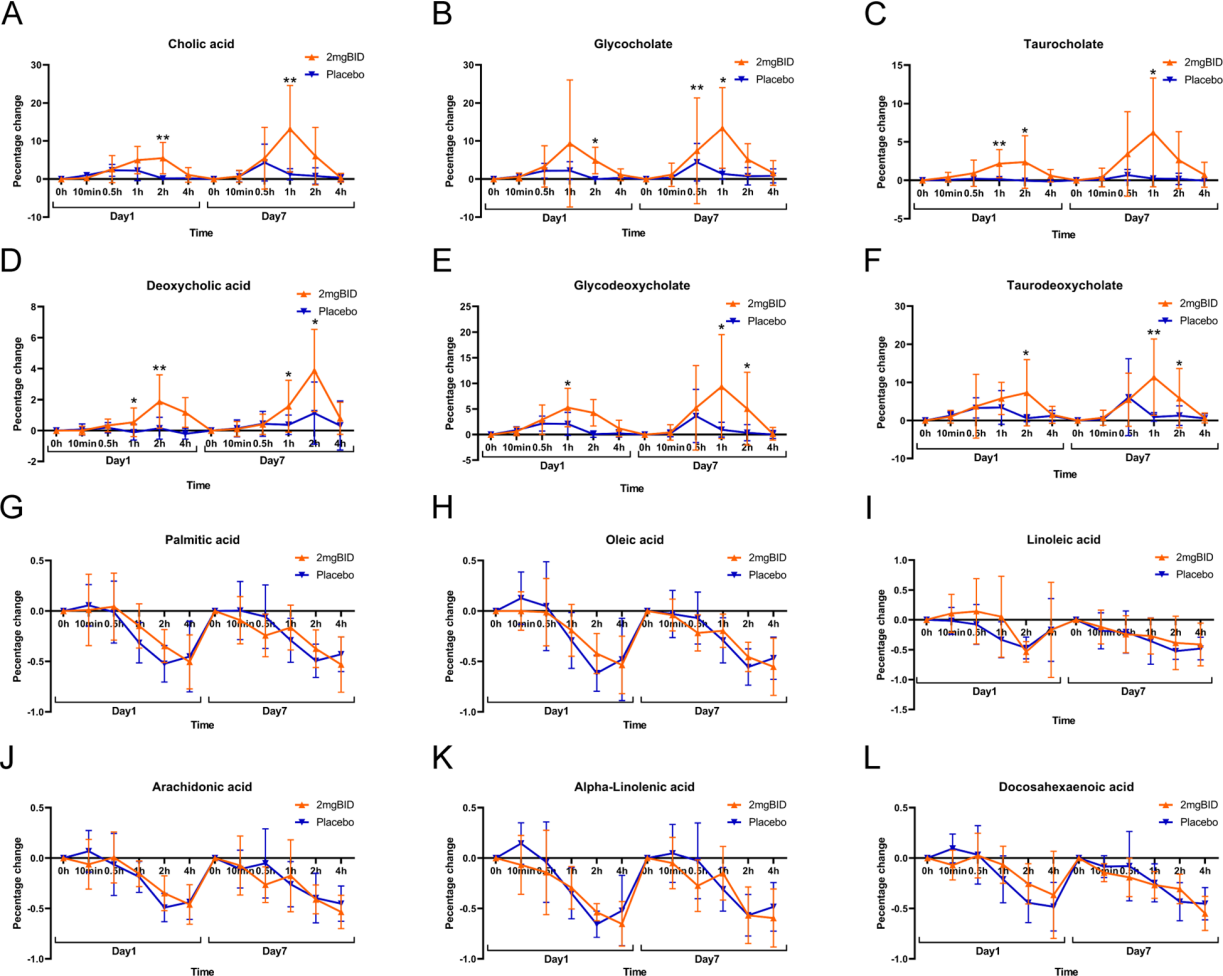
**(A–F)** Primary bile acid biosynthesis-related metabolite changes. **(G–L)** Biosynthesis of unsaturated fatty acids-related metabolite changes. *P < 0.05, **P < 0.01.

As shown in **Figures 3E, F**, on both Day 1 and Day 7, the increase in bile acid-related metabolites in the 2 mg BID group was greater than that in the placebo group. Heatmaps (**Figures 3G, H**) also showed the clustering of bile acid-related metabolites together, indicating that they had similar trends of change. Furthermore, as shown in **Figures 4A–C**, the upregulation of three primary bile acids (CA, GCA, and TCA) was statistically significant between the 2 mg BID group and the placebo group at 1 h or 2 h after administration on Days 1 and 7. Other secondary bile acids (DCA, GDCA, and TDCA) showed the same trend (**Figures 4D–F**). These changes suggested that the hypoglycaemic effect of SY-009 might be closely related to changes in the bile acid profile.

However, the metabolites involved in the biosynthesis of unsaturated fatty acids were downregulated in both the 2 mg BID group and the placebo group, and the decrease was not statistically significant (**Figures 4G–L**). Therefore, the changes in this metabolic pathway were not considered an effect of taking SY-009 capsules, and the same was true for several other metabolic pathways associated with unsaturated fatty acids.

In addition, as shown in **Figure 2** and **Figures 3G, H**, other metabolic pathways, including steroid hormone biosynthesis, purine metabolism, and phenylalanine, tyrosine and tryptophan biosynthesis, also differed between the 2 mg BID group and the placebo group. The level of cholesterol sulfate, which is involved in steroid hormone biosynthesis, was significantly greater in the 2 mg BID group than in the placebo group. Allantoin, a metabolite involved in purine metabolism, was significantly upregulated in the placebo group compared with the 2 mg BID group. Phenylalanine, tyrosine and tryptophan biosynthesis was the specific pathway of the placebo group, and the metabolite involved in this pathway, L-phenylalanine, was significantly upregulated.

The differentially abundant metabolite tables (**Supplementary Tables S6**-**S13**), metabolic pathway enrichment plots (**Supplementary Figures S5**, **S6A, B**), and Venn diagrams (**Supplementary Figures S6C, D**) of the other dose groups, including 0.5 mg BID, 1 mg BID, 1 mg QD, and 2 mg QD, revealed that no special important pathways or metabolites were present. Therefore, on the basis of the results of untargeted metabolomics, we comprehensively considered bile acids as our target metabolites for accurate quantitation, focusing on the effects of SY-009 on the bile acid profile.

### 3.2 SY-009 significantly altered the bile acid profile according to targeted metabolomics

#### 3.2.1 SY-009 caused increases in bile acids in patients with T2DM

**Figure 5:**
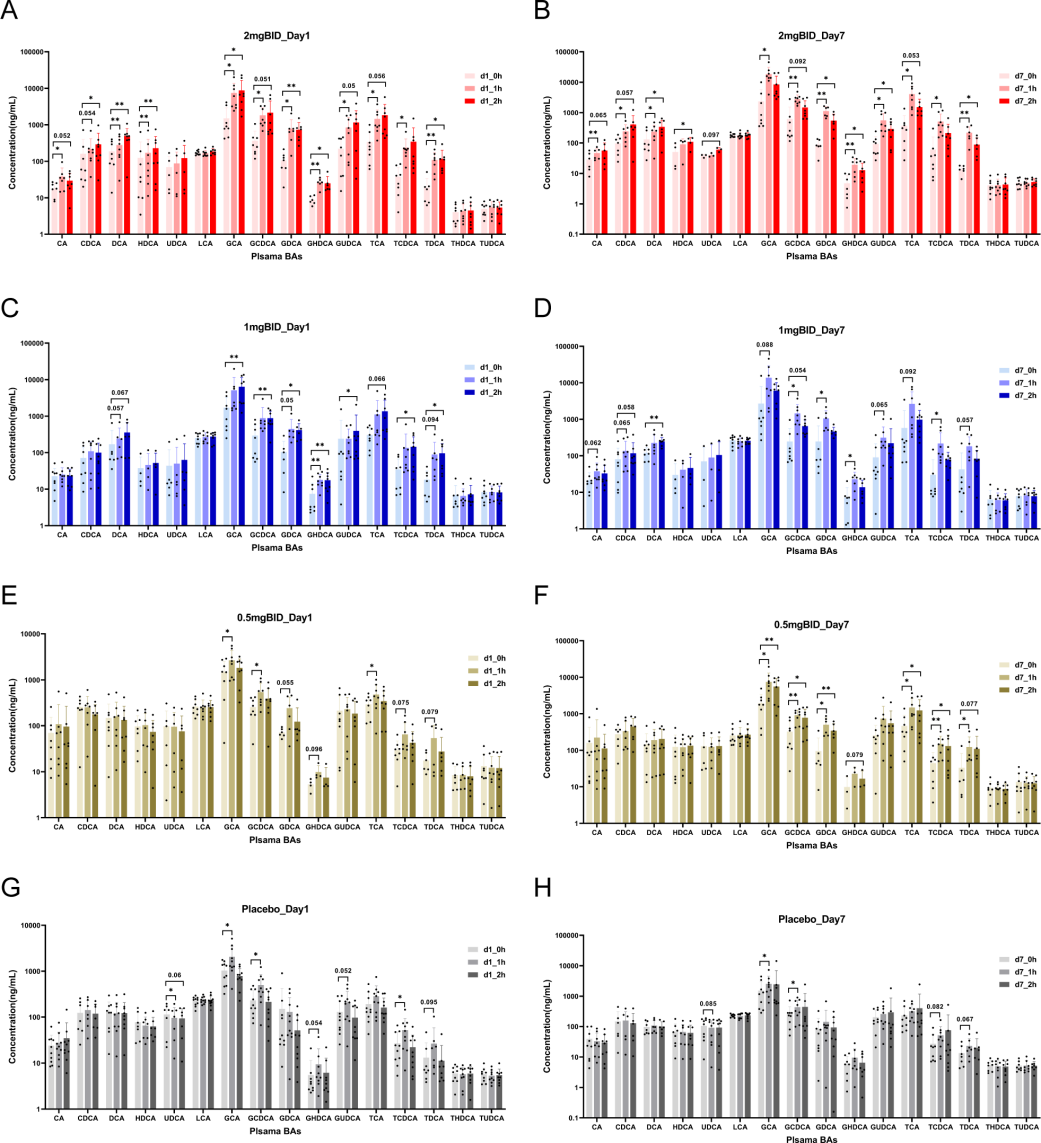
On day 1 or day 7, the changes of bile acids before and after administration in 2 mg BID group, 1 mg BID group, 0.5 mg BID group, and placebo group. **(A)** 2 mg BID_Day 1; **(B)** 2 mg BID_Day 7; **(C)** 1 mg BID_Day 1; **(D)** 1 mg BID_Day 7; **(E)** 0.5 mg BID_Day 1; **(F)** 0.5 mg BID_Day 7; **(G)** Placebo_Day 1; **(H)** Placebo_Day 7. *P < 0.05, **P < 0.01, ns P≥0.05.

For the targeted metabolomics analysis, a representative chromatogram of bile acids is shown in **Supplementary Figure S7A**. After quantitative analysis of the common bile acids of all the samples via LC-MS/MS, as shown in **Figure 5**, in the 2 mg BID group, CA, CDCA, DCA, HDCA, GCA, GCDCA, GDCA, GHDCA, GUDCA, TCA, TCDCA, and TDCA significantly increased 1 h or 2 h after drug administration, whereas UDCA, LCA, THDCA, and TUDCA did not significantly change. Moreover, in the placebo group, GCA, GCDCA, and TCDCA significantly increased on Day 1 and Day 7. The levels of some bile acids also slightly but not significantly increased. In addition, only UDCA was downregulated in the placebo group, but there was a trend towards recovery after SY-009 capsules were taken. The changes in the 1 mg BID group and 0.5 mg BID group tended to be similar to those in the 2 mg BID group, but the degree of change was not as large as that in the 2 mg BID group. The changes in the 1 mg QD and 2 mg QD groups after drug treatment are shown in **Supplementary Figures S7B–E**, and no special conditions were observed.

**Figure 6:**
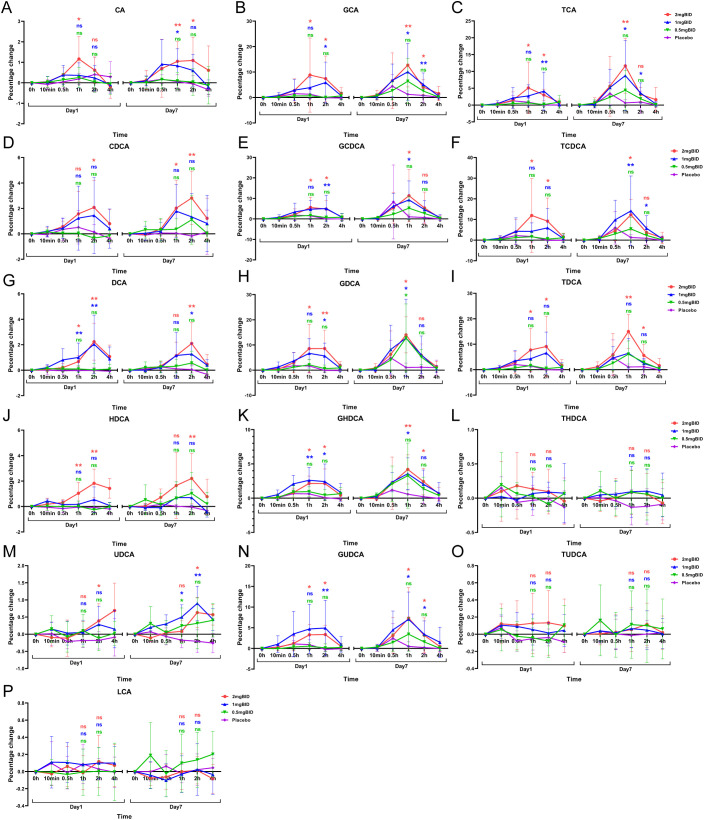
Percentage change from baseline after administration of 16 bile acids: CA**(A)**, GCA**(B)**, TCA**(C)**, CDCA**(D)**, GCDCA**(E)**, TCDCA**(F)**, DCA**(G)**, GDCA**(H)**, TDCA**(I)**, HDCA**(J)**, GHDCA**(K)**, THDCA**(L)**, UDCA**(M)**, GUDCA**(N)**, TUDCA**(O)**, LCA**(P)**. *P < 0.05, **P < 0.01, ns P≥0.05. The LSD method was used to perform multiple comparisons.

The BID dose group and the placebo group were subsequently selected to plot the percentage change in bile acid levels compared with the baseline levels (**Figure 6**). The results revealed that the levels of LCA, THDCA, and TUDCA in each SY-009 group remained stable after administration, with no significant difference compared with those in the placebo group. However, CA, CDCA, DCA, HDCA, UDCA, GCA, GCDCA, GDCA, GHDCA, GUDCA, TCA, TCDCA, and TDCA increased after treatment and peaked at 1 h or 2 h, with the degree of increase being dose-dependent. The increases in these bile acids in the 2 mg BID group was significantly greater than that in the placebo group. Some bile acids in the 1 mg BID group also showed a similar effect to that of the 2 mg BID group or even slightly exceeded it. This might have been due to individual differences, or the 1 mg BID dose possibly achieve maximum efficacy in certain cases. Furthermore, some bile acids tended to increase on Day 7 compared with Day 1, which might be related to continuous medication.

#### 3.2.2 SY-009 caused a decrease in free bile acids and an increase in glycine-conjugated bile acids and PBA/SBA

**Figure 7:**
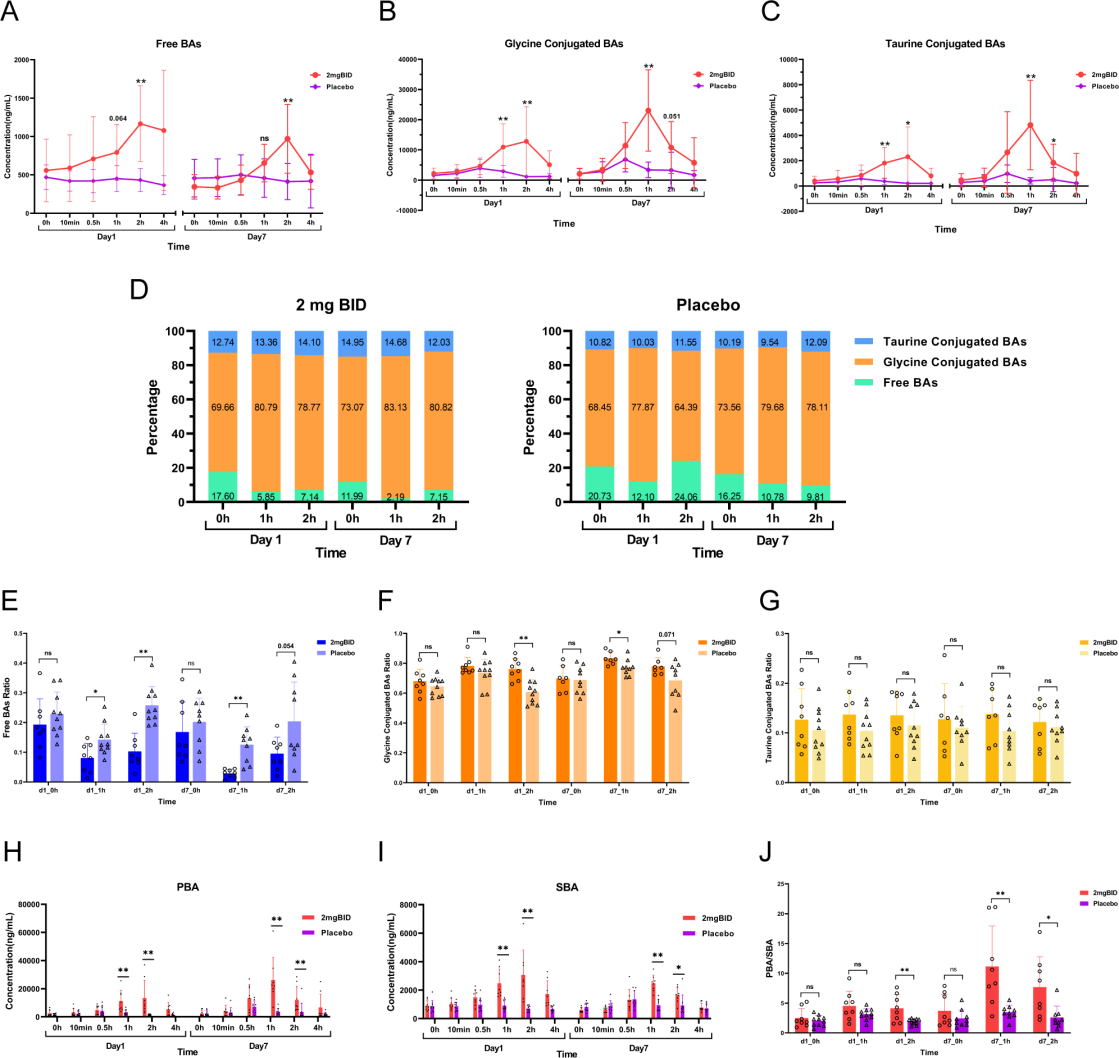
**(A–C)** Changes and differences in the concentration of free bile acids, glycine-conjugated bile acids, and taurine-conjugated bile acids in the 2 mg BID group and placebo group. **(D)** Changes in the ratio of free bile acids, glycine-conjugated bile acids, and taurine-conjugated bile acids in the 2 mg BID group and placebo group. **(E–G)** Difference in proportion of three types of bile acids between the 2 mg BID group and the placebo group. **(H, I)** Changes and differences in the concentration of PBA and SBA in the 2 mg BID group and placebo group. **(J)** The difference of PBA/SBA between the 2 mg BID group and the placebo group. *P < 0.05, **P < 0.01, ns P≥0.05.

The proportion of bile acids in the 2 mg BID group and placebo group were subsequently analysed. **Figures 7A–C** shows that after administration, the levels of free bile acids, glycine-conjugated bile acids, and taurine-conjugated bile acids were significantly greater in the 2 mg BID group than in the placebo group. However, regarding the proportions of the three types of bile acids, in both the 2 mg BID group and the placebo group, the proportion of free bile acids in the total bile acid content decreased, the proportion of glycine-conjugated bile acids increased, and the proportion of taurine-conjugated bile acids tended to remain stable (**Figure 7D**). In addition, the degree of decrease in free bile acid and the degree of increase in glycine-conjugated bile acid in the 2 mg BID group were significantly greater than those in the placebo group, and there was no significant difference in the taurine-conjugated bile acid ratio between the two groups (**Figures 7E–G**).

Moreover, the concentrations of primary bile acid (PBA) and secondary bile acid (SBA) in the 2 mg BID group were significantly greater than those in the placebo group at 1 h or 2 h after administration (**Figures 7H, I**). Furthermore, the PBA/SBA ratio in the 2 mg BID group also increased, and the degree of increase in the ratio was significantly greater than that in the placebo group, especially on Day 7 (**Figure 7J**).

#### 3.2.3 Bile acid profiles are strongly correlated with the T2DM phenotype

**Figure 8:**
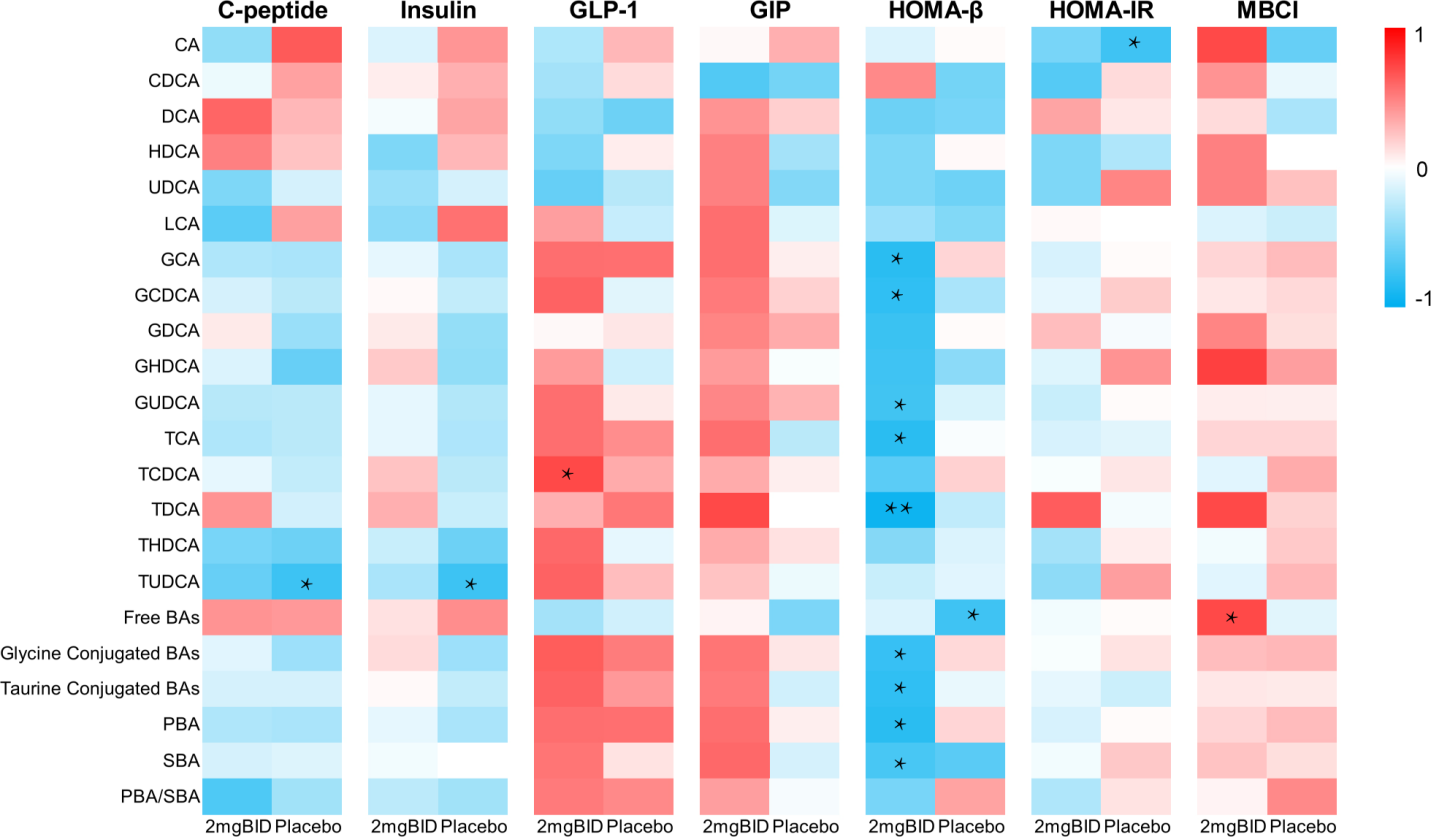
On day 7, spearman correlation analysis of percentage changes of various bile acids at 2h after administration with C-peptide and other indicators. *P < 0.05, **P < 0.01.

Preliminary clinical studies have shown that SY-009 can reduce postprandial blood glucose but does not affect fasting blood glucose (22). Data on C-peptide and other indices related to T2DM have been obtained. Changes in these indices were also accompanied by changes in bile acid levels. Therefore, we selected the data 2 hours after the seventh day of administration and analysed the correlation between the percentage change in bile acid levels compared with baseline levels and the change in these indices. The results revealed that the free or conjugated status of bile acids in the bile acid pool was closely related to HOMA-β (**Figure 8**).

## 4 Discussion

Metabolomics can reflect the dynamic changes in metabolites throughout the body. T2DM is a common metabolic disease, and investigating the pathophysiological and metabolic regulatory mechanisms of T2DM using metabolomics technology is highly important. Therefore, this study was based mainly on untargeted and targeted metabolomics technologies to analyse subjects’ plasma samples obtained from patients in the SY-009 phase Ib clinical study, aiming to reveal the effect of SY-009 on T2DM.

In the untargeted metabolomics analysis, after SY-009 administration, a series of metabolic pathways, including primary bile acid biosynthesis, biosynthesis of unsaturated fatty acids, steroid hormone biosynthesis, purine metabolism, and phenylalanine, tyrosine and tryptophan biosynthesis, were altered. Among them, the unsaturated fatty acid-related metabolic pathways and the bile acid-related metabolic pathways in the 2 mg BID group were among the key metabolic pathways enriched. However, there were no significant differences in unsaturated fatty acids, including palmitic acid, oleic acid, linoleic acid, alpha-linolenic acid, docosahexaenoic acid, or arachidonic acid, between the 2 mg BID group and the placebo group, suggesting that the changes in these unsaturated fatty acids may not be attributable to the effects of SY-009. Unlike the increase in unsaturated fatty acids, the increase in bile acids, including CA, GCA, TCA, DCA, GDCA, and TDCA, in the 2 mg BID group was significantly greater than that in the placebo group. Many studies have shown a close correlation between bile acids and T2DM. For example, Alessandro Mantovani et al. reported significant differences in bile acid profiles between T2DM patients and non-T2DM patients (25). Bingting Chen et al. revealed that GUDCA may play a hypoglycaemic role by regulating bile acid metabolism (26). With respect to other metabolic pathways affected by SY-009, some relevant studies have investigated the metabolites involved in these pathways. The level of cholesterol sulfate involved in the steroid hormone biosynthesis pathway was significantly greater in the 2 mg BID group than in the placebo group. Cholesterol sulfate, which is abundant in the gut (27), is an important regulatory molecule and acts as an endogenous regulator of cholesterol synthesis (28). Dongke Xu et al. reported that cholesterol sulfate promotes cholesterol synthesis in colon epithelial cells to relieve ulcerative colitis (29). Bile acids are produced by cholesterol, and the changing trend of cholesterol sulfate is consistent with that of bile acids, suggesting that the change in bile acids may be related to the promotion of cholesterol sulfate by SY-009. In addition, Xueping Zhang et al. proposed that cholesterol sulfate may have a protective effect on β-cells (30). Therefore, the increase in cholesterol sulfate caused by SY-009 may be important. With respect to the metabolic pathways of phenylalanine, tyrosine and tryptophan biosynthesis, the increase in phenylalanine in the placebo group was mitigated to some extent by SY-009. In previous studies, phenylalanine-related aromatic amino acids have been reported as potential biomarkers of T2DM in several studies (21, 31, 32). Qian Zhou et al. reported that phenylalanine impairs insulin signalling and inhibits glucose uptake by modifying insulin receptor beta (33). These studies suggest that SY-009 may ameliorate T2DM by affecting the metabolism of aromatic amino acids. In addition, the level of allantoin in the placebo group was significantly greater than that in 2 mg BID group. However, both Junnan Ma et al. (34) and Hyeon-Kyu Go et al. (35) reported the antiglucose effect of allantoin on diabetic mice. This contradicts our experimental results and requires further consideration.

Given the limited number of plasma samples, bile acids were selected as our targeted quantitative metabolites to reveal the effect of SY-009 on the bile acid profile because bile acids play an important role in regulating systemic metabolism and inflammation to affect diabetes, and they are key regulators and novel treatment targets in T2DM (36). In particular, GUDCA can regulate bile acid levels and alter gut microbiota to attenuate diabetes (26). Moreover, modulating bile acids is also a mechanism by which metformin exerts its glucose-lowering effect (37). Thus, targeting bile acid metabolism is a potential strategy for treating T2DM.

In the targeted metabolomics results, after SY-009 administration, in addition to LCA, THDCA, and TUDCA, other bile acids, including CA, CDCA, DCA, HDCA, UDCA, GCA, GCDCA, GDCA, GHDCA, GUDCA, TCA, TCDCA, and TDCA were significantly increased compared with those in the placebo group, and these increases were dose-dependent. In addition, in the whole bile acid pool, the proportions of free, glycine-conjugated, and taurine-conjugated bile acids also changed greatly. Compared with those in the placebo group, in the SY-009 group, the proportion of free types decreased significantly, the proportion of glycine-conjugated types increased significantly, and the proportion of taurine-conjugated types tended to be stable. Moreover, PBA/SBA also showed a significant upward trend.

At present, increasing evidence shows that the coregulation of the intestinal flora and bile acids may mediate host metabolism and affect the occurrence and development of diabetes (26, 38, 39). In the bile acid synthesis pathway in humans, the metabolism of cholesterol produces primary bile acids, including CA and CDCA, in the liver. Besides, free bile acids are usually combined with glycine or taurine to form conjugated bile acids. Then, the primary bile acids enter the intestine and are deconjugated by bacterial bile salt hydrolase (BSH) and dehydroxylated by bacterial 7a-dehydroxylase to form DCA and LCA. A small amount of CDCA is converted to UDCA, and LCA can be converted to HDCA by intestinal 6α- and 6β-hydroxylase (40).

According to the results of our study, the increase in primary bile acid content after the consumption of SY-009 suggests that SY-009 may promote the conversion of cholesterol to primary bile acids. In addition, DCA significantly increased after drug treatment, suggesting that BSH and 7α-dehydroxylase are likely to be activated. The stable trend of LCA after taking the drug may be due to the inhibition of other unknown channels or because LCA is conjugated with sulfate and excreted in the stool and urine rather than entering the bloodstream (Chiang and Ferrell 2020). The intestinal environment has a strong influence on bile acid deconjugation and conjugation. Besides, BSH activity is restricted to the genera *Clostridium*, *Enterococcus*, *Bifidobacterium*, *Bacteroides*, and *Lactobacillus*, while 7α-dehydroxylase activity mainly originates from Clostridium XIVa clusters (41, 42). All BSH reactions rely on the hydrolysis of amide bonds to release taurine or glycine (43–46). Moreover, the intestinal flora has different preferences for glycine-conjugated BAs and taurine-conjugated BAs. For example, in *Bifidobacterium*, three types of BSH have been found, two of which have high activity. They all prefer glycine-conjugated BAs to taurine-conjugated BAs (47). Moreover, after SY-009 capsules were taken, secondary bile acids, including HDCA and UDCA, tended to bind to glycine rather than taurine and were associated with the gut microbiota. In addition, the significant increase in PBA/SBA is also worth paying attention to. At present, studies in other fields have shown the importance of changes in the bile acid ratio. For example, Tianlu Chen et al. chose the bile acid glycine: taurine ratio as a biomarker to monitor the progression of liver disease (48). Tingting Gao et al. reported that the primary/secondary bile acid ratio was a serum diagnostic marker for the need for surgery in infants with necrotizing enterocolitis (49). Taken together, although the effect and mechanism of the change in the plasma bile acid ratio after SY-009 administration have not been clarified, modulating the gut microbiota composition may be a possible mechanism by which SY-009 affects diabetes.

Importantly, the nuclear farnesoid X receptor (FXR) and the membrane Takeda G protein-coupled receptor 5 (TGR5) are currently the most important receptors mediating the regulation of bile acids (50). The effect of intestinal FXR on liver metabolism was first reported by Sayin et al. (51). Yangfeng Hou et al. also conducted a systematic review of the multi-pathway regulation of blood glucose by FXR (52). The important role of TGR5 in the occurrence and development of metabolic syndrome, such as diabetes, has also been reviewed and reported by Xianmei Gou et al. (53). Therefore, whether SY-009 has an effect on FXR and TGR5 is a future research direction.

SY-009, a novel SGLT1 inhibitor, is difficult to absorb orally, and SGLT1 receptors are abundantly distributed in the intestine (4). In the phase Ib clinical study, after SY-009 was taken, the drug concentration in plasma was below the limit of quantitation (LOQ), and PK parameters were not available (22). The characteristic of unrelated PK/PD may be that SY-009 is not directly absorbed into the digestive tract and instead acts on SGLT1 locally. Our results showed that SY-009 significantly modulated the composition of bile acids. There is a bidirectional relationship between the intestinal microbial community and bile acids (54). The microbial flora closely regulates the metabolism and synthesis of bile acids, and the composition of the bile acid pool affects the diversity and homeostasis of the intestinal flora. However, whether SY-009 can directly modulate the activities of enzymes responsible for cholesterol metabolism and bile acid synthesis or regulate the intestinal flora is unclear. The results of our metabolomics study suggest that the coregulation of bile acids and the intestinal flora may be an important part of the hypoglycaemic mechanism of SY-009. Further studies can be conducted in the future if there is an opportunity to obtain stool samples from patients after taking the drug.

Considering various conditions, such as manpower, material resources, and limited blood samples, we only quantified bile acids based via targeted metabolomics, which is a limitation of this study. However, the untargeted metabolomics results suggest that SY-009, and even other SGLT1 inhibitors, may affect on more metabolic pathways related to T2DM, which needs to be clarified. Furthermore, the specific mechanisms of SY-009 need to be further investigated.

## 5 Conclusion

In summary, our results revealed that there are significant changes in plasma metabolomics in patients with T2DM after taking SY-009 capsules. In particular, a series of bile acids, including CA, CDCA, DCA, HDCA, UDCA, GCA, GCDCA, GDCA, GHDCA, GUDCA, TCA, TCDCA, and TDCA, were increased significantly, and the bile acid ratios, including free bile acids, glycine-conjugated bile acids and PBA/SBA, were also significantly affected by SY009. These changes in the bile acid profile provide a new perspective on the hypoglycaemic effect of SY-009. This study is the first application of metabolomics for SGLT1 inhibitors, which is highly important for understanding the pathogenesis, progression, prognosis, and treatment of T2DM.

## Data Availability

The original contributions presented in the study are included in the article/**Supplementary Material**. Further inquiries can be directed to the corresponding author/s.

## Supplementary material

Supplementary Table 1 2 mg BID PLS-DA model parameters

Supplementary Table 2 2 mg BID_day 1: The identification of differential metabolites based on untargeted metabolomics.

Supplementary Table 3 2 mg BID_day 7: The identification of differential metabolites based on untargeted metabolomics.

Supplementary Table 4 Placebo_Day 1: The identification of differential metabolites based on untargeted metabolomics.

Supplementary Table 5 Placebo_Day 7: The identification of differential metabolites based on untargeted metabolomics.

Supplementary Table 6 0.5 mg BID_Day 1: The identification of differential metabolites based on untargeted metabolomics.

Supplementary Table 7 0.5 mg BID_Day 7: The identification of differential metabolites based on untargeted metabolomics.

Supplementary Table 8 1 mg BID_Day 1: The identification of differential metabolites based on untargeted metabolomics.

Supplementary Table 9 1 mg BID_Day 7: The identification of differential metabolites based on untargeted metabolomics.

Supplementary Table 10 1 mg QD_Day 1: The identification of differential metabolites based on non-targeted metabolomics.

Supplementary Table 11 1 mg QD_Day 7: The identification of differential metabolites based on untargeted metabolomics.

Supplementary Table 12 2 mg QD_Day 1: The identification of differential metabolites based on untargeted metabolomics.

Supplementary Table 13 2 mg QD_Day 7: The identification of differential metabolites based on untargeted metabolomics.
