# Supplementary material for: Effect of SY009, a novel SGLT1 inhibitor, on the plasma metabolome and bile acids in patients with type 2 diabetes mellitus
Source: Front Endocrinol (Lausanne). 2025 Jan 28;16:1487058. doi: 10.3389/fendo.2025.1487058 (PMC11810745; doi:10.3389/fendo.2025.1487058)
Supplement: Supplementary Table 1 — 2 mg BID PLS-DA model parameters [file DataSheet1.docx]

Supplementary Material

# Supplementary Figures and Tables

## Supplementary Figures


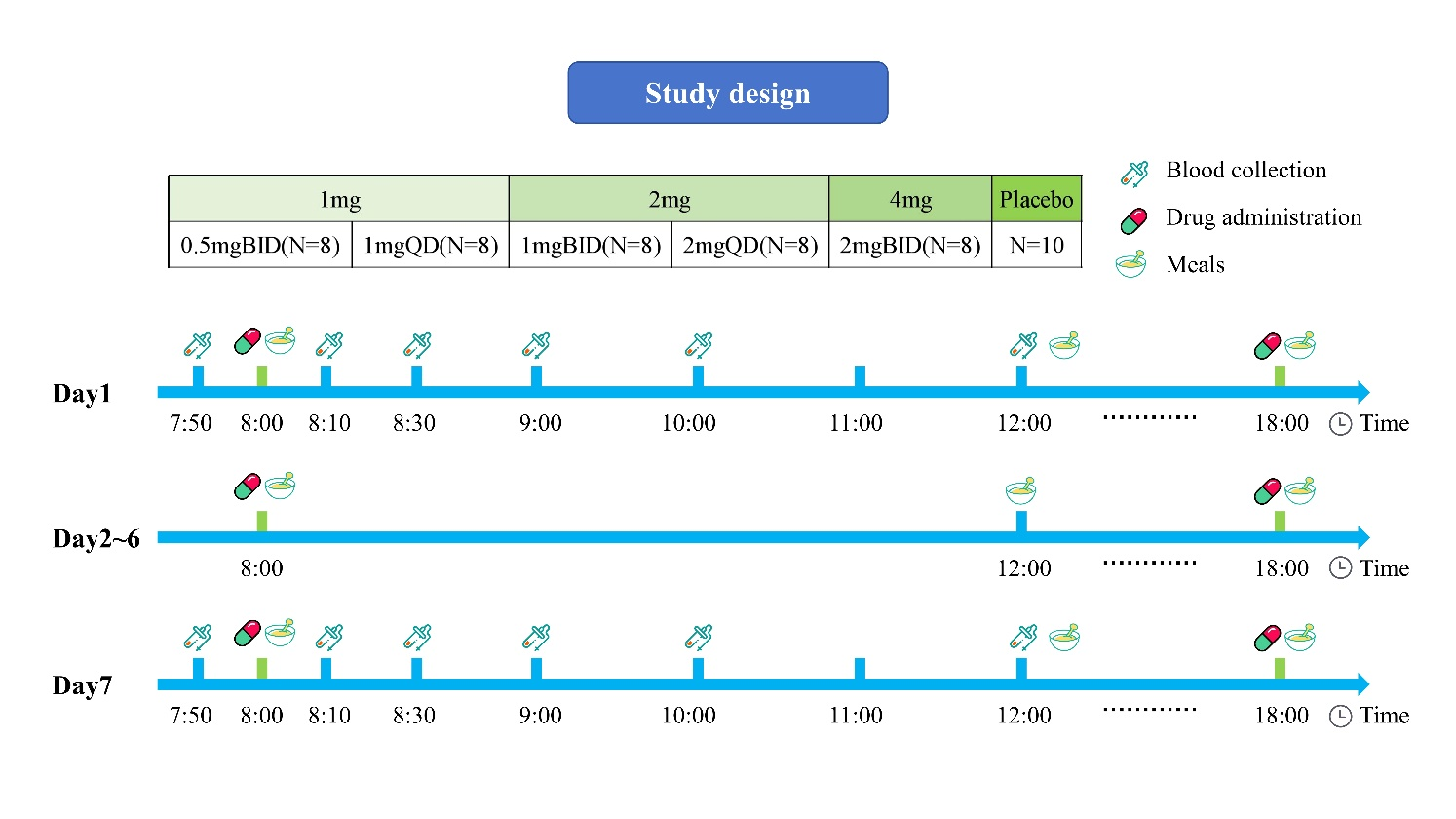


**Supplementary Figure S1.** Schematic diagram of the clinical study design including dose design, meal and medication time of subjects, and PK blood collection time.


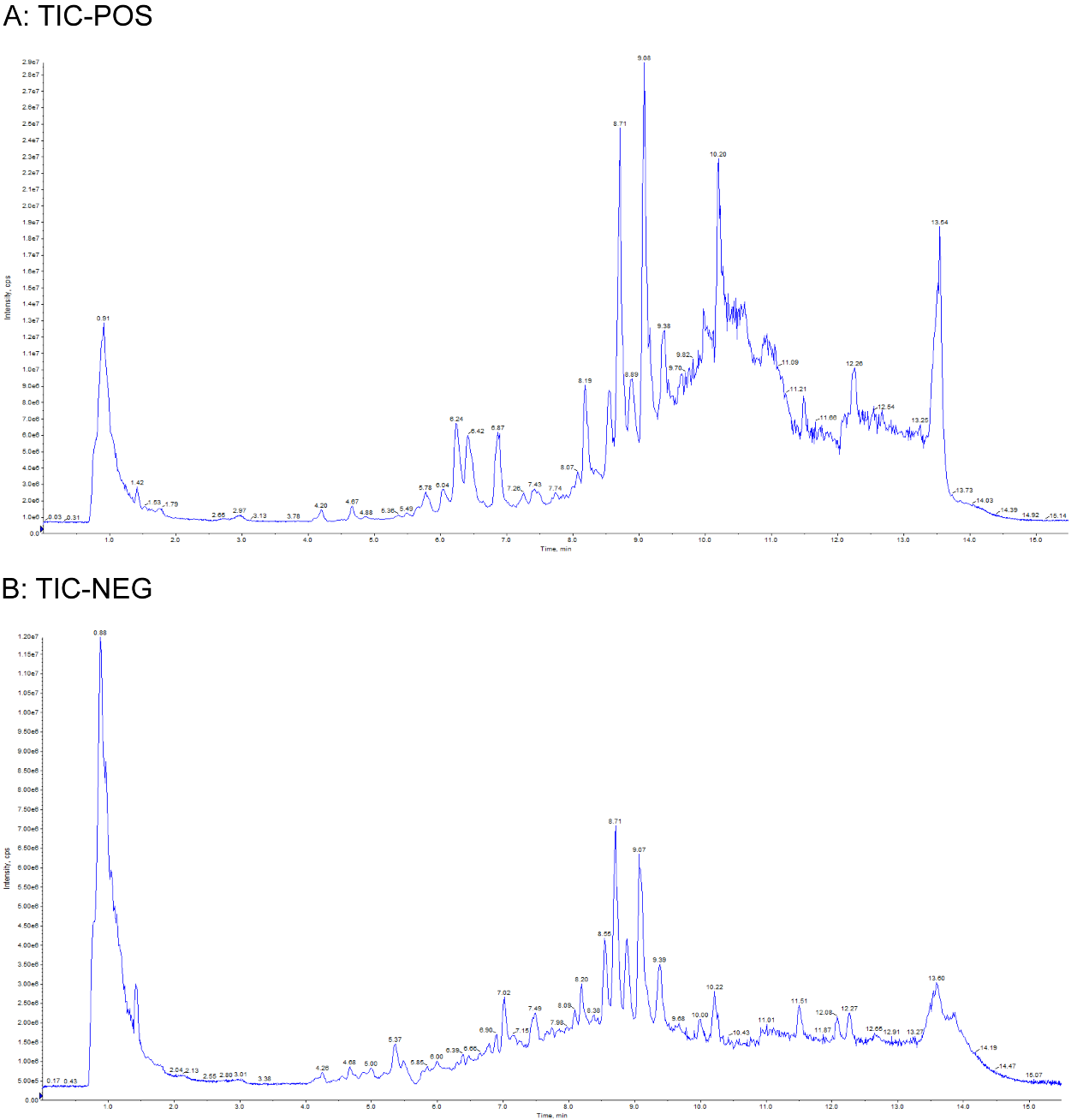


**Supplementary Figure S2.** The representative total ion current chromatograms obtained by LC-TOF-MS in positive and negative ion modes.


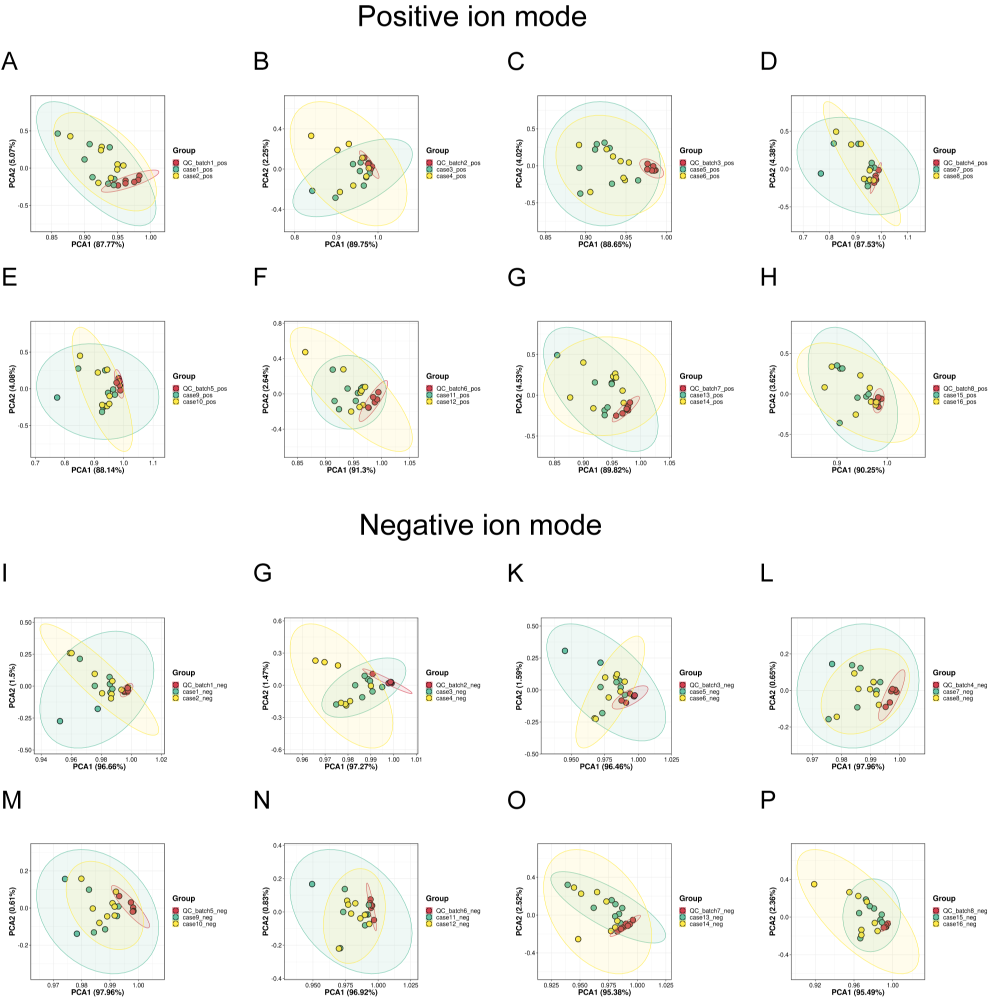


**Supplementary Figure S3.** PCA diagram for 8 sample batches. The QC samples are represented by pink, while the samples before and after SY009 administration are represented by blue and orange, respectively. The ellipse represents a 95% confidence interval. Note: Figures A-H correspond to ESI (+), while Figures I-P correspond to ESI (-).


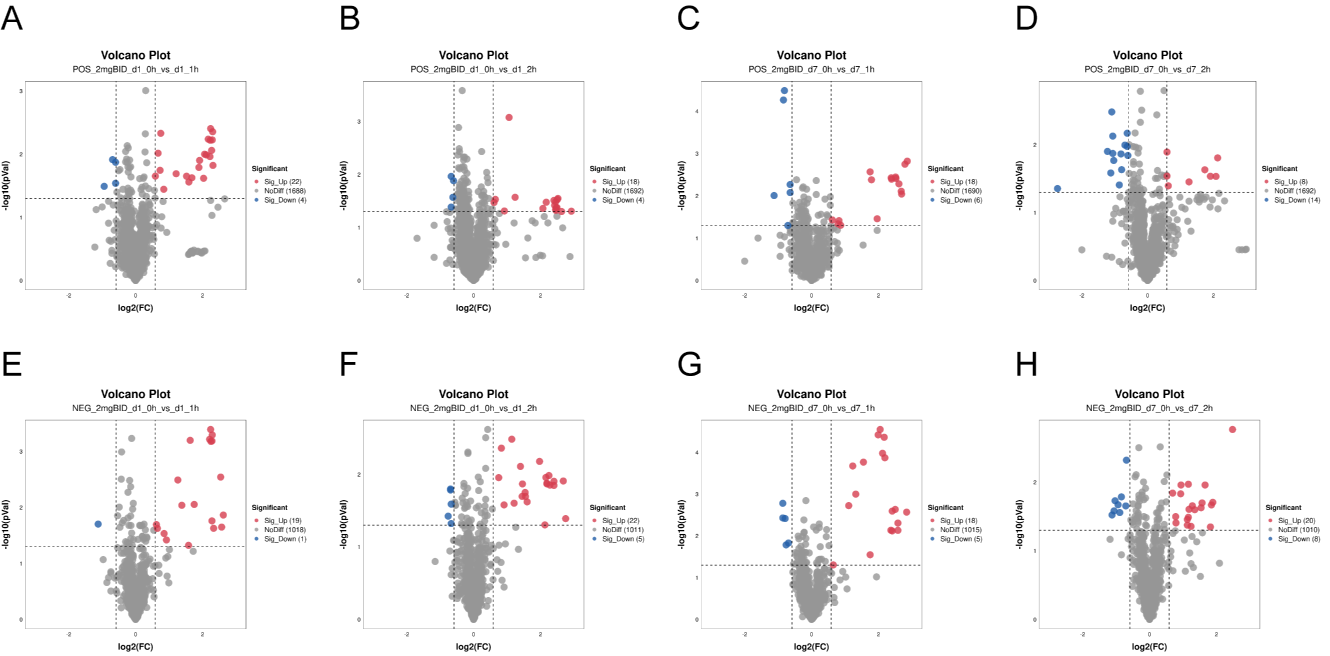


**Supplementary Figure S4.** A-D: volcano plots in positive ion mode. E-H: volcano plots in negative ion mode. Red dots represent up-regulated metabolites with FC>1.5 and P<0.05, and blue dots represent down-regulated metabolites with FC<0.67 and P<0.05.


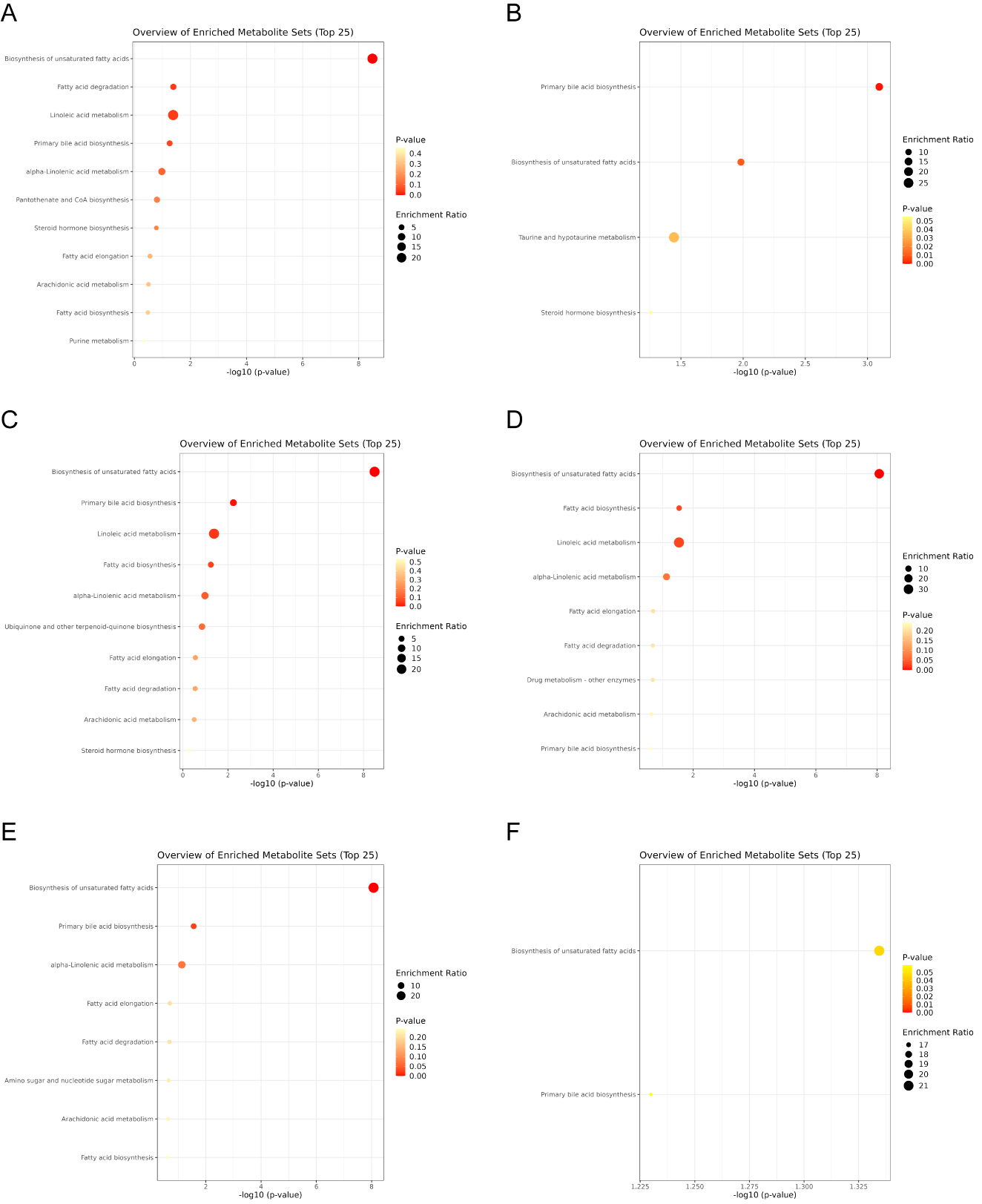


**Supplementary Figure S5.** The metabolic pathway enrichment diagrams of SY-009 groups on day 1 and day 7. A: 0.5 mg BID_Day 1; B: 0.5 mg BID_Day 7; C: 1 mg BID_Day 1; D: 1 mg BID_Day 7; E: 1 mg QD_Day 1; F: 1 mg QD_Day 7.

**
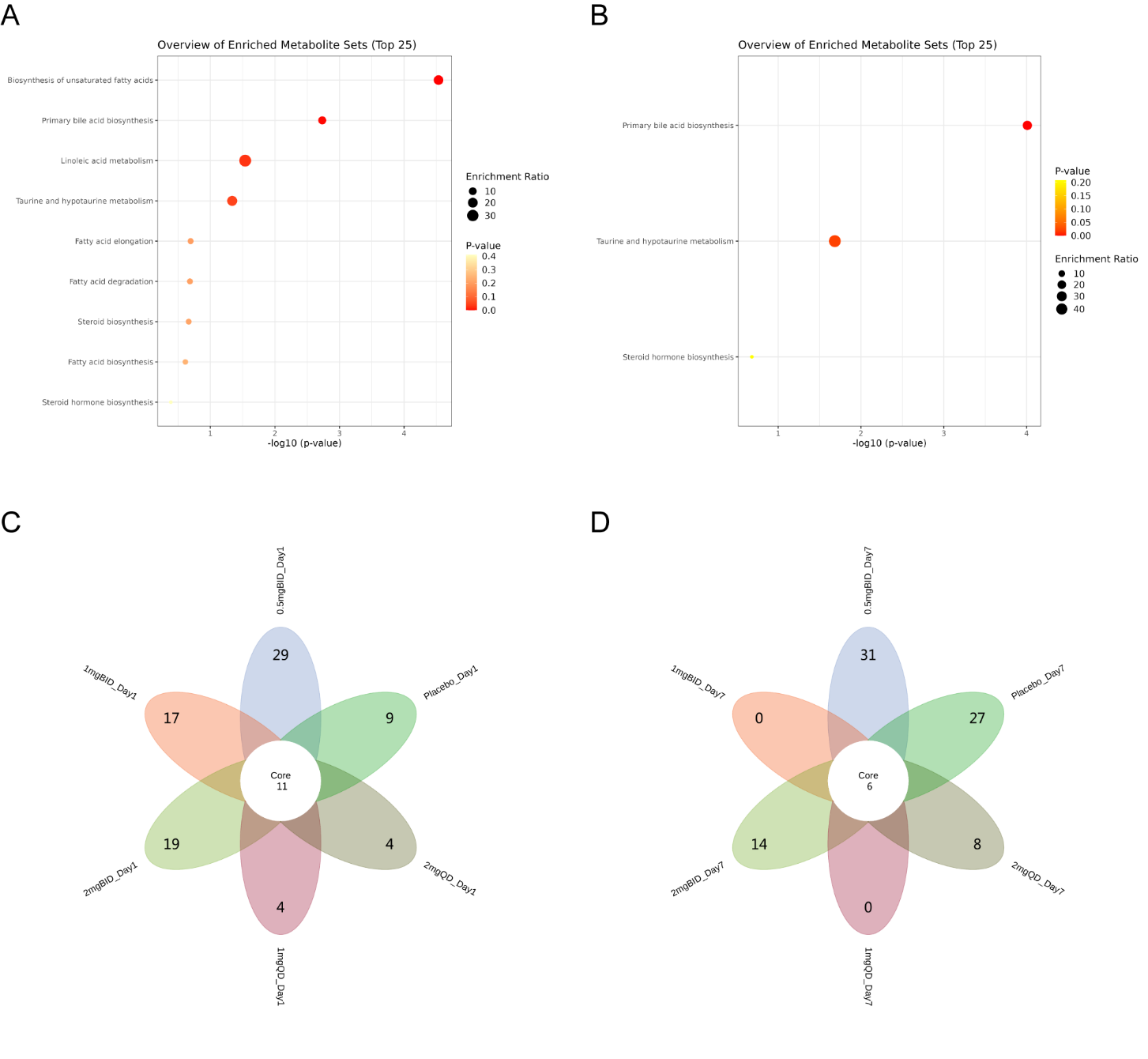
**

**Supplementary Figure S6.** A-B: The metabolic pathway enrichment diagrams of 2 mg QD groups on day 1 and day 7. A: 2 mg QD_Day 1; B: 2 mg QD_Day 7. C-D: The Venn diagrams of all dose groups on day 1 and day 7. C: Day1; D: Day7.


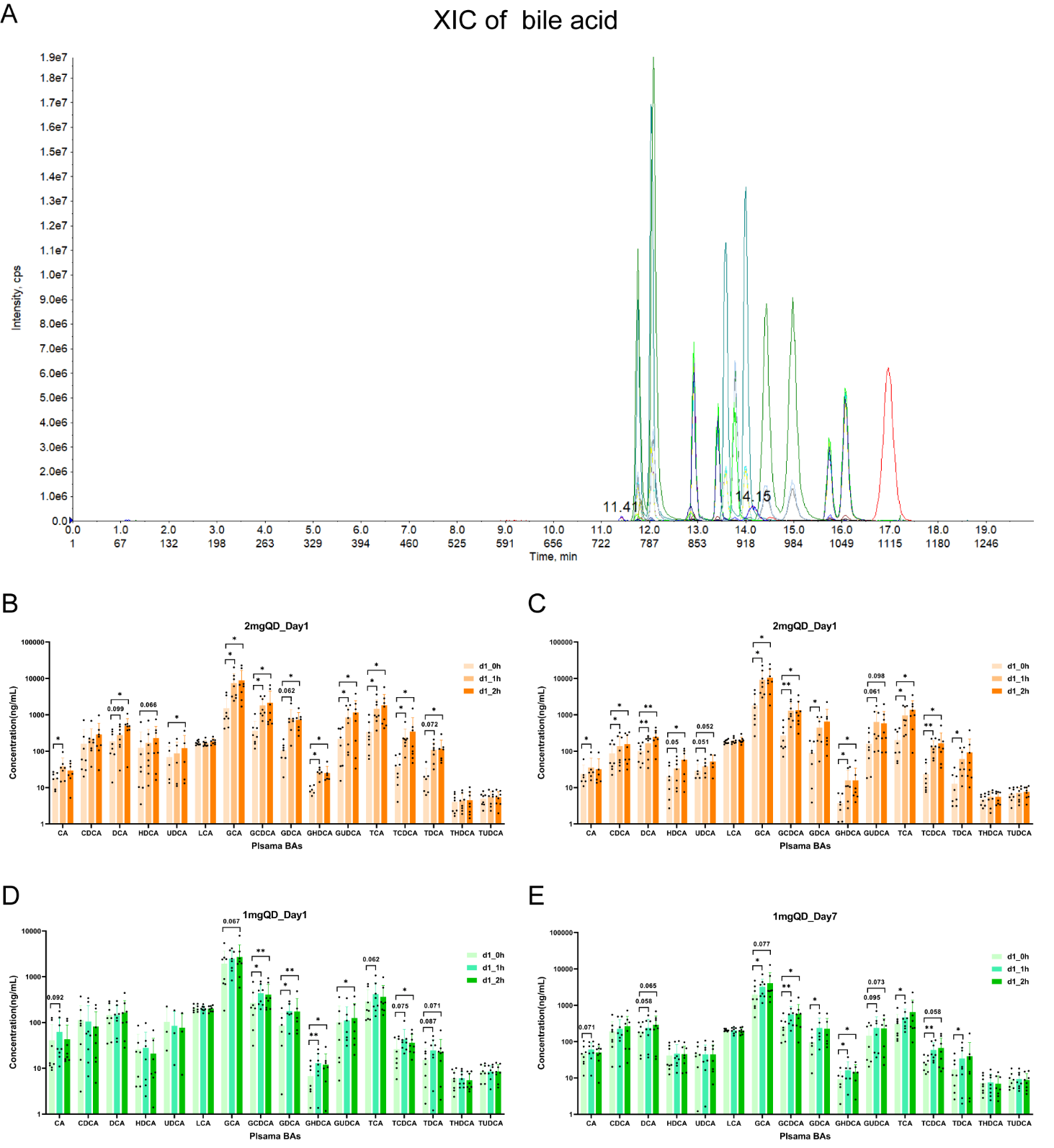


**Supplementary Figure S7.** A: The representative chromatogram of bile acids based on targeted metabolomics analysis. B-E: Changes of different bile acids in 1 mg QD and 2 mg QD groups before and after administration. B: 2 mg QD_Day 1; C: 2 mg QD_Day 7; D: 1 mg QD_Day 1; E: 1 mg QD_Day 7. *: P < 0.05, **: P < 0.01.

## Supplementary Tables

**Supplementary Table S1.** 2 mg BID PLS-DA model parameters

| Ion mode | Group comparison | R^2^ | Q^2^ | P value R^2^ | P value Q^2^ | Intercept R^2^ | Intercept Q^2^ |
| --- | --- | --- | --- | --- | --- | --- | --- |
| ESI(+) | d1_0h_vs_d1_1h | 0.867268703 | 0.233962088 | 0.08 | 0.04 | 0.752080749 | -0.595072019 |
| ESI(+) | d1_0h_vs_d1_2h | 0.831120578 | 0.113342621 | 0.36 | 0.02 | 0.788672537 | -0.588145916 |
| ESI(+) | d7_0h_vs_d7_1h | 0.879089163 | 0.40940916 | 0.07 | 0.005 | 0.710178775 | -0.825483922 |
| ESI(+) | d7_0h_vs_d7_2h | 0.914319706 | 0.285174082 | 0.025 | 0.025 | 0.747600271 | -0.710990896 |
| ESI(-) | d1_0h_vs_d1_1h | 0.915291087 | 0.270794707 | 0.035 | 0.015 | 0.780606371 | -0.617408054 |
| ESI(-) | d1_0h_vs_d1_2h | 0.890715113 | 0.259065356 | 0.06 | 0.025 | 0.764956448 | -0.738213147 |
| ESI(-) | d7_0h_vs_d7_1h | 0.891489041 | 0.562688725 | 0.055 | 0.001 | 0.751792274 | -0.733203203 |
| ESI(-) | d7_0h_vs_d7_2h | 0.918185623 | 0.439289351 | 0.02 | 0.015 | 0.788602407 | -0.645568758 |

**Supplementary Table S2.** 2 mg BID_day 1: The identification of differential metabolites based on untargeted metabolomics.

| NO | Ion mode | Identification | mz | rt | Adduct | Database | d1_1h vs d1_0h | | |  | d1_2h vs d1_0h | | |
| --- | --- | --- | --- | --- | --- | --- | --- | --- | --- | --- | --- | --- | --- |
|  |  |  |  |  |  |  | VIP | FC | Trend |  | VIP | FC | Trend |
| 1 | ESI(+) | Yessotoxin | 1160.534 | 383.804 | (M+NH4)+ | KEGG | 2.44 | 4.21 | ↑ |  | 2.23 | 4.16 | ↑ |
| 2 | ESI(+) | GDP-4-dehydro-3,6-dideoxy-alpha-D-mannose | 1160.133 | 384.763 | (2M+NH4)+ | KEGG | 2.70 | 3.35 | ↑ |  | - | - | - |
| 3 | ESI(+) | Ginsenoside Rb1 | 1150.664 | 385.179 | (M+CH3CN+H)+ | KEGG | 1.52 | 3.00 | ↑ |  | - | - | - |
| 4 | ESI(+) | (4Z,7Z,10Z,13Z,16Z,19Z)-Docosahexaenoyl-CoA | 1122.321 | 373.479 | (M-H+2Na)+ | KEGG | 2.54 | 3.15 | ↑ |  | - | - | - |
| 5 | ESI(+) | Naphthyl-2-oxomethyl-succinyl-CoA | 1088.127 | 383.808 | (M-2H+3Na)+ | KEGG | 1.84 | 3.02 | ↑ |  | - | - | - |
| 6 | ESI(+) | 3-Oxo-OPC8-CoA | 1080.310 | 384.247 | (M+Na)+ | KEGG | 2.29 | 4.11 | ↑ |  | - | - | - |
| 7 | ESI(+) | Oxytocin | 1024.473 | 383.862 | (M+NH4)+ | KEGG | 2.21 | 3.89 | ↑ |  | 2.39 | 3.98 | ↑ |
| 8 | ESI(+) | Icosenoyl-CoA | 1024.414 | 383.813 | (M+H-2H2O)+ | KEGG | 2.08 | 3.30 | ↑ |  | - | - | - |
| 9 | ESI(+) | 3-Oxo-OPC4-CoA | 1024.238 | 383.775 | (M+Na)+ | KEGG | 2.35 | 3.27 | ↑ |  | - | - | - |
| 10 | ESI(+) | 2'-Deoxyinosine triphosphate | 1022.943 | 384.666 | (2M+K)+ | KEGG | 2.10 | 3.28 | ↑ |  | - | - | - |
| 11 | ESI(+) | Undecaprenyl phosphate alpha-L-Ara4N | 1000.734 | 374.341 | (M+Na)+ | KEGG | 2.11 | 0.66 | ↓* |  | 1.81 | 0.65 | ↓* |
| 12 | ESI(+) | Decanoyl-CoA | 966.224 | 384.579 | (M-H+2Na)+ | KEGG | - | - | - |  | 2.02 | 2.92 | ↑ |
| 13 | ESI(+) | Flupoxam | 959.110 | 384.952 | (2M+K)+ | KEGG | 1.70 | 3.05 | ↑ |  | - | - | - |
| 14 | ESI(+) | Zn-Bacteriochlorophyll a | 915.476 | 384.582 | (M+H-2H2O)+ | KEGG | 1.97 | 3.95 | ↑ |  | - | - | - |
| 15 | ESI(+) | 1-(5'-Phosphoribosyl)-5-amino-4-(N-succinocarboxamide)-imidazole | 909.157 | 384.910 | (2M+H)+ | KEGG | 1.80 | 3.86 | ↑ |  | - | - | - |
| 16 | ESI(+) | Glucoberteroin | 909.104 | 384.947 | (2M+K)+ | KEGG | 1.68 | 3.13 | ↑ |  | - | - | - |
| 17 | ESI(+) | 24,25-Dihydrolanosterol | 874.851 | 372.982 | (2M+NH4)+ | KEGG | - | - | - |  | 2.14 | 0.66 | ↓* |
| 18 | ESI(+) | beta-Tocotrienol | 859.601 | 807.898 | (2M+K)+ | KEGG | - | - | - |  | 3.17 | 0.57 | ↓ |
| 19 | ESI(+) | Oxychlordane | 857.586 | 807.605 | (2M+NH4)+ | KEGG | 1.29 | 0.61 | ↓ |  | - | - | - |
| 20 | ESI(+) | Ubiquinone-9 | 812.670 | 813.561 | (M+NH4)+ | KEGG | - | - | - |  | 2.24 | 1.53 | ↑ |
| 21 | ESI(+) | Petromyzonol | 811.603 | 656.376 | (2M+Na)+ | KEGG | 2.96 | 0.52 | ↓* |  | 2.21 | 0.59 | ↓ |
| 22 | ESI(+) | Plastoquinone-9 | 787.604 | 510.075 | (M+K)+ | KEGG | 2.40 | 0.50 | ↓ |  | - | - | - |
| 23 | ESI(+) | Clobenpropit | 617.179 | 434.546 | (2M+H)+ | KEGG | 1.60 | 0.58 | ↓ |  | - | - | - |
| 24 | ESI(+) | 15-Demethoxy-epsilon-rhodomycin | 616.176 | 434.554 | (M-H+2Na)+ | KEGG | 1.47 | 0.60 | ↓ |  | - | - | - |
| 25 | ESI(+) | 5'-S-Methyl-5'-thioinosine | 614.181 | 434.762 | (2M+NH4)+ | KEGG | 1.90 | 0.63 | ↓ |  | - | - | - |
| 26 | ESI(+) | L-Urobilinogen | 597.363 | 460.602 | (M+H)+ | KEGG | 2.02 | 1.77 | ↑ |  | 1.97 | 1.59 | ↑ |
| 27 | ESI(+) | Rocuronium | 596.352 | 460.205 | (M-2H+3Na)+ | KEGG | 2.31 | 1.66 | ↑* |  | - | - | - |
| 28 | ESI(+) | 3-Hydroxy-palmitic acid methyl ester | 595.493 | 809.854 | (2M+Na)+ | KEGG | - | - | - |  | 2.04 | 1.55 | ↑ |
| 29 | ESI(+) | Graphinone | 593.333 | 462.508 | (2M+H)+ | KEGG | 2.45 | 1.79 | ↑* |  | 2.66 | 1.89 | ↑* |
| 30 | ESI(+) | alpha-Tocopherol acetate | 495.379 | 450.388 | (M+Na)+ | KEGG | 7.66 | 4.16 | ↑** |  | 6.42 | 4.49 | ↑* |
| 31 | ESI(+) | Molybdoenzyme molybdenum cofactor | 487.863 | 47.150 | (M+H-2H2O)+ | KEGG | - | - | - |  | 2.74 | 8.24 | ↑ |
| 32 | ESI(+) | Cholic acid | 450.321 | 451.330 | (M+CH3CN+H)+ | KEGG | 7.49 | 4.71 | ↑** |  | 7.05 | 5.75 | ↑* |
| 33 | ESI(+) | 3alpha,7alpha-Dihydroxy-12-oxo-5beta-cholanate | 448.306 | 432.057 | (M+CH3CN+H)+ | KEGG | 3.64 | 2.86 | ↑* |  | 3.31 | 2.37 | ↑* |
| 34 | ESI(+) | Di(2-ethylhexyl) adipate | 417.319 | 477.068 | (M+HCOO+2H)+ | KEGG | 3.03 | 1.68 | ↑** |  | - | - | - |
| 35 | ESI(+) | Sphinganine 1-phosphate | 382.272 | 491.414 | (M+H)+ | KEGG | 2.22 | 0.66 | ↓* |  | - | - | - |
| 36 | ESI(+) | Deoxyadenosine diphosphate | 376.020 | 59.550 | (M+H-2H2O)+ | KEGG | - | - | - |  | 1.95 | 0.63 | ↓* |
| 37 | ESI(+) | 2-Undecanone | 358.368 | 515.588 | (2M+NH4)+ | KEGG | - | - | - |  | 2.94 | 1.53 | ↑* |
| 38 | ESI(+) | Propofol | 357.279 | 520.791 | (2M+H)+ | KEGG | - | - | - |  | 4.13 | 2.08 | ↑** |
| 39 | ESI(+) | Erucic acid | 321.316 | 649.828 | (M+H-H2O)+ | KEGG | - | - | - |  | 1.96 | 0.58 | ↓ |
| 40 | ESI(+) | Tridihexethyl | 319.285 | 652.168 | (M+H)+ | KEGG | - | - | - |  | 1.14 | 0.66 | ↓ |
| 41 | ESI(+) | Gamma-Linolenic acid | 279.232 | 656.456 | (M+H)+ | KEGG | - | - | - |  | 1.55 | 0.62 | ↓ |
| 42 | ESI(-) | 6''-O-Carbamoylkanamycin A | 548.232 | 451.017 | (M+Na-2H)- | KEGG | 5.09 | 3.09 | ↑** |  | 4.07 | 2.74 | ↑* |
| 43 | ESI(-) | 1D-myo-Inositol 1,3,4,5-tetrakisphosphate | 536.871 | 47.830 | (M+K-2H)- | KEGG | - | - | - |  | 1.92 | 0.45 | ↓ |
| 44 | ESI(-) | L-Olivosyl-oleandolide | 515.287 | 390.278 | (M-H)- | KEGG | - | - | - |  | 1.48 | 1.62 | ↑ |
| 45 | ESI(-) | Taurocholate | 514.284 | 391.766 | (M-H)- | KEGG | 4.31 | 3.00 | ↑* |  | 4.10 | 2.92 | ↑* |
| 46 | ESI(-) | Antibiotic G-418 | 512.295 | 402.461 | (M+NH4-2H)- | KEGG | - | - | - |  | 1.48 | 1.88 | ↑ |
| 47 | ESI(-) | L-Oleandrosyl-oleandolide | 511.291 | 402.722 | (M-H2O-H)- | KEGG | 2.44 | 1.94 | ↑ |  | 2.99 | 2.54 | ↑ |
| 48 | ESI(-) | Mupirocin | 499.292 | 441.674 | (M-H)- | KEGG | 4.83 | 3.37 | ↑** |  | 4.77 | 4.39 | ↑ |
| 49 | ESI(-) | Taurodeoxycholate | 498.289 | 442.703 | (M-H)- | KEGG | 7.52 | 5.80 | ↑** |  | 6.92 | 6.72 | ↑* |
| 50 | ESI(-) | Cholesterol sulfate | 465.305 | 406.352 | (M-H)- | KEGG | 6.59 | 4.86 | ↑* |  | 6.69 | 5.29 | ↑* |
| 51 | ESI(-) | Glycocholate | 464.302 | 406.214 | (M-H)- | KEGG | 7.56 | 5.93 | ↑* |  | 7.65 | 6.43 | ↑* |
| 52 | ESI(-) | 3,5-Diiodo-4-hydroxyphenylpyruvate | 450.844 | 45.021 | (M+F)- | KEGG | 3.48 | 1.98 | ↑ |  | - | - | - |
| 53 | ESI(-) | Glycodeoxycholate | 448.307 | 451.439 | (M-H)- | KEGG | 7.31 | 4.90 | ↑** |  | 6.34 | 4.76 | ↑* |
| 54 | ESI(-) | Digalacturonate | 391.050 | 75.469 | (M+Na-2H)- | KEGG | 1.48 | 0.60 | ↓ |  | - | - | - |
| 55 | ESI(-) | Norlinolenic acid | 280.237 | 691.326 | (M+NH4-2H)- | KEGG | - | - | - |  | 1.90 | 0.63 | ↓* |
| 56 | ESI(-) | 1,1-Dichloroethylene epoxide | 132.923 | 814.982 | (M+Na-2H)- | KEGG | - | - | - |  | 1.18 | 0.66 | ↓ |
| 57 | ESI(-) | Sulfate | 96.960 | 68.737 | (M-H)- | KEGG | 1.41 | 0.65 | ↓ |  | - | - | - |
| 58 | ESI(-) | Linoleic acid | 559.473 | 691.034 | (2M-H)- | HMDB | - | - | - |  | 2.17 | 0.61 | ↓ |
| 59 | ESI(-) | Irbesartan | 427.216 | 449.210 | (M-H)- | HMDB | - | - | - |  | 1.19 | 1.8 | ↑ |
| 60 | ESI(-) | Deoxycholic acid | 391.285 | 522.588 | (M-H)- | HMDB | 1.19 | 1.62 | ↑ |  | 4.26 | 2.76 | ↑* |
| 61 | ESI(-) | Oleic acid | 281.249 | 736.704 | (M-H)- | HMDB | - | - | - |  | 1.97 | 0.62 | ↓* |
| 62 | ESI(-) | Bovinic acid | 279.233 | 691.362 | (M-H)- | HMDB | - | - | - |  | 1.90 | 0.62 | ↓* |
| 63 | ESI(-) | Alpha-Linolenic acid | 277.218 | 657.186 | (M-H)- | HMDB | - | - | - |  | 1.97 | 0.59 | ↓* |
| 64 | ESI(-) | L-Phenylalanine | 164.072 | 185.038 | (M-H)- | HMDB | - | - | - |  | 2.12 | 1.77 | ↑ |

“-”: VIP or FC does not meet the filtering conditions, ↑: up-regulated, ↓: down-regulated, *: P < 0.05, **P < 0.01.

**Supplementary Table S3.** 2 mg BID_day 7: The identification of differential metabolites based on untargeted metabolomics.

| NO | Ion mode | Identification | mz | rt | Adduct | Database | d7_1h vs d7_0h | | |  | d7_2h vs d7_0h | | |
| --- | --- | --- | --- | --- | --- | --- | --- | --- | --- | --- | --- | --- | --- |
|  |  |  |  |  |  |  | VIP | FC | Trend |  | VIP | FC | Trend |
| 1 | ESI(+) | Yessotoxin | 1160.534 | 383.804 | (M+NH4)+ | KEGG | 2.14 | 0.25 | ↓ |  | 2.05 | 0.25 | ↓ |
| 2 | ESI(+) | Chlorobactene | 1087.812 | 376.100 | (2M+Na)+ | KEGG | 1.95 | 0.61 | ↓ |  | - | - | - |
| 3 | ESI(+) | Petromyzonol | 811.603 | 656.376 | (2M+Na)+ | KEGG | 1.26 | 0.57 | ↓ |  | - | - | - |
| 4 | ESI(+) | Plastoquinone-9 | 787.604 | 536.623 | (M+K)+ | KEGG | 1.31 | 1.54 | ↑* |  | - | - | - |
| 5 | ESI(+) | APC | 601.265 | 328.231 | (M+H-H2O)+ | KEGG | - | - | - |  | 6.63 | 2.40 | ↑ |
| 6 | ESI(+) | L-Urobilinogen | 597.363 | 460.602 | (M+H)+ | KEGG | 1.80 | 1.78 | ↑ |  | - | - | - |
| 7 | ESI(+) | Rocuronium | 596.352 | 460.205 | (M-2H+3Na)+ | KEGG | - | - | - |  | 2.74 | 2.21 | ↑ |
| 8 | ESI(+) | Graphinone | 593.333 | 462.508 | (2M+H)+ | KEGG | 2.35 | 1.83 | ↑* |  | 5.77 | 4.39 | ↑* |
| 9 | ESI(+) | D-Urobilinogen | 591.317 | 346.610 | (M+H)+ | KEGG | - | - | - |  | 3.84 | 3.00 | ↑ |
| 10 | ESI(+) | Podocarpic acid | 587.276 | 388.127 | (2M+K)+ | KEGG | - | - | - |  | 1.79 | 0.65 | ↓** |
| 11 | ESI(+) | alpha-Tocopherol acetate | 495.379 | 450.388 | (M+Na)+ | KEGG | 8.46 | 6.47 | ↑** |  | 6.28 | 3.93 | ↑ |
| 12 | ESI(+) | Molybdoenzyme molybdenum cofactor | 487.863 | 47.150 | (M+H-2H2O)+ | KEGG | - | - | - |  | 2.56 | 7.98 | ↑ |
| 13 | ESI(+) | Cholic acid | 450.321 | 451.330 | (M+CH3CN+H)+ | KEGG | 8.85 | 6.91 | ↑** |  | 6.16 | 3.35 | ↑* |
| 14 | ESI(+) | 3alpha,7alpha-Dihydroxy-12-oxo-5beta-cholanate | 448.306 | 432.057 | (M+CH3CN+H)+ | KEGG | 4.49 | 3.89 | ↑* |  | 3.03 | 2.33 | ↑ |
| 15 | ESI(+) | 3-Dehydro-2-deoxyecdysone | 429.300 | 503.538 | (M+H-H2O)+ | KEGG | - | - | - |  | 1.63 | 1.51 | ↑* |
| 16 | ESI(+) | Tetrahydrocorticosterone | 368.280 | 425.333 | (M+NH4)+ | KEGG | - | - | - |  | 2.36 | 0.57 | ↓* |
| 17 | ESI(+) | 3-Ketosphingosine | 364.220 | 378.593 | (M-2H+3Na)+ | KEGG | 2.47 | 0.56 | ↓** |  | 2.29 | 0.62 | ↓* |
| 18 | ESI(+) | Phytanate | 359.316 | 763.026 | (M+HCOO+2H)+ | KEGG | 1.09 | 0.63 | ↓ |  | 2.50 | 0.66 | ↓ |
| 19 | ESI(+) | Propofol | 357.279 | 520.791 | (2M+H)+ | KEGG | 2.66 | 1.72 | ↑ |  | 5.95 | 3.72 | ↑ |
| 20 | ESI(+) | (15S)-15-Hydroxy-5,8,11-cis-13-trans-eicosatetraenoate | 338.269 | 691.359 | (M+NH4)+ | KEGG | - | - | - |  | 3.49 | 0.46 | ↓* |
| 21 | ESI(+) | Erucic acid | 321.316 | 649.828 | (M+H-H2O)+ | KEGG | 1.80 | 0.65 | ↓ |  | 3.44 | 0.49 | ↓* |
| 22 | ESI(+) | Sannamycin B | 315.236 | 379.900 | (M+H-H2O)+ | KEGG | - | - | - |  | 2.12 | 0.63 | ↓ |
| 23 | ESI(+) | 10-Deoxymethynolide | 314.233 | 379.904 | (M+NH4)+ | KEGG | - | - | - |  | 1.90 | 0.64 | ↓ |
| 24 | ESI(+) | Naringenin | 314.102 | 64.420 | (M+CH3CN+H)+ | KEGG | - | - | - |  | 2.08 | 1.51 | ↑* |
| 25 | ESI(+) | (10S)-Juvenile hormone III acid diol | 288.217 | 359.979 | (M+NH4)+ | KEGG | 1.73 | 0.64 | ↓** |  | 2.91 | 0.48 | ↓** |
| 26 | ESI(+) | (9Z)-Octadecenoic acid | 283.264 | 737.497 | (M+H)+ | KEGG | - | - | - |  | 3.63 | 0.43 | ↓* |
| 27 | ESI(+) | Gamma-Linolenic acid | 279.232 | 656.456 | (M+H)+ | KEGG | - | - | - |  | 2.92 | 0.48 | ↓* |
| 28 | ESI(+) | O-Butanoylcarnitine | 232.155 | 230.193 | (M+H)+ | KEGG | 1.24 | 0.66 | ↓ |  | - | - | - |
| 29 | ESI(+) | Cyclohexylamine | 166.058 | 95.612 | (M-2H+3Na)+ | KEGG | - | - | - |  | 2.13 | 6.86 | ↑ |
| 30 | ESI(+) | Dichloromethane | 125.986 | 814.938 | (M+CH3CN+H)+ | KEGG | 2.36 | 0.64 | ↓** |  | - | - | - |
| 31 | ESI(+) | Cortisol | 363.217 | 378.637 | (M+H)+ | HMDB | 2.33 | 0.57 | ↓** |  | 2.10 | 0.65 | ↓* |
| 32 | ESI(+) | Piperine | 286.144 | 475.909 | (M+H)+ | HMDB | - | - | - |  | 2.86 | 1.61 | ↑ |
| 33 | ESI(-) | Ethylmorphine | 625.344 | 398.827 | (2M-H)- | KEGG | - | - | - |  | 2.41 | 1.72 | ↑* |
| 34 | ESI(-) | L-Urobilin | 593.334 | 460.232 | (M-H)- | KEGG | - | - | - |  | 2.96 | 2.05 | ↑ |
| 35 | ESI(-) | I-Urobilinogen | 591.319 | 462.997 | (M-H)- | KEGG | - | - | - |  | 2.66 | 2.37 | ↑ |
| 36 | ESI(-) | 6''-O-Carbamoylkanamycin A | 548.232 | 451.017 | (M+Na-2H)- | KEGG | 4.19 | 2.93 | ↑** |  | 2.49 | 1.71 | ↑ |
| 37 | ESI(-) | L-Olivosyl-oleandolide | 515.287 | 390.278 | (M-H)- | KEGG | 3.67 | 3.36 | ↑* |  | 1.55 | 1.62 | ↑ |
| 38 | ESI(-) | Taurocholate | 514.284 | 391.766 | (M-H)- | KEGG | 5.61 | 5.18 | ↑** |  | 3.78 | 2.55 | ↑* |
| 39 | ESI(-) | Mupirocin | 499.292 | 441.674 | (M-H)- | KEGG | 6.11 | 5.32 | ↑** |  | 3.49 | 2.30 | ↑ |
| 40 | ESI(-) | Taurodeoxycholate | 498.289 | 442.703 | (M-H)- | KEGG | 7.45 | 5.97 | ↑** |  | 4.83 | 2.65 | ↑ |
| 41 | ESI(-) | Cholesterol sulfate | 465.305 | 406.352 | (M-H)- | KEGG | 6.64 | 5.67 | ↑** |  | 4.51 | 2.90 | ↑* |
| 42 | ESI(-) | Glycocholate | 464.302 | 406.214 | (M-H)- | KEGG | 9.36 | 7.16 | ↑** |  | 7.62 | 3.71 | ↑* |
| 43 | ESI(-) | 3,5-Diiodo-4-hydroxyphenylpyruvate | 450.844 | 45.021 | (M+F)- | KEGG | - | - | - |  | 6.54 | 2.41 | ↑ |
| 44 | ESI(-) | Glycodeoxycholate | 448.307 | 451.439 | (M-H)- | KEGG | 6.77 | 3.98 | ↑** |  | 5.15 | 2.27 | ↑* |
| 45 | ESI(-) | Paliperidone | 425.201 | 378.787 | (M-H)- | KEGG | 2.11 | 0.55 | ↓** |  | - | - | - |
| 46 | ESI(-) | Cascarillin | 407.207 | 379.043 | (M-H)- | KEGG | 2.10 | 0.55 | ↓** |  | - | - | - |
| 47 | ESI(-) | Norlinolenic acid | 280.237 | 691.326 | (M+NH4-2H)- | KEGG | - | - | - |  | 2.50 | 0.49 | ↓* |
| 48 | ESI(-) | Umbelliferone | 178.051 | 297.040 | (M+NH4-2H)- | KEGG | - | - | - |  | 1.97 | 1.62 | ↑ |
| 49 | ESI(-) | Linoleic acid | 559.473 | 691.034 | (2M-H)- | HMDB | - | - | - |  | 2.91 | 0.44 | ↓ |
| 50 | ESI(-) | Deoxycholic acid | 391.285 | 522.588 | (M-H)- | HMDB | 1.54 | 1.80 | ↑ |  | 4.69 | 3.60 | ↑* |
| 51 | ESI(-) | Arachidonic acid | 303.233 | 682.363 | (M-H)- | HMDB | - | - | - |  | 1.91 | 0.62 | ↓** |
| 52 | ESI(-) | Oleic acid | 281.249 | 736.704 | (M-H)- | HMDB | - | - | - |  | 2.21 | 0.54 | ↓* |
| 53 | ESI(-) | Bovinic acid | 279.233 | 691.362 | (M-H)- | HMDB | - | - | - |  | 2.33 | 0.52 | ↓* |
| 54 | ESI(-) | Alpha-Linolenic acid | 277.218 | 657.186 | (M-H)- | HMDB | - | - | - |  | 2.77 | 0.46 | ↓* |
| 55 | ESI(-) | Palmitic acid | 255.233 | 726.302 | (M-H)- | HMDB | - | - | - |  | 1.81 | 0.61 | ↓* |
| 56 | ESI(-) | Palmitoleic acid | 253.218 | 678.007 | (M-H)- | HMDB | - | - | - |  | 1.57 | 0.64 | ↓ |
| 57 | ESI(-) | Indoxyl sulfate | 212.003 | 292.730 | (M-H)- | HMDB | - | - | - |  | 1.69 | 1.73 | ↑* |

“-”: VIP or FC does not meet the filtering conditions, ↑: up-regulated, ↓: down-regulated, *: P < 0.05, **P < 0.01.

**Supplementary Table S4.** Placebo_Day 1: The identification of differential metabolites based on untargeted metabolomics.

| NO | Ion mode | Identification | mz | rt | Adduct | Database | d1_1h vs d1_0h | | |  | d1_2h vs d1_0h | | |
| --- | --- | --- | --- | --- | --- | --- | --- | --- | --- | --- | --- | --- | --- |
|  |  |  |  |  |  |  | VIP | FC | Trend |  | VIP | FC | Trend |
| 1 | ESI(+) | 3,5-Dihydroxyphenylacetyl-CoA | 981.178 | 65.588 | (M+CH3CN+Na)+ | KEGG | - | - | - |  | 1.85 | 0.64 | ↓ |
| 2 | ESI(+) | Petromyzonol | 811.604 | 734.065 | (2M+Na)+ | KEGG | 2.81 | 0.62 | ↓ |  | - | - | - |
| 3 | ESI(+) | Plastoquinone-9 | 787.604 | 510.075 | (M+K)+ | KEGG | - | - | - |  | 1.78 | 0.62 | ↓ |
| 4 | ESI(+) | Ambenonium | 651.145 | 434.775 | (M-2H+3K)+ | KEGG | - | - | - |  | 1.06 | 0.63 | ↓ |
| 5 | ESI(+) | N-Feruloyltyramine | 649.250 | 445.597 | (2M+Na)+ | KEGG | - | - | - |  | 1.14 | 2.05 | ↑ |
| 6 | ESI(+) | Clobenpropit | 617.179 | 434.546 | (2M+H)+ | KEGG | - | - | - |  | 1.62 | 0.61 | ↓ |
| 7 | ESI(+) | 15-Demethoxy-epsilon-rhodomycin | 616.176 | 434.554 | (M-H+2Na)+ | KEGG | - | - | - |  | 1.67 | 0.61 | ↓ |
| 8 | ESI(+) | alpha-Tocopherol acetate | 495.379 | 450.388 | (M+Na)+ | KEGG | 3.06 | 2.96 | ↑* |  | - | - | - |
| 9 | ESI(+) | Cholic acid | 450.321 | 451.330 | (M+CH3CN+H)+ | KEGG | 3.39 | 2.84 | ↑** |  | - | - | - |
| 10 | ESI(+) | Ajmaline | 441.074 | 57.515 | (M-2H+3K)+ | KEGG | 2.06 | 1.74 | ↑** |  | 2.88 | 2.18 | ↑** |
| 11 | ESI(+) | Fortimicin FU-10 | 385.121 | 56.810 | (M-H+2Na)+ | KEGG | 1.90 | 1.76 | ↑** |  | 2.53 | 1.94 | ↑** |
| 12 | ESI(+) | (S)-Canadine | 384.119 | 56.727 | (M-H+2Na)+ | KEGG | 2.15 | 1.75 | ↑** |  | 2.35 | 1.86 | ↑** |
| 13 | ESI(+) | Propofol | 357.279 | 520.791 | (2M+H)+ | KEGG | 2.59 | 1.62 | ↑ |  | - | - | - |
| 14 | ESI(+) | (15S)-15-Hydroxy-5,8,11-cis-13-trans-eicosatetraenoate | 338.269 | 691.359 | (M+NH4)+ | KEGG | - | - | - |  | 1.88 | 0.60 | ↓ |
| 15 | ESI(+) | Tridihexethyl | 336.327 | 769.197 | (M+NH4)+ | KEGG | 1.86 | 0.61 | ↓ |  | 3.06 | 0.39 | ↓ |
| 16 | ESI(+) | 1'-Acetoxyeugenol acetate | 331.053 | 60.960 | (M-2H+3Na)+ | KEGG | 1.87 | 1.52 | ↑ |  | - | - | - |
| 17 | ESI(+) | Naphazoline hydrochloride | 323.011 | 58.961 | (M-H+2K)+ | KEGG | 1.91 | 1.56 | ↑* |  | - | - | - |
| 18 | ESI(+) | Erucic acid | 321.316 | 649.828 | (M+H-H2O)+ | KEGG | 3.78 | 0.42 | ↓** |  | 3.75 | 0.42 | ↓** |
| 19 | ESI(+) | Methamidophos | 320.967 | 57.159 | (2M+K)+ | KEGG | 1.60 | 1.54 | ↑** |  | 1.76 | 1.61 | ↑** |
| 20 | ESI(+) | Octadecanal | 310.311 | 753.415 | (M+CH3CN+H)+ | KEGG | - | - | - |  | 1.13 | 0.56 | ↓ |
| 21 | ESI(+) | (9Z)-Octadecenoic acid | 283.264 | 737.497 | (M+H)+ | KEGG | - | - | - |  | 4.36 | 0.42 | ↓** |
| 22 | ESI(+) | Gamma-Linolenic acid | 279.232 | 656.456 | (M+H)+ | KEGG | - | - | - |  | 3.76 | 0.45 | ↓* |
| 23 | ESI(+) | Allantoin | 205.057 | 56.745 | (M+HCOO+2H)+ | KEGG | 1.59 | 1.54 | ↑** |  | 2.01 | 1.69 | ↑** |
| 24 | ESI(+) | 4-Guanidinobutanamide | 167.089 | 182.243 | (M+Na)+ | KEGG | 1.83 | 1.58 | ↑ |  | - | - | - |
| 25 | ESI(+) | L-Phenylalanine | 166.086 | 185.032 | (M+H)+ | KEGG | 1.78 | 1.59 | ↑ |  | 3.40 | 1.88 | ↑** |
| 26 | ESI(+) | Piperine | 286.144 | 475.909 | (M+H)+ | HMDB | 3.47 | 1.83 | ↑** |  | 3.60 | 1.90 | ↑** |
| 27 | ESI(+) | Tyramine | 120.081 | 185.213 | (M+H-H2O)+ | HMDB | 1.81 | 1.53 | ↑ |  | 2.50 | 1.53 | ↑ |
| 28 | ESI(-) | Siroheme | 935.202 | 67.731 | (M+F)- | KEGG | 2.26 | 0.62 | ↓ |  | 2.54 | 0.63 | ↓* |
| 29 | ESI(-) | Nickel-sirohydrochlorin a,c-diamide | 897.248 | 68.758 | (M-H2O-H)- | KEGG | 1.66 | 0.64 | ↓ |  | - | - | - |
| 30 | ESI(-) | 1D-myo-Inositol 1,3,4,5-tetrakisphosphate | 536.871 | 47.830 | (M+K-2H)- | KEGG | 2.70 | 0.59 | ↓ |  | - | - | - |
| 31 | ESI(-) | Glycochenodeoxycholate 7-sulfate | 528.263 | 422.020 | (M-H)- | KEGG | 1.84 | 1.56 | ↑* |  | - | - | - |
| 32 | ESI(-) | Taurodeoxycholate | 498.289 | 442.703 | (M-H)- | KEGG | 4.93 | 2.91 | ↑** |  | - | - | - |
| 33 | ESI(-) | Cholesterol sulfate | 465.305 | 406.352 | (M-H)- | KEGG | 3.86 | 1.98 | ↑* |  | - | - | - |
| 34 | ESI(-) | Glycocholate | 464.302 | 406.214 | (M-H)- | KEGG | 4.46 | 2.85 | ↑* |  | - | - | - |
| 35 | ESI(-) | 3,5-Diiodo-4-hydroxyphenylpyruvate | 450.844 | 45.021 | (M+F)- | KEGG | - | - | - |  | 1.88 | 1.74 | ↑ |
| 36 | ESI(-) | Glycodeoxycholate | 448.307 | 451.439 | (M-H)- | KEGG | 4.19 | 2.84 | ↑* |  | - | - | - |
| 37 | ESI(-) | Digalacturonate | 391.050 | 75.469 | (M+Na-2H)- | KEGG | - | - | - |  | 1.85 | 0.66 | ↓ |
| 38 | ESI(-) | Docosahexaenoic acid | 327.233 | 671.838 | (M-H)- | KEGG | - | - | - |  | 3.64 | 0.51 | ↓** |
| 39 | ESI(-) | Norlinolenic acid | 280.237 | 691.326 | (M+NH4-2H)- | KEGG | 2.50 | 0.63 | ↓** |  | 6.03 | 0.33 | ↓** |
| 40 | ESI(-) | Taurocyamine | 188.010 | 322.135 | (M+Na-2H)- | KEGG | - | - | - |  | 3.79 | 1.53 | ↑ |
| 41 | ESI(-) | D-Glucose | 179.056 | 56.424 | (M-H)- | KEGG | 2.12 | 1.54 | ↑** |  | 2.77 | 1.64 | ↑** |
| 42 | ESI(-) | Linoleic acid | 559.473 | 691.034 | (2M-H)- | HMDB | 2.84 | 0.65 | ↓** |  | 3.33 | 0.60 | ↓** |
| 43 | ESI(-) | Arachidonic acid | 303.233 | 682.363 | (M-H)- | HMDB | - | - | - |  | 4.05 | 0.47 | ↓** |
| 44 | ESI(-) | Oleic acid | 281.249 | 736.704 | (M-H)- | HMDB | 2.38 | 0.65 | ↓** |  | 5.94 | 0.33 | ↓** |
| 45 | ESI(-) | Bovinic acid | 279.233 | 691.362 | (M-H)- | HMDB | - | - | - |  | 5.57 | 0.36 | ↓** |
| 46 | ESI(-) | Alpha-Linolenic acid | 277.218 | 657.186 | (M-H)- | HMDB | 2.50 | 0.62 | ↓** |  | 6.41 | 0.32 | ↓** |
| 47 | ESI(-) | Palmitic acid | 255.233 | 726.302 | (M-H)- | HMDB | 2.42 | 0.63 | ↓** |  | 4.59 | 0.42 | ↓** |
| 48 | ESI(-) | Palmitoleic acid | 253.218 | 678.007 | (M-H)- | HMDB | 2.77 | 0.56 | ↓* |  | 3.66 | 0.52 | ↓* |

“-”: VIP or FC does not meet the filtering conditions, ↑: up-regulated, ↓: down-regulated, *: P < 0.05, **P < 0.01.

**Supplementary Table S5.** Placebo_Day 7: The identification of differential metabolites based on untargeted metabolomics.

| NO | Ion mode | Identification | mz | rt | Adduct | Database | d7_1h vs d7_0h | | |  | d7_2h vs d7_0h | | |
| --- | --- | --- | --- | --- | --- | --- | --- | --- | --- | --- | --- | --- | --- |
|  |  |  |  |  |  |  | VIP | FC | Trend |  | VIP | FC | Trend |
| 1 | ESI(+) | Chenodeoxycholoyl-CoA | 1142.393 | 385.523 | (M+H)+ | KEGG | 2.16 | 0.49 | ↓ |  | 2.17 | 0.45 | ↓ |
| 2 | ESI(+) | Gambiriin C | 1142.327 | 385.568 | (2M+NH4)+ | KEGG | 2.46 | 0.39 | ↓ |  | 1.94 | 0.40 | ↓ |
| 3 | ESI(+) | trans-2-Enoyl-OPC8-CoA | 1105.314 | 373.426 | (M+CH3CN+Na)+ | KEGG | - | - | - |  | 1.80 | 1.51 | ↑* |
| 4 | ESI(+) | beta-Carotene | 1073.928 | 374.528 | (2M+H)+ | KEGG | 3.17 | 0.37 | ↓ |  | 2.18 | 0.42 | ↓ |
| 5 | ESI(+) | Hyperforin | 1073.802 | 374.435 | (2M+H)+ | KEGG | 2.37 | 0.50 | ↓ |  | 1.71 | 0.57 | ↓ |
| 6 | ESI(+) | Mascaroside | 1071.432 | 385.136 | (2M+Na)+ | KEGG | 2.18 | 0.49 | ↓ |  | 1.83 | 0.48 | ↓ |
| 7 | ESI(+) | Chikusetsusaponin V | 1071.369 | 385.470 | (M-2H+3K)+ | KEGG | 2.09 | 0.45 | ↓ |  | 1.76 | 0.48 | ↓ |
| 8 | ESI(+) | UDP-L-Ara4N | 1071.119 | 385.480 | (2M+H)+ | KEGG | 2.23 | 0.48 | ↓ |  | 1.76 | 0.53 | ↓ |
| 9 | ESI(+) | Maltohexaose | 1008.348 | 385.416 | (M+NH4)+ | KEGG | 2.24 | 0.40 | ↓ |  | 1.73 | 0.43 | ↓ |
| 10 | ESI(+) | 3-Oxododecanoyl-CoA | 1008.230 | 385.419 | (M-H+2Na)+ | KEGG | 2.17 | 0.49 | ↓ |  | 1.85 | 0.48 | ↓ |
| 11 | ESI(+) | Feruloyl-diketide-CoA | 1008.171 | 385.469 | (M+Na)+ | KEGG | 2.42 | 0.45 | ↓ |  | 2.10 | 0.45 | ↓ |
| 12 | ESI(+) | Tylosin | 954.492 | 374.377 | (M+K)+ | KEGG | 2.35 | 0.50 | ↓ |  | 1.90 | 0.52 | ↓ |
| 13 | ESI(+) | Nonanoyl-CoA | 954.269 | 374.386 | (M+HCOO+2H)+ | KEGG | 2.29 | 0.52 | ↓ |  | 1.84 | 0.53 | ↓ |
| 14 | ESI(+) | 2-Succinylbenzoyl-CoA | 954.158 | 374.356 | (M+H-H2O)+ | KEGG | 2.34 | 0.49 | ↓ |  | 2.00 | 0.49 | ↓ |
| 15 | ESI(+) | Salmeterol | 848.552 | 810.069 | (2M+NH4)+ | KEGG | 2.36 | 1.56 | ↑ |  | - | - | - |
| 16 | ESI(+) | 4alpha-Methyl-5alpha-cholest-7-en-3-one | 835.665 | 808.138 | (2M+K)+ | KEGG | 3.28 | 0.53 | ↓* |  | 1.83 | 0.63 | ↓ |
| 17 | ESI(+) | Petromyzonol | 811.603 | 656.376 | (2M+Na)+ | KEGG | - | - | - |  | 1.64 | 1.51 | ↑ |
| 18 | ESI(+) | Plastoquinone-9 | 787.604 | 787.898 | (M+K)+ | KEGG | - | - | - |  | 2.13 | 1.82 | ↑ |
| 19 | ESI(+) | Pregnanolone | 659.513 | 668.248 | (2M+Na)+ | KEGG | 1.24 | 0.43 | ↓ |  | - | - | - |
| 20 | ESI(+) | (15S)-15-Hydroxy-5,8,11-cis-13-trans-eicosatetraenoate | 658.510 | 668.592 | (2M+NH4)+ | KEGG | 1.25 | 0.52 | ↓ |  | - | - | - |
| 21 | ESI(+) | Clobenpropit | 617.179 | 434.546 | (2M+H)+ | KEGG | 1.49 | 1.93 | ↑ |  | - | - | - |
| 22 | ESI(+) | 15-Demethoxy-epsilon-rhodomycin | 616.176 | 434.554 | (M-H+2Na)+ | KEGG | 1.49 | 1.93 | ↑ |  | - | - | - |
| 23 | ESI(+) | 3,4-Dihydroanhydrorhodovibrin | 615.487 | 670.469 | (M+HCOO+2H)+ | KEGG | 1.03 | 0.58 | ↓ |  | - | - | - |
| 24 | ESI(+) | Pancuronium | 614.484 | 670.647 | (M+CH3CN+H)+ | KEGG | 1.19 | 0.62 | ↓ |  | - | - | - |
| 25 | ESI(+) | APC | 601.265 | 328.231 | (M+H-H2O)+ | KEGG | 7.08 | 2.25 | ↑ |  | 1.84 | 1.53 | ↑ |
| 26 | ESI(+) | Quercetin 3-O-glucoside | 511.108 | 69.000 | (M+HCOO+2H)+ | KEGG | 2.68 | 1.52 | ↑** |  | 3.93 | 2.06 | ↑** |
| 27 | ESI(+) | Cholic acid | 450.321 | 451.330 | (M+CH3CN+H)+ | KEGG | 2.94 | 1.55 | ↑ |  | 2.18 | 1.86 | ↑ |
| 28 | ESI(+) | Ajmaline | 441.074 | 57.515 | (M-2H+3K)+ | KEGG | 3.16 | 1.96 | ↑** |  | 3.21 | 2.11 | ↑** |
| 29 | ESI(+) | Teleocidin B-1 | 434.317 | 450.150 | (M+H-H2O)+ | KEGG | 1.43 | 0.25 | ↓ |  | - | - | - |
| 30 | ESI(+) | Fortimicin FU-10 | 385.121 | 56.810 | (M-H+2Na)+ | KEGG | 2.23 | 1.65 | ↑** |  | 2.50 | 1.78 | ↑** |
| 31 | ESI(+) | (S)-Canadine | 384.119 | 56.727 | (M-H+2Na)+ | KEGG | 2.25 | 1.65 | ↑** |  | 2.23 | 1.69 | ↑** |
| 32 | ESI(+) | Tetrahydrocorticosterone | 368.280 | 425.333 | (M+NH4)+ | KEGG | - | - | - |  | 2.89 | 0.49 | ↓ |
| 33 | ESI(+) | Propofol | 357.279 | 520.791 | (2M+H)+ | KEGG | - | - | - |  | 2.13 | 0.66 | ↓ |
| 34 | ESI(+) | Tridihexethyl | 319.285 | 652.168 | (M+H)+ | KEGG | - | - | - |  | 2.30 | 1.65 | ↑* |
| 35 | ESI(+) | (10S)-Juvenile hormone III acid diol | 288.217 | 359.979 | (M+NH4)+ | KEGG | - | - | - |  | 2.42 | 0.56 | ↓* |
| 36 | ESI(+) | (9Z)-Octadecenoic acid | 283.264 | 737.497 | (M+H)+ | KEGG | - | - | - |  | 5.01 | 0.40 | ↓** |
| 37 | ESI(+) | Gamma-Linolenic acid | 279.232 | 656.456 | (M+H)+ | KEGG | - | - | - |  | 3.82 | 0.49 | ↓* |
| 38 | ESI(+) | Tromethamine | 260.186 | 317.129 | (2M+NH4)+ | KEGG | - | - | - |  | 2.50 | 0.59 | ↓ |
| 39 | ESI(+) | Allantoin | 205.057 | 56.745 | (M+HCOO+2H)+ | KEGG | 2.10 | 1.55 | ↑** |  | 2.03 | 1.58 | ↑** |
| 40 | ESI(+) | L-Phenylalanine | 166.086 | 185.032 | (M+H)+ | KEGG | - | - | - |  | 2.51 | 1.59 | ↑ |
| 41 | ESI(+) | Dodecanoylcarnitine | 344.280 | 427.023 | (M+H)+ | HMDB | - | - | - |  | 2.01 | 0.64 | ↓ |
| 42 | ESI(-) | Decanoyl-CoA | 958.187 | 65.395 | (M+K-2H)- | KEGG | 4.40 | 1.78 | ↑** |  | - | - | - |
| 43 | ESI(-) | Glycodeoxycholate | 897.623 | 450.564 | (2M-H)- | KEGG | - | - | - |  | 2.83 | 2.93 | ↑ |
| 44 | ESI(-) | S-Glutathionyl-L-cysteine | 851.166 | 60.445 | (2M-H)- | KEGG | - | - | - |  | 2.78 | 1.56 | ↑** |
| 45 | ESI(-) | 2'''-N-Acetyl-6'''-deamino-6'''-hydroxyparomomycin II | 679.263 | 413.723 | (M+Na-2H)- | KEGG | 1.58 | 1.72 | ↑ |  | - | - | - |
| 46 | ESI(-) | 3-Hydroxyethylchlorophyllide a | 631.242 | 436.460 | (M-H)- | KEGG | 1.12 | 1.68 | ↑ |  | - | - | - |
| 47 | ESI(-) | 6''-O-Carbamoylkanamycin A | 548.232 | 451.017 | (M+Na-2H)- | KEGG | - | - | - |  | 3.93 | 1.71 | ↑ |
| 48 | ESI(-) | 1D-myo-Inositol 1,3,4,5-tetrakisphosphate | 536.871 | 47.830 | (M+K-2H)- | KEGG | 3.31 | 1.90 | ↑ |  | 2.59 | 1.85 | ↑ |
| 49 | ESI(-) | Glycochenodeoxycholate 7-sulfate | 528.263 | 422.020 | (M-H)- | KEGG | 5.11 | 1.69 | ↑ |  | - | - | - |
| 50 | ESI(-) | Mupirocin | 499.292 | 441.674 | (M-H)- | KEGG | - | - | - |  | 1.51 | 1.82 | ↑ |
| 51 | ESI(-) | Taurodeoxycholate | 498.289 | 442.703 | (M-H)- | KEGG | - | - | - |  | 2.58 | 2.47 | ↑ |
| 52 | ESI(-) | Glycocholate | 464.302 | 406.214 | (M-H)- | KEGG | 4.82 | 1.62 | ↑ |  | - | - | - |
| 53 | ESI(-) | Prometon | 449.310 | 451.302 | (2M-H)- | KEGG | 3.74 | 1.56 | ↑ |  | - | - | - |
| 54 | ESI(-) | Paliperidone | 425.201 | 378.787 | (M-H)- | KEGG | - | - | - |  | 2.95 | 0.66 | ↓** |
| 55 | ESI(-) | Cascarillin | 407.207 | 379.043 | (M-H)- | KEGG | - | - | - |  | 3.13 | 0.64 | ↓** |
| 56 | ESI(-) | Chenodeoxycholate | 391.285 | 657.364 | (M-H)- | KEGG | 1.17 | 1.77 | ↑ |  | - | - | - |
| 57 | ESI(-) | Taxa-4(20),11(12)-dien-5alpha-yl acetate | 329.249 | 693.228 | (M-H)- | KEGG | 3.63 | 0.55 | ↓* |  | 2.30 | 0.64 | ↓* |
| 58 | ESI(-) | Docosahexaenoic acid | 327.233 | 671.838 | (M-H)- | KEGG | - | - | - |  | 3.85 | 0.56 | ↓** |
| 59 | ESI(-) | (-)-Menthone | 307.265 | 749.046 | (2M-H)- | KEGG | - | - | - |  | 4.03 | 0.51 | ↓** |
| 60 | ESI(-) | Norlinolenic acid | 280.237 | 691.326 | (M+NH4-2H)- | KEGG | 2.63 | 0.65 | ↓* |  | 5.41 | 0.41 | ↓** |
| 61 | ESI(-) | D-Glucose | 179.056 | 56.424 | (M-H)- | KEGG | - | - | - |  | 2.66 | 1.55 | ↑** |
| 62 | ESI(-) | Linoleic acid | 559.473 | 691.034 | (2M-H)- | HMDB | 3.73 | 0.51 | ↓* |  | 3.81 | 0.50 | ↓* |
| 63 | ESI(-) | Arachidonic acid | 303.233 | 682.363 | (M-H)- | HMDB | - | - | - |  | 3.57 | 0.55 | ↓** |
| 64 | ESI(-) | Oleic acid | 281.249 | 736.704 | (M-H)- | HMDB | 2.54 | 0.66 | ↓* |  | 5.52 | 0.40 | ↓** |
| 65 | ESI(-) | Bovinic acid | 279.233 | 691.362 | (M-H)- | HMDB | 2.95 | 0.61 | ↓* |  | 5.30 | 0.41 | ↓** |
| 66 | ESI(-) | Alpha-Linolenic acid | 277.218 | 657.186 | (M-H)- | HMDB | 2.84 | 0.66 | ↓* |  | 5.95 | 0.42 | ↓** |
| 67 | ESI(-) | Palmitic acid | 255.233 | 726.302 | (M-H)- | HMDB | - | - | - |  | 4.54 | 0.46 | ↓** |
| 68 | ESI(-) | Palmitoleic acid | 253.218 | 678.007 | (M-H)- | HMDB | 2.78 | 0.60 | ↓* |  | 5.54 | 0.43 | ↓** |

“-”: VIP or FC does not meet the filtering conditions, ↑: up-regulated, ↓: down-regulated, *: P < 0.05, **P < 0.01.

**Supplementary Table S6.** 0.5 mg BID_Day 1: The identification of differential metabolites based on untargeted metabolomics.

| NO | Ion mode | Identification | mz | rt | Adduct | Database | d1_1h vs d1_0h | | |  | d1_2h vs d1_0h | | |
| --- | --- | --- | --- | --- | --- | --- | --- | --- | --- | --- | --- | --- | --- |
|  |  |  |  |  |  |  | VIP | FC | Trend |  | VIP | FC | Trend |
| 1 | ESI(+) | 1D-myo-Inositol 1,3,4,5,6-pentakisphosphate | 1160.801 | 383.802 | (2M+H)+ | KEGG | - | - | - |  | 3.06 | 0.16 | ↓ |
| 2 | ESI(+) | 21,22-Diprenylpaxilline | 1160.735 | 383.796 | (2M+NH4)+ | KEGG | - | - | - |  | 3.57 | 0.16 | ↓ |
| 3 | ESI(+) | Yessotoxin | 1160.534 | 383.804 | (M+NH4)+ | KEGG | - | - | - |  | 3.19 | 0.13 | ↓ |
| 4 | ESI(+) | GDP-4-dehydro-3,6-dideoxy-alpha-D-mannose | 1160.133 | 384.763 | (2M+NH4)+ | KEGG | - | - | - |  | 2.97 | 0.16 | ↓ |
| 5 | ESI(+) | Ginsenoside Rb1 | 1150.664 | 385.179 | (M+CH3CN+H)+ | KEGG | - | - | - |  | 2.75 | 0.18 | ↓ |
| 6 | ESI(+) | Angiotensin II | 1122.447 | 373.519 | (M-H+2K)+ | KEGG | - | - | - |  | 3.46 | 0.13 | ↓ |
| 7 | ESI(+) | (4Z,7Z,10Z,13Z,16Z,19Z)-Docosahexaenoyl-CoA | 1122.321 | 373.479 | (M-H+2Na)+ | KEGG | - | - | - |  | 3.24 | 0.13 | ↓ |
| 8 | ESI(+) | Maltohexaose | 1105.189 | 373.454 | (M-2H+3K)+ | KEGG | 2.81 | 5.73 | ↑ |  | - | - | - |
| 9 | ESI(+) | Docosenoyl-CoA | 1088.437 | 382.746 | (M+H)+ | KEGG | 3.70 | 5.95 | ↑ |  | - | - | - |
| 10 | ESI(+) | (6Z,9Z,12Z,15Z,18Z,21Z)-Tetracosahexaenoyl-CoA | 1088.376 | 383.832 | (M+H-H2O)+ | KEGG | - | - | - |  | 2.94 | 0.17 | ↓ |
| 11 | ESI(+) | Naphthyl-2-oxomethyl-succinyl-CoA | 1088.127 | 383.808 | (M-2H+3Na)+ | KEGG | - | - | - |  | 3.57 | 0.15 | ↓ |
| 12 | ESI(+) | 5beta-Cyprinolsulfate | 1087.625 | 384.725 | (2M+Na)+ | KEGG | 2.67 | 4.04 | ↑ |  | - | - | - |
| 13 | ESI(+) | 6-Mercaptopurine ribonucleoside triphosphate | 1086.876 | 384.907 | (2M+K)+ | KEGG | 2.62 | 3.19 | ↑ |  | - | - | - |
| 14 | ESI(+) | 2-Hexaprenyl-6-methoxyphenol | 1082.940 | 379.270 | (2M+NH4)+ | KEGG | - | - | - |  | 2.77 | 0.17 | ↓ |
| 15 | ESI(+) | Chlorobactene | 1082.815 | 379.211 | (2M+NH4)+ | KEGG | - | - | - |  | 3.24 | 0.16 | ↓ |
| 16 | ESI(+) | 3-Oxo-OPC8-CoA | 1080.310 | 384.247 | (M+Na)+ | KEGG | 2.78 | 6.11 | ↑ |  | - | - | - |
| 17 | ESI(+) | Icosenoyl-CoA | 1024.414 | 383.813 | (M+H-2H2O)+ | KEGG | - | - | - |  | 3.59 | 0.15 | ↓ |
| 18 | ESI(+) | 3-Oxo-OPC4-CoA | 1024.238 | 383.775 | (M+Na)+ | KEGG | - | - | - |  | 3.12 | 0.16 | ↓ |
| 19 | ESI(+) | Adouetine X | 1023.648 | 384.706 | (2M+Na)+ | KEGG | 2.41 | 3.21 | ↑ |  | - | - | - |
| 20 | ESI(+) | dITP | 1022.943 | 384.666 | (2M+K)+ | KEGG | - | - | - |  | 2.87 | 0.17 | ↓ |
| 21 | ESI(+) | Biotinyl-CoA | 1016.174 | 385.135 | (M+Na)+ | KEGG | 2.96 | 5.18 | ↑ |  | - | - | - |
| 22 | ESI(+) | 20-Hydroxyecdysone | 983.614 | 373.634 | (2M+Na)+ | KEGG | 2.80 | 5.06 | ↑ |  | - | - | - |
| 23 | ESI(+) | Delphinidin-3-(p-coumaroyl)-rutinoside-5-glucoside | 983.281 | 373.468 | (M+CH3CN+Na)+ | KEGG | 2.90 | 5.05 | ↑ |  | - | - | - |
| 24 | ESI(+) | Cyanidin-3-(p-coumaroyl)-rutinoside-5-glucoside | 967.278 | 375.064 | (M+CH3CN+Na)+ | KEGG | 2.96 | 4.98 | ↑ |  | - | - | - |
| 25 | ESI(+) | Decanoyl-CoA | 966.224 | 384.579 | (M-H+2Na)+ | KEGG | - | - | - |  | 3.65 | 0.17 | ↓ |
| 26 | ESI(+) | Flupoxam | 959.110 | 384.952 | (2M+K)+ | KEGG | - | - | - |  | 3.06 | 0.16 | ↓ |
| 27 | ESI(+) | 3-Dehydro-2-deoxyecdysone | 915.582 | 384.529 | (2M+Na)+ | KEGG | 2.49 | 3.76 | ↑ |  | - | - | - |
| 28 | ESI(+) | 1-(5'-Phosphoribosyl)-5-amino-4-(N-succinocarboxamide)-imidazole | 909.157 | 384.910 | (2M+H)+ | KEGG | - | - | - |  | 3.53 | 0.13 | ↓ |
| 29 | ESI(+) | Glucoberteroin | 909.104 | 384.947 | (2M+K)+ | KEGG | - | - | - |  | 2.91 | 0.16 | ↓ |
| 30 | ESI(+) | P1,P4-Bis(5'-xanthosyl) tetraphosphate | 908.947 | 384.933 | (M+K)+ | KEGG | 2.44 | 3.54 | ↑ |  | - | - | - |
| 31 | ESI(+) | DIDS | 908.894 | 384.984 | (2M+H)+ | KEGG | 2.60 | 3.94 | ↑ |  | - | - | - |
| 32 | ESI(+) | Nonacosan-10-one | 883.857 | 371.907 | (2M+K)+ | KEGG | 2.98 | 5.12 | ↑ |  | - | - | - |
| 33 | ESI(+) | 4,4'-Diaponeurosporenic acid | 882.656 | 373.526 | (2M+NH4)+ | KEGG | 2.33 | 5.17 | ↑ |  | - | - | - |
| 34 | ESI(+) | 2,3-Bis-O-(geranylgeranyl)-sn-glycero-1-phospho-L-serine | 804.552 | 808.200 | (M+H)+ | KEGG | 2.63 | 1.56 | ↑ |  | - | - | - |
| 35 | ESI(+) | Butanoyl-CoA | 802.142 | 373.453 | (M+H-2H2O)+ | KEGG | 3.01 | 6.83 | ↑ |  | - | - | - |
| 36 | ESI(+) | Plastoquinone-9 | 787.604 | 758.464 | (M+K)+ | KEGG | 2.34 | 1.55 | ↑ |  | 3.51 | 1.87 | ↑ |
| 37 | ESI(+) | 3-Hydroxyethylbacteriochlorophyllide a | 681.276 | 413.114 | (M+HCOO+2H)+ | KEGG | - | - | - |  | 1.95 | 0.66 | ↓ |
| 38 | ESI(+) | Clobenpropit | 617.179 | 434.546 | (2M+H)+ | KEGG | - | - | - |  | 2.24 | 0.63 | ↓ |
| 39 | ESI(+) | 15-Demethoxy-epsilon-rhodomycin | 616.176 | 434.554 | (M-H+2Na)+ | KEGG | - | - | - |  | 2.09 | 0.65 | ↓ |
| 40 | ESI(+) | APC | 601.265 | 328.231 | (M+H-H2O)+ | KEGG | - | - | - |  | 7.53 | 0.42 | ↓ |
| 41 | ESI(+) | Vecuronium | 522.423 | 718.030 | (M+H-2H2O)+ | KEGG | 1.05 | 0.65 | ↓ |  | 1.20 | 0.65 | ↓ |
| 42 | ESI(+) | Cholic acid | 450.321 | 451.330 | (M+CH3CN+H)+ | KEGG | 3.89 | 2.36 | ↑** |  | - | - | - |
| 43 | ESI(+) | 3alpha,7alpha-Dihydroxy-12-oxo-5beta-cholanate | 448.306 | 432.057 | (M+CH3CN+H)+ | KEGG | 2.05 | 2.21 | ↑ |  | - | - | - |
| 44 | ESI(+) | Ajmaline | 441.074 | 57.515 | (M-2H+3K)+ | KEGG | 2.32 | 1.65 | ↑** |  | 2.66 | 1.74 | ↑** |
| 45 | ESI(+) | Gentamicin C1a | 432.280 | 296.179 | (M+H-H2O)+ | KEGG | 2.09 | 1.67 | ↑* |  | 2.05 | 1.58 | ↑ |
| 46 | ESI(+) | Di(2-ethylhexyl) adipate | 417.319 | 477.068 | (M+HCOO+2H)+ | KEGG | - | - | - |  | 2.51 | 1.51 | ↑ |
| 47 | ESI(+) | Fortimicin FU-10 | 385.121 | 56.810 | (M-H+2Na)+ | KEGG | 2.31 | 1.62 | ↑** |  | 2.43 | 1.62 | ↑** |
| 48 | ESI(+) | (S)-Canadine | 384.119 | 56.727 | (M-H+2Na)+ | KEGG | 2.28 | 1.62 | ↑** |  | 2.63 | 1.67 | ↑** |
| 49 | ESI(+) | Tetrahydrocorticosterone | 368.280 | 425.333 | (M+NH4)+ | KEGG | - | - | - |  | 2.29 | 0.62 | ↓* |
| 50 | ESI(+) | 3-Ketosphingosine | 364.220 | 378.593 | (M-2H+3Na)+ | KEGG | - | - | - |  | 2.36 | 0.60 | ↓* |
| 51 | ESI(+) | Propofol | 357.279 | 520.791 | (2M+H)+ | KEGG | - | - | - |  | 1.64 | 0.62 | ↓* |
| 52 | ESI(+) | Tridihexethyl | 336.327 | 769.197 | (M+NH4)+ | KEGG | - | - | - |  | 2.41 | 0.62 | ↓ |
| 53 | ESI(+) | Erucic acid | 321.316 | 649.828 | (M+H-H2O)+ | KEGG | 5.45 | 0.30 | ↓** |  | 4.10 | 0.56 | ↓* |
| 54 | ESI(+) | Methamidophos | 320.967 | 57.159 | (2M+K)+ | KEGG | - | - | - |  | 2.26 | 1.53 | ↑** |
| 55 | ESI(+) | omega-Cyclohexylundecanoic acid | 315.254 | 696.331 | (M+HCOO+2H)+ | KEGG | - | - | - |  | 1.96 | 0.59 | ↓ |
| 56 | ESI(+) | Octadecanal | 310.311 | 753.415 | (M+CH3CN+H)+ | KEGG | - | - | - |  | 3.53 | 0.51 | ↓ |
| 57 | ESI(+) | (10S)-Juvenile hormone III acid diol | 288.217 | 359.979 | (M+NH4)+ | KEGG | - | - | - |  | 2.07 | 0.64 | ↓* |
| 58 | ESI(+) | (9Z)-Octadecenoic acid | 283.264 | 737.497 | (M+H)+ | KEGG | - | - | - |  | 3.13 | 0.50 | ↓** |
| 59 | ESI(+) | Gamma-Linolenic acid | 279.232 | 656.456 | (M+H)+ | KEGG | - | - | - |  | 4.30 | 0.40 | ↓** |
| 60 | ESI(+) | 4-(L-Alanin-3-yl)-2-hydroxy-cis,cis-muconate 6-semialdehyde | 247.093 | 67.322 | (M+NH4)+ | KEGG | - | - | - |  | 2.76 | 1.72 | ↑ |
| 61 | ESI(+) | Cycasin | 235.093 | 74.453 | (M+H-H2O)+ | KEGG | - | - | - |  | 2.74 | 1.51 | ↑ |
| 62 | ESI(+) | D-Glucose | 225.034 | 55.849 | (M-H+2Na)+ | KEGG | - | - | - |  | 2.16 | 1.51 | ↑** |
| 63 | ESI(+) | Allantoin | 205.057 | 56.745 | (M+HCOO+2H)+ | KEGG | 2.05 | 1.52 | ↑** |  | - | - | - |
| 64 | ESI(+) | Cortisol | 363.217 | 378.637 | (M+H)+ | HMDB | - | - | - |  | 2.42 | 0.60 | ↓* |
| 65 | ESI(+) | Piperine | 286.144 | 475.909 | (M+H)+ | HMDB | 3.57 | 2.15 | ↑ |  | 3.55 | 1.73 | ↑ |
| 66 | ESI(+) | Linoleic acid | 281.248 | 691.187 | (M+H)+ | HMDB | - | - | - |  | 2.78 | 0.58 | ↓* |
| 67 | ESI(-) | S-Glutathionyl-L-cysteine | 851.166 | 60.445 | (2M-H)- | KEGG | - | - | - |  | 3.43 | 1.64 | ↑** |
| 68 | ESI(-) | CoA | 748.097 | 65.987 | (M-H2O-H)- | KEGG | 2.46 | 1.55 | ↑* |  | - | - | - |
| 69 | ESI(-) | 1D-myo-Inositol 1,3,4,5-tetrakisphosphate | 536.871 | 47.830 | (M+K-2H)- | KEGG | - | - | - |  | 3.87 | 0.49 | ↓ |
| 70 | ESI(-) | Glycochenodeoxycholate 7-sulfate | 528.263 | 422.020 | (M-H)- | KEGG | - | - | - |  | 2.59 | 1.66 | ↑ |
| 71 | ESI(-) | Taurodeoxycholate | 498.289 | 442.703 | (M-H)- | KEGG | 4.11 | 1.97 | ↑* |  | - | - | - |
| 72 | ESI(-) | Glycocholate | 464.302 | 406.214 | (M-H)- | KEGG | 3.22 | 1.66 | ↑* |  | - | - | - |
| 73 | ESI(-) | 3,5-Diiodo-4-hydroxyphenylpyruvate | 450.844 | 45.021 | (M+F)- | KEGG | - | - | - |  | 4.00 | 0.56 | ↓ |
| 74 | ESI(-) | Glycodeoxycholate | 448.307 | 451.439 | (M-H)- | KEGG | 4.24 | 1.95 | ↑** |  | 3.05 | 1.53 | ↑* |
| 75 | ESI(-) | Docosahexaenoic acid | 327.233 | 671.838 | (M-H)- | KEGG | 2.71 | 0.61 | ↓* |  | 4.56 | 0.51 | ↓** |
| 76 | ESI(-) | Norlinolenic acid | 280.237 | 691.326 | (M+NH4-2H)- | KEGG | - | - | - |  | 5.47 | 0.41 | ↓** |
| 77 | ESI(-) | Allocholic acid | 407.280 | 447.569 | (M-H)- | HMDB | 1.33 | 1.52 | ↑ |  | - | - | - |
| 78 | ESI(-) | Arachidonic acid | 303.233 | 682.363 | (M-H)- | HMDB | - | - | - |  | 2.99 | 0.64 | ↓* |
| 79 | ESI(-) | Oleic acid | 281.249 | 736.704 | (M-H)- | HMDB | - | - | - |  | 5.00 | 0.48 | ↓** |
| 80 | ESI(-) | Bovinic acid | 279.233 | 691.362 | (M-H)- | HMDB | - | - | - |  | 5.42 | 0.42 | ↓** |
| 81 | ESI(-) | Alpha-Linolenic acid | 277.218 | 657.186 | (M-H)- | HMDB | - | - | - |  | 6.25 | 0.38 | ↓** |
| 82 | ESI(-) | Palmitic acid | 255.233 | 726.302 | (M-H)- | HMDB | - | - | - |  | 2.98 | 0.61 | ↓** |

“-”: VIP or FC does not meet the filtering conditions, ↑: up-regulated, ↓: down-regulated, *: P < 0.05, **P < 0.01.

**Supplementary Table S7.** 0.5 mg BID_Day 7: The identification of differential metabolites based on untargeted metabolomics.

| NO | Ion mode | Identification | mz | rt | Adduct | Database | d7_1h vs d7_0h | | |  | d7_2h vs d7_0h | | |
| --- | --- | --- | --- | --- | --- | --- | --- | --- | --- | --- | --- | --- | --- |
|  |  |  |  |  |  |  | VIP | FC | Trend |  | VIP | FC | Trend |
| 1 | ESI(+) | Docosenoyl-CoA | 1088.437 | 382.746 | (M+H)+ | KEGG | 2.01 | 3.58 | ↑ |  | 2.01 | 3.99 | ↑ |
| 2 | ESI(+) | trans-2-Enoyl-OPC8-CoA | 1088.312 | 382.668 | (M+HCOO+2H)+ | KEGG | - | - | - |  | 1.01 | 3.49 | ↑ |
| 3 | ESI(+) | Retinyl palmitate | 1087.936 | 375.233 | (2M+K)+ | KEGG | - | - | - |  | 2.18 | 3.54 | ↑ |
| 4 | ESI(+) | 3-Oxo-OPC8-CoA | 1080.310 | 384.247 | (M+Na)+ | KEGG | 3.78 | 6.56 | ↑ |  | 3.78 | 7.35 | ↑ |
| 5 | ESI(+) | dITP | 1022.943 | 384.666 | (2M+K)+ | KEGG | 3.01 | 4.43 | ↑ |  | 3.01 | 4.99 | ↑ |
| 6 | ESI(+) | Biotinyl-CoA | 1016.174 | 385.135 | (M+Na)+ | KEGG | - | - | - |  | 1.13 | 4.71 | ↑ |
| 7 | ESI(+) | 20-Hydroxyecdysone | 983.614 | 373.634 | (2M+Na)+ | KEGG | - | - | - |  | 1.72 | 5.85 | ↑ |
| 8 | ESI(+) | Decanoyl-CoA | 966.224 | 384.579 | (M-H+2Na)+ | KEGG | 3.68 | 4.34 | ↑ |  | 3.68 | 4.66 | ↑ |
| 9 | ESI(+) | Zn-Bacteriochlorophyll a | 915.476 | 384.582 | (M+H-2H2O)+ | KEGG | 3.40 | 5.27 | ↑ |  | 3.40 | 5.83 | ↑ |
| 10 | ESI(+) | 1-(5'-Phosphoribosyl)-5-amino-4-(N-succinocarboxamide)-imidazole | 909.157 | 384.910 | (2M+H)+ | KEGG | 3.31 | 5.98 | ↑ |  | 3.31 | 6.72 | ↑ |
| 11 | ESI(+) | 4,4'-Diaponeurosporenic acid | 882.656 | 373.526 | (2M+NH4)+ | KEGG | - | - | - |  | 1.39 | 5.96 | ↑ |
| 12 | ESI(+) | 2,3-Bis-O-(geranylgeranyl)-sn-glycero-1-phospho-L-serine | 804.552 | 808.200 | (M+H)+ | KEGG | 4.23 | 1.77 | ↑ |  | 4.23 | 1.84 | ↑ |
| 13 | ESI(+) | Plastoquinone-9 | 787.604 | 733.916 | (M+K)+ | KEGG | 3.34 | 0.51 | ↓ |  | - | - | - |
| 14 | ESI(+) | (15S)-15-Hydroxy-5,8,11-cis-13-trans-eicosatetraenoate | 658.510 | 668.592 | (2M+NH4)+ | KEGG | - | - | - |  | 1.53 | 1.56 | ↑ |
| 15 | ESI(+) | alpha-Tocopherol acetate | 495.379 | 450.388 | (M+Na)+ | KEGG | 3.84 | 1.94 | ↑* |  | - | - | - |
| 16 | ESI(+) | Cholic acid | 450.321 | 451.330 | (M+CH3CN+H)+ | KEGG | 7.30 | 2.73 | ↑* |  | 7.30 | 1.71 | ↑ |
| 17 | ESI(+) | 3alpha,7alpha-Dihydroxy-12-oxo-5beta-cholanate | 448.306 | 432.057 | (M+CH3CN+H)+ | KEGG | 2.45 | 2.62 | ↑ |  | 2.45 | 2.27 | ↑ |
| 18 | ESI(+) | Ajmaline | 441.074 | 57.515 | (M-2H+3K)+ | KEGG | - | - | - |  | 1.92 | 1.55 | ↑** |
| 19 | ESI(+) | 3-Ketosphingosine | 364.220 | 378.593 | (M-2H+3Na)+ | KEGG | - | - | - |  | 1.51 | 0.61 | ↓** |
| 20 | ESI(+) | Sclareol | 355.285 | 660.976 | (M+HCOO+2H)+ | KEGG | - | - | - |  | 1.78 | 0.65 | ↓ |
| 21 | ESI(+) | Tridihexethyl | 336.327 | 769.197 | (M+NH4)+ | KEGG | 2.75 | 0.63 | ↓ |  | - | - | - |
| 22 | ESI(+) | 1'-Acetoxyeugenol acetate | 331.053 | 60.960 | (M-2H+3Na)+ | KEGG | - | - | - |  | 1.44 | 1.53 | ↑ |
| 23 | ESI(+) | Naphazoline hydrochloride | 323.011 | 58.961 | (M-H+2K)+ | KEGG | 3.07 | 1.55 | ↑* |  | - | - | - |
| 24 | ESI(+) | 2-Amino-4-chloro-4-pentenoic acid | 316.083 | 60.286 | (2M+NH4)+ | KEGG | 4.07 | 2.17 | ↑* |  | - | - | - |
| 25 | ESI(+) | (9Z)-Octadecenoic acid | 283.264 | 737.497 | (M+H)+ | KEGG | - | - | - |  | 1.47 | 0.51 | ↓** |
| 26 | ESI(+) | Gamma-Linolenic acid | 279.232 | 656.456 | (M+H)+ | KEGG | - | - | - |  | 1.11 | 0.49 | ↓** |
| 27 | ESI(+) | Cortisol | 363.217 | 378.637 | (M+H)+ | HMDB | - | - | - |  | 1.44 | 0.56 | ↓** |
| 28 | ESI(+) | Piperine | 286.144 | 475.909 | (M+H)+ | HMDB | 2.71 | 1.92 | ↑* |  | 2.71 | 2.02 | ↑** |
| 29 | ESI(-) | Moxalactam | 1039.185 | 67.893 | (2M-H)- | KEGG | - | - | - |  | 2.00 | 1.51 | ↑ |
| 30 | ESI(-) | Rifamycin S | 716.268 | 391.050 | (M+Na-2H)- | KEGG | 1.16 | 0.50 | ↓ |  | 1.16 | 0.50 | ↓ |
| 31 | ESI(-) | D-Urobilinogen | 606.329 | 385.165 | (M+NH4-2H)- | KEGG | 1.22 | 0.49 | ↓ |  | 1.20 | 0.46 | ↓ |
| 32 | ESI(-) | APC | 599.252 | 424.194 | (M-H2O-H)- | KEGG | 1.33 | 0.36 | ↓ |  | 1.30 | 0.40 | ↓ |
| 33 | ESI(-) | Americine | 582.244 | 446.876 | (M+K-2H)- | KEGG | 1.67 | 0.35 | ↓ |  | 1.54 | 0.32 | ↓ |
| 34 | ESI(-) | Antioside | 568.321 | 397.308 | (M+NH4-2H)- | KEGG | - | - | - |  | 1.04 | 0.59 | ↓ |
| 35 | ESI(-) | Nelfinavir | 566.305 | 393.125 | (M-H)- | KEGG | 1.34 | 0.39 | ↓ |  | 1.39 | 0.41 | ↓ |
| 36 | ESI(-) | 15-Demethoxy-epsilon-rhodomycin | 552.204 | 542.767 | (M-H2O-H)- | KEGG | 1.34 | 0.48 | ↓ |  | 1.54 | 0.54 | ↓ |
| 37 | ESI(-) | 6''-O-Carbamoylkanamycin A | 548.232 | 451.017 | (M+Na-2H)- | KEGG | 1.35 | 1.60 | ↑* |  | - | - | - |
| 38 | ESI(-) | 1D-myo-Inositol 1,3,4,5-tetrakisphosphate | 536.871 | 47.830 | (M+K-2H)- | KEGG | - | - | - |  | 1.30 | 0.43 | ↓ |
| 39 | ESI(-) | Glycochenodeoxycholate 7-sulfate | 528.263 | 422.020 | (M-H)- | KEGG | 2.47 | 1.79 | ↑** |  | - | - | - |
| 40 | ESI(-) | Taurocholate | 514.284 | 391.766 | (M-H)- | KEGG | 1.65 | 1.74 | ↑** |  | - | - | - |
| 41 | ESI(-) | L-Oleandrosyl-oleandolide | 511.291 | 402.722 | (M-H2O-H)- | KEGG | 1.01 | 1.95 | ↑ |  | - | - | - |
| 42 | ESI(-) | Mupirocin | 499.292 | 441.674 | (M-H)- | KEGG | 1.14 | 1.74 | ↑ |  | - | - | - |
| 43 | ESI(-) | Taurodeoxycholate | 498.289 | 442.703 | (M-H)- | KEGG | 4.96 | 3.83 | ↑* |  | 2.43 | 1.86 | ↑* |
| 44 | ESI(-) | Makisterone B | 475.306 | 562.342 | (M-H2O-H)- | KEGG | 1.12 | 0.50 | ↓ |  | 1.18 | 0.54 | ↓ |
| 45 | ESI(-) | Tobramycin | 466.254 | 415.656 | (M-H)- | KEGG | 1.19 | 0.40 | ↓ |  | 1.33 | 0.40 | ↓ |
| 46 | ESI(-) | Cholesterol sulfate | 465.305 | 406.352 | (M-H)- | KEGG | 3.37 | 3.09 | ↑* |  | 2.49 | 2.16 | ↑* |
| 47 | ESI(-) | Glycocholate | 464.302 | 406.214 | (M-H)- | KEGG | 5.34 | 4.41 | ↑* |  | 4.22 | 2.89 | ↑* |
| 48 | ESI(-) | Glycodeoxycholate | 448.307 | 451.439 | (M-H)- | KEGG | 5.08 | 3.51 | ↑* |  | 3.57 | 2.12 | ↑* |
| 49 | ESI(-) | Gentamicin C1a | 448.278 | 511.438 | (M-H)- | KEGG | 1.34 | 0.44 | ↓ |  | 1.31 | 0.43 | ↓ |
| 50 | ESI(-) | Cefixime | 434.023 | 66.096 | (M-H2O-H)- | KEGG | 2.26 | 1.71 | ↑** |  | - | - | - |
| 51 | ESI(-) | Nuatigenin | 429.301 | 562.546 | (M-H)- | KEGG | 1.18 | 0.52 | ↓ |  | 1.16 | 0.50 | ↓ |
| 52 | ESI(-) | Kinetin | 429.154 | 255.868 | (2M-H)- | KEGG | 1.17 | 0.44 | ↓ |  | 1.20 | 0.46 | ↓ |
| 53 | ESI(-) | Didrovaltratum | 405.192 | 380.862 | (M-H2O-H)- | KEGG | 1.22 | 0.39 | ↓ |  | 1.87 | 0.29 | ↓ |
| 54 | ESI(-) | Lovastatin | 385.238 | 442.125 | (M-H2O-H)- | KEGG | 1.64 | 0.47 | ↓ |  | 1.84 | 0.48 | ↓ |
| 55 | ESI(-) | Meloxicam | 350.030 | 255.588 | (M-H)- | KEGG | 1.19 | 0.39 | ↓ |  | 1.36 | 0.36 | ↓ |
| 56 | ESI(-) | Norlinolenic acid | 280.237 | 691.326 | (M+NH4-2H)- | KEGG | - | - | - |  | 2.96 | 0.63 | ↓ |
| 57 | ESI(-) | 3-(4-Hydroxyphenyl)lactate | 181.051 | 261.816 | (M-H)- | KEGG | 1.45 | 0.33 | ↓ |  | 1.44 | 0.33 | ↓ |
| 58 | ESI(-) | Pyrazinoic acid | 160.977 | 47.294 | (M+K-2H)- | KEGG | 1.58 | 0.40 | ↓ |  | 1.76 | 0.44 | ↓ |
| 59 | ESI(-) | Carbon disulfide | 96.922 | 55.436 | (M+Na-2H)- | KEGG | 1.32 | 0.39 | ↓ |  | 1.36 | 0.40 | ↓ |
| 60 | ESI(-) | Deoxycholic acid | 391.285 | 522.588 | (M-H)- | HMDB | - | - | - |  | 1.93 | 1.88 | ↑* |
| 61 | ESI(-) | Leucinic acid | 131.071 | 312.707 | (M-H)- | HMDB | 1.39 | 0.42 | ↓ |  | 1.58 | 0.48 | ↓ |

“-”: VIP or FC does not meet the filtering conditions, ↑: up-regulated, ↓: down-regulated, *: P < 0.05, **P < 0.01.

**Supplementary Table S8.** 1 mg BID_Day 1: The identification of differential metabolites based on untargeted metabolomics.

| NO | Ion mode | Identification | mz | rt | Adduct | Database | d1_1h vs d1_0h | | |  | d1_2h vs d1_0h | | |
| --- | --- | --- | --- | --- | --- | --- | --- | --- | --- | --- | --- | --- | --- |
|  |  |  |  |  |  |  | VIP | FC | Trend |  | VIP | FC | Trend |
| 1 | ESI(+) | Pravastatin | 866.533 | 807.446 | (2M+NH4)+ | KEGG | - | - | - |  | 2.33 | 1.53 | ↑* |
| 2 | ESI(+) | Salmeterol | 848.552 | 810.069 | (2M+NH4)+ | KEGG | 1.39 | 0.59 | ↓ |  | - | - | - |
| 3 | ESI(+) | Petromyzonol | 811.604 | 734.065 | (2M+Na)+ | KEGG | 1.84 | 0.50 | ↓ |  | 1.22 | 0.66 | ↓ |
| 4 | ESI(+) | Plastoquinone-9 | 787.604 | 733.916 | (M+K)+ | KEGG | 4.20 | 0.43 | ↓** |  | 3.32 | 0.61 | ↓ |
| 5 | ESI(+) | S-(PGA1)-glutathione | 682.279 | 413.265 | (M+K)+ | KEGG | - | - | - |  | 1.73 | 0.64 | ↓ |
| 6 | ESI(+) | 3-Hydroxyethylbacteriochlorophyllide a | 681.276 | 413.114 | (M+HCOO+2H)+ | KEGG | - | - | - |  | 1.43 | 0.63 | ↓ |
| 7 | ESI(+) | Ambenonium | 651.145 | 434.775 | (M-2H+3K)+ | KEGG | - | - | - |  | 1.50 | 0.61 | ↓ |
| 8 | ESI(+) | Clobenpropit | 617.179 | 434.546 | (2M+H)+ | KEGG | 1.27 | 0.48 | ↓ |  | 1.14 | 0.23 | ↓ |
| 9 | ESI(+) | 15-Demethoxy-epsilon-rhodomycin | 616.176 | 434.554 | (M-H+2Na)+ | KEGG | - | - | - |  | 2.35 | 0.23 | ↓ |
| 10 | ESI(+) | Graphinone | 593.333 | 462.508 | (2M+H)+ | KEGG | 1.49 | 1.83 | ↑ |  | 1.55 | 2.24 | ↑ |
| 11 | ESI(+) | Rocuronium | 576.410 | 579.940 | (M+HCOO+2H)+ | KEGG | 1.47 | 0.66 | ↓ |  | - | - | - |
| 12 | ESI(+) | Levorphanol | 532.383 | 582.103 | (2M+NH4)+ | KEGG | - | - | - |  | 1.52 | 0.63 | ↓ |
| 13 | ESI(+) | Hentriacontane | 513.419 | 646.069 | (M-H+2K)+ | KEGG | 2.51 | 0.65 | ↓ |  | - | - | - |
| 14 | ESI(+) | alpha-Tocopherol acetate | 495.379 | 450.388 | (M+Na)+ | KEGG | 3.79 | 2.24 | ↑* |  | 3.73 | 2.43 | ↑* |
| 15 | ESI(+) | Netilmicin | 476.306 | 301.881 | (M+H)+ | KEGG | 2.68 | 0.48 | ↓ |  | 1.68 | 0.56 | ↓ |
| 16 | ESI(+) | Montanic acid | 469.392 | 646.448 | (M-H+2Na)+ | KEGG | 1.86 | 0.66 | ↓ |  | - | - | - |
| 17 | ESI(+) | Phylloquinone | 468.389 | 647.150 | (M+NH4)+ | KEGG | 3.11 | 0.53 | ↓** |  | - | - | - |
| 18 | ESI(+) | Cholic acid | 450.321 | 451.330 | (M+CH3CN+H)+ | KEGG | 6.63 | 3.05 | ↑* |  | 6.56 | 3.29 | ↑** |
| 19 | ESI(+) | 3alpha,7alpha-Dihydroxy-12-oxo-5beta-cholanate | 448.306 | 432.057 | (M+CH3CN+H)+ | KEGG | 6.73 | 2.90 | ↑** |  | 5.65 | 2.56 | ↑** |
| 20 | ESI(+) | Ajmaline | 441.074 | 57.515 | (M-2H+3K)+ | KEGG | 2.82 | 1.70 | ↑** |  | 2.50 | 1.64 | ↑** |
| 21 | ESI(+) | Gentamicin C1a | 432.280 | 296.179 | (M+H-H2O)+ | KEGG | 3.84 | 0.42 | ↓ |  | 2.28 | 0.53 | ↓ |
| 22 | ESI(+) | Di(2-ethylhexyl) adipate | 417.319 | 477.068 | (M+HCOO+2H)+ | KEGG | 3.14 | 1.69 | ↑* |  | - | - | - |
| 23 | ESI(+) | Propyl cinnamate | 398.233 | 668.704 | (2M+NH4)+ | KEGG | 2.27 | 0.49 | ↓ |  | 1.93 | 0.54 | ↓ |
| 24 | ESI(+) | Fortimicin A | 388.254 | 289.668 | (M+H-H2O)+ | KEGG | 2.43 | 0.49 | ↓ |  | 1.68 | 0.55 | ↓ |
| 25 | ESI(+) | Tomatidine | 380.337 | 650.343 | (M+H-2H2O)+ | KEGG | 3.66 | 0.50 | ↓* |  | 2.33 | 0.65 | ↓ |
| 26 | ESI(+) | Solasodine | 378.321 | 411.175 | (M+H-2H2O)+ | KEGG | 1.71 | 0.65 | ↓ |  | - | - | - |
| 27 | ESI(+) | Tetrahydrocorticosterone | 368.280 | 425.333 | (M+NH4)+ | KEGG | - | - | - |  | 2.24 | 0.63 | ↓* |
| 28 | ESI(+) | 3-Ketosphingosine | 364.220 | 378.593 | (M-2H+3Na)+ | KEGG | - | - | - |  | 2.34 | 0.62 | ↓ |
| 29 | ESI(+) | (15S)-15-Hydroxy-5,8,11-cis-13-trans-eicosatetraenoate | 338.269 | 691.359 | (M+NH4)+ | KEGG | - | - | - |  | 2.00 | 0.61 | ↓ |
| 30 | ESI(+) | Erucic acid | 321.316 | 649.828 | (M+H-H2O)+ | KEGG | 8.25 | 0.25 | ↓** |  | 3.93 | 0.45 | ↓** |
| 31 | ESI(+) | Tridihexethyl | 319.285 | 652.168 | (M+H)+ | KEGG | 3.17 | 0.56 | ↓ |  | 2.99 | 0.65 | ↓ |
| 32 | ESI(+) | 16-Hydroxypalmitate | 290.269 | 412.748 | (M+NH4)+ | KEGG | - | - | - |  | 2.07 | 0.66 | ↓* |
| 33 | ESI(+) | (9Z)-Octadecenoic acid | 283.264 | 737.497 | (M+H)+ | KEGG | 2.69 | 0.60 | ↓** |  | 5.97 | 0.32 | ↓** |
| 34 | ESI(+) | Gamma-Linolenic acid | 279.232 | 656.456 | (M+H)+ | KEGG | - | - | - |  | 4.38 | 0.42 | ↓** |
| 35 | ESI(+) | Cortisol | 363.217 | 378.637 | (M+H)+ | HMDB | - | - | - |  | 2.40 | 0.63 | ↓ |
| 36 | ESI(-) | Avermectin A1a | 902.524 | 810.172 | (M+NH4-2H)- | KEGG | - | - | - |  | 5.43 | 0.28 | ↓** |
| 37 | ESI(-) | UDP-2-acetamido-3-amino-2,3-dideoxy-alpha-D-glucuronate | 636.086 | 73.909 | (M+NH4-2H)- | KEGG | - | - | - |  | 2.30 | 0.60 | ↓* |
| 38 | ESI(-) | 3-Hydroxyethylchlorophyllide a | 631.242 | 436.460 | (M-H)- | KEGG | 1.48 | 0.64 | ↓ |  | 1.76 | 0.55 | ↓ |
| 39 | ESI(-) | I-Urobilinogen | 591.319 | 462.997 | (M-H)- | KEGG | - | - | - |  | 1.56 | 1.76 | ↑ |
| 40 | ESI(-) | 6''-O-Carbamoylkanamycin A | 548.232 | 451.017 | (M+Na-2H)- | KEGG | 1.56 | 1.51 | ↑ |  | 1.81 | 1.61 | ↑ |
| 41 | ESI(-) | Glycochenodeoxycholate 7-sulfate | 528.263 | 422.020 | (M-H)- | KEGG | 2.55 | 1.55 | ↑ |  | - | - | - |
| 42 | ESI(-) | Taurocholate | 514.284 | 391.766 | (M-H)- | KEGG | 2.55 | 1.97 | ↑ |  | 2.82 | 2.29 | ↑ |
| 43 | ESI(-) | Mupirocin | 499.292 | 441.674 | (M-H)- | KEGG | 1.50 | 1.76 | ↑ |  | 1.69 | 1.96 | ↑ |
| 44 | ESI(-) | Taurodeoxycholate | 498.289 | 442.703 | (M-H)- | KEGG | 6.62 | 4.03 | ↑ |  | 6.21 | 5.08 | ↑* |
| 45 | ESI(-) | Cholesterol sulfate | 465.305 | 406.352 | (M-H)- | KEGG | 4.03 | 2.71 | ↑ |  | 4.71 | 3.56 | ↑ |
| 46 | ESI(-) | Glycocholate | 464.302 | 406.214 | (M-H)- | KEGG | 6.33 | 3.43 | ↑ |  | 6.47 | 4.50 | ↑* |
| 47 | ESI(-) | 3,5-Diiodo-4-hydroxyphenylpyruvate | 450.844 | 45.021 | (M+F)- | KEGG | - | - | - |  | 6.11 | 1.81 | ↑ |
| 48 | ESI(-) | Glycodeoxycholate | 448.307 | 451.439 | (M-H)- | KEGG | 7.30 | 3.29 | ↑* |  | 6.38 | 3.58 | ↑** |
| 49 | ESI(-) | Chenodeoxycholate | 391.285 | 657.364 | (M-H)- | KEGG | 4.12 | 0.48 | ↓** |  | - | - | - |
| 50 | ESI(-) | Digalacturonate | 391.050 | 75.469 | (M+Na-2H)- | KEGG | 2.20 | 0.66 | ↓ |  | - | - | - |
| 51 | ESI(-) | Taxa-4(20),11(12)-dien-5alpha-yl acetate | 329.249 | 693.228 | (M-H)- | KEGG | - | - | - |  | 2.07 | 0.59 | ↓* |
| 52 | ESI(-) | Docosahexaenoic acid | 327.233 | 671.838 | (M-H)- | KEGG | - | - | - |  | 2.56 | 0.57 | ↓** |
| 53 | ESI(-) | (-)-Menthone | 307.265 | 749.046 | (2M-H)- | KEGG | 2.87 | 0.63 | ↓** |  | 4.11 | 0.42 | ↓** |
| 54 | ESI(-) | Norlinolenic acid | 280.237 | 691.326 | (M+NH4-2H)- | KEGG | 2.53 | 0.63 | ↓** |  | 3.96 | 0.43 | ↓** |
| 55 | ESI(-) | Oleic acid | 563.504 | 736.373 | (2M-H)- | HMDB | 3.16 | 0.54 | ↓** |  | 3.71 | 0.43 | ↓* |
| 56 | ESI(-) | Linoleic acid | 559.473 | 691.034 | (2M-H)- | HMDB | 2.91 | 0.62 | ↓* |  | 4.04 | 0.43 | ↓** |
| 57 | ESI(-) | Deoxycholic acid | 391.285 | 479.072 | (M-H)- | HMDB | - | - | - |  | 2.00 | 1.64 | ↑ |
| 58 | ESI(-) | Arachidonic acid | 303.233 | 682.363 | (M-H)- | HMDB | - | - | - |  | 2.69 | 0.60 | ↓** |
| 59 | ESI(-) | Bovinic acid | 279.233 | 691.362 | (M-H)- | HMDB | 2.53 | 0.63 | ↓** |  | 3.78 | 0.44 | ↓** |
| 60 | ESI(-) | Alpha-Linolenic acid | 277.218 | 657.186 | (M-H)- | HMDB | 2.64 | 0.63 | ↓** |  | 4.17 | 0.42 | ↓** |
| 61 | ESI(-) | Palmitic acid | 255.233 | 726.302 | (M-H)- | HMDB | - | - | - |  | 3.72 | 0.45 | ↓** |
| 62 | ESI(-) | Palmitoleic acid | 253.218 | 678.007 | (M-H)- | HMDB | 2.66 | 0.58 | ↓* |  | 4.15 | 0.39 | ↓** |
| 63 | ESI(-) | Myristic acid | 227.202 | 665.450 | (M-H)- | HMDB | 2.37 | 0.63 | ↓* |  | 2.57 | 0.54 | ↓* |

“-”: VIP or FC does not meet the filtering conditions, ↑: up-regulated, ↓: down-regulated, *: P < 0.05, **P < 0.01.

**Supplementary Table S9.** 1 mg BID_Day 7: The identification of differential metabolites based on untargeted metabolomics.

| NO | Ion mode | Identification | mz | rt | Adduct | Database | d7_1h vs d7_0h | | |  | d7_2h vs d7_0h | | |
| --- | --- | --- | --- | --- | --- | --- | --- | --- | --- | --- | --- | --- | --- |
|  |  |  |  |  |  |  | VIP | FC | Trend |  | VIP | FC | Trend |
| 1 | ESI(+) | Taurolithocholate | 967.612 | 381.057 | (2M+H)+ | KEGG | - | - | - |  | 1.75 | 1.52 | ↑* |
| 2 | ESI(+) | Petromyzonol | 811.603 | 656.376 | (2M+Na)+ | KEGG | - | - | - |  | 3.51 | 0.49 | ↓* |
| 3 | ESI(+) | 2,3-Bis-O-(geranylgeranyl)-sn-glycero-1-phospho-L-serine | 804.552 | 808.200 | (M+H)+ | KEGG | 1.90 | 1.51 | ↑ |  | - | - | - |
| 4 | ESI(+) | 6-Thioxanthine 5'-monophosphate | 783.026 | 57.443 | (2M+Na)+ | KEGG | - | - | - |  | 2.11 | 1.58 | ↑** |
| 5 | ESI(+) | S-(PGA1)-glutathione | 682.279 | 413.265 | (M+K)+ | KEGG | 1.07 | 1.76 | ↑ |  | - | - | - |
| 6 | ESI(+) | Clobenpropit | 617.179 | 434.546 | (2M+H)+ | KEGG | 1.42 | 2.47 | ↑ |  | - | - | - |
| 7 | ESI(+) | 15-Demethoxy-epsilon-rhodomycin | 616.176 | 434.554 | (M-H+2Na)+ | KEGG | 1.05 | 2.21 | ↑ |  | - | - | - |
| 8 | ESI(+) | L-Urobilinogen | 597.363 | 460.602 | (M+H)+ | KEGG | 2.47 | 1.96 | ↑* |  | 2.44 | 2.04 | ↑ |
| 9 | ESI(+) | Graphinone | 593.333 | 462.508 | (2M+H)+ | KEGG | 2.77 | 1.95 | ↑ |  | 6.92 | 5.62 | ↑* |
| 10 | ESI(+) | D-Urobilinogen | 591.317 | 346.610 | (M+H)+ | KEGG | - | - | - |  | 3.66 | 3.87 | ↑ |
| 11 | ESI(+) | alpha-Tocopherol acetate | 495.379 | 450.388 | (M+Na)+ | KEGG | 5.96 | 3.56 | ↑* |  | 4.30 | 2.17 | ↑ |
| 12 | ESI(+) | Cholic acid | 450.321 | 451.330 | (M+CH3CN+H)+ | KEGG | 9.25 | 6.83 | ↑* |  | 5.75 | 2.39 | ↑ |
| 13 | ESI(+) | 3alpha,7alpha-Dihydroxy-12-oxo-5beta-cholanate | 448.306 | 432.057 | (M+CH3CN+H)+ | KEGG | 4.01 | 3.23 | ↑ |  | 2.98 | 2.23 | ↑ |
| 14 | ESI(+) | Ajmaline | 441.074 | 57.515 | (M-2H+3K)+ | KEGG | - | - | - |  | 2.28 | 1.65 | ↑** |
| 15 | ESI(+) | Di(2-ethylhexyl) adipate | 417.319 | 477.068 | (M+HCOO+2H)+ | KEGG | 2.46 | 1.79 | ↑** |  | - | - | - |
| 16 | ESI(+) | Solasodine | 378.321 | 411.175 | (M+H-2H2O)+ | KEGG | 1.69 | 1.58 | ↑ |  | - | - | - |
| 17 | ESI(+) | Propofol | 357.279 | 520.791 | (2M+H)+ | KEGG | 1.81 | 1.86 | ↑ |  | 3.69 | 2.21 | ↑** |
| 18 | ESI(+) | (15S)-15-Hydroxy-5,8,11-cis-13-trans-eicosatetraenoate | 338.269 | 691.359 | (M+NH4)+ | KEGG | - | - | - |  | 2.35 | 0.61 | ↓ |
| 19 | ESI(+) | (9Z)-Octadecenoic acid | 283.264 | 737.497 | (M+H)+ | KEGG | - | - | - |  | 3.70 | 0.53 | ↓** |
| 20 | ESI(+) | Gamma-Linolenic acid | 279.232 | 656.456 | (M+H)+ | KEGG | - | - | - |  | 3.62 | 0.49 | ↓** |
| 21 | ESI(+) | Hexadecanal | 258.279 | 444.012 | (M+NH4)+ | KEGG | - | - | - |  | 1.35 | 1.57 | ↑ |
| 22 | ESI(-) | L-Urobilin | 593.334 | 460.232 | (M-H)- | KEGG | - | - | - |  | 2.73 | 1.95 | ↑ |
| 23 | ESI(-) | I-Urobilinogen | 591.319 | 462.997 | (M-H)- | KEGG | - | - | - |  | 4.70 | 3.93 | ↑ |
| 24 | ESI(-) | 6''-O-Carbamoylkanamycin A | 548.232 | 451.017 | (M+Na-2H)- | KEGG | 2.64 | 2.03 | ↑ |  | - | - | - |
| 25 | ESI(-) | L-Olivosyl-oleandolide | 515.287 | 390.278 | (M-H)- | KEGG | 2.12 | 1.61 | ↑ |  | - | - | - |
| 26 | ESI(-) | Taurocholate | 514.284 | 391.766 | (M-H)- | KEGG | 4.10 | 3.04 | ↑ |  | - | - | - |
| 27 | ESI(-) | Mupirocin | 499.292 | 441.674 | (M-H)- | KEGG | 4.11 | 3.52 | ↑ |  | 1.57 | 1.51 | ↑ |
| 28 | ESI(-) | Taurodeoxycholate | 498.289 | 442.703 | (M-H)- | KEGG | 9.31 | 7.83 | ↑* |  | 7.38 | 3.43 | ↑ |
| 29 | ESI(-) | Vindoline | 472.245 | 448.874 | (M+NH4-2H)- | KEGG | - | - | - |  | 1.99 | 1.64 | ↑ |
| 30 | ESI(-) | Cholesterol sulfate | 465.305 | 406.352 | (M-H)- | KEGG | 6.49 | 4.28 | ↑ |  | 4.77 | 1.92 | ↑ |
| 31 | ESI(-) | Glycocholate | 464.302 | 406.214 | (M-H)- | KEGG | 8.22 | 4.88 | ↑ |  | 7.00 | 2.20 | ↑ |
| 32 | ESI(-) | Glycodeoxycholate | 448.307 | 451.439 | (M-H)- | KEGG | 7.89 | 4.81 | ↑** |  | 6.13 | 2.30 | ↑ |
| 33 | ESI(-) | Taxa-4(20),11(12)-dien-5alpha-yl acetate | 329.249 | 693.228 | (M-H)- | KEGG | - | - | - |  | 2.87 | 0.56 | ↓** |
| 34 | ESI(-) | (-)-Menthone | 307.265 | 749.046 | (2M-H)- | KEGG | - | - | - |  | 3.08 | 0.55 | ↓** |
| 35 | ESI(-) | Norlinolenic acid | 280.237 | 691.326 | (M+NH4-2H)- | KEGG | - | - | - |  | 3.89 | 0.46 | ↓** |
| 36 | ESI(-) | Linoleic acid | 559.473 | 691.034 | (2M-H)- | HMDB | - | - | - |  | 4.49 | 0.41 | ↓** |
| 37 | ESI(-) | Deoxycholic acid | 391.285 | 522.588 | (M-H)- | HMDB | 2.86 | 2.10 | ↑* |  | 3.94 | 2.26 | ↑** |
| 38 | ESI(-) | Arachidonic acid | 303.233 | 682.363 | (M-H)- | HMDB | - | - | - |  | 2.35 | 0.63 | ↓** |
| 39 | ESI(-) | Oleic acid | 281.249 | 736.704 | (M-H)- | HMDB | - | - | - |  | 3.08 | 0.53 | ↓** |
| 40 | ESI(-) | Bovinic acid | 279.233 | 691.362 | (M-H)- | HMDB | - | - | - |  | 3.86 | 0.46 | ↓** |
| 41 | ESI(-) | Alpha-Linolenic acid | 277.218 | 657.186 | (M-H)- | HMDB | - | - | - |  | 3.79 | 0.45 | ↓** |
| 42 | ESI(-) | Palmitic acid | 255.233 | 726.302 | (M-H)- | HMDB | - | - | - |  | 2.94 | 0.54 | ↓** |
| 43 | ESI(-) | Palmitoleic acid | 253.218 | 678.007 | (M-H)- | HMDB | - | - | - |  | 3.58 | 0.49 | ↓** |
| 44 | ESI(-) | Myristic acid | 227.202 | 665.450 | (M-H)- | HMDB | 1.56 | 0.66 | ↓* |  | 3.02 | 0.52 | ↓** |

“-”: VIP or FC does not meet the filtering conditions, ↑: up-regulated, ↓: down-regulated, *: P < 0.05, **P < 0.01.

**Supplementary Table S10.** 1 mg QD_Day 1: The identification of differential metabolites based on non-targeted metabolomics.

| NO | Ion mode | Identification | mz | rt | Adduct | Database | d1_1h vs d1_0h | | |  | d1_2h vs d1_0h | | |
| --- | --- | --- | --- | --- | --- | --- | --- | --- | --- | --- | --- | --- | --- |
|  |  |  |  |  |  |  | VIP | FC | Trend |  | VIP | FC | Trend |
| 1 | ESI(+) | Petromyzonol | 811.604 | 734.065 | (2M+Na)+ | KEGG | 3.02 | 0.51 | ↓ |  | - | - | - |
| 2 | ESI(+) | 2,3-Bis-O-(geranylgeranyl)-sn-glycero-1-phospho-L-serine | 804.552 | 808.200 | (M+H)+ | KEGG | 3.36 | 1.95 | ↑ |  | - | - | - |
| 3 | ESI(+) | Clobenpropit | 617.179 | 434.546 | (2M+H)+ | KEGG | 1.41 | 0.42 | ↓ |  | 1.63 | 0.29 | ↓ |
| 4 | ESI(+) | 15-Demethoxy-epsilon-rhodomycin | 616.176 | 434.554 | (M-H+2Na)+ | KEGG | 1.99 | 0.45 | ↓ |  | 2.33 | 0.33 | ↓ |
| 5 | ESI(+) | Cholic acid | 450.321 | 451.330 | (M+CH3CN+H)+ | KEGG | 1.20 | 1.51 | ↑* |  | 1.34 | 1.61 | ↑* |
| 6 | ESI(+) | Glycodeoxycholate | 414.300 | 450.528 | (M+H-2H2O)+ | KEGG | - | - | - |  | 1.15 | 1.67 | ↑* |
| 7 | ESI(+) | Erucic acid | 321.316 | 649.828 | (M+H-H2O)+ | KEGG | 1.08 | 0.62 | ↓* |  | 1.35 | 0.56 | ↓* |
| 8 | ESI(+) | Gamma-Linolenic acid | 279.232 | 656.456 | (M+H)+ | KEGG | - | - | - |  | 1.48 | 0.56 | ↓** |
| 9 | ESI(+) | Piperine | 286.144 | 475.909 | (M+H)+ | HMDB | 1.39 | 2.14 | ↑* |  | 1.36 | 1.96 | ↑* |
| 10 | ESI(-) | Avermectin B1b | 874.493 | 810.975 | (M+NH4-2H)- | KEGG | - | - | - |  | 1.72 | 1.55 | ↑ |
| 11 | ESI(-) | 2'''-N-Acetyl-6'''-deamino-6'''-hydroxyparomomycin II | 679.263 | 413.723 | (M+Na-2H)- | KEGG | - | - | - |  | 1.63 | 0.45 | ↓ |
| 12 | ESI(-) | CMP-N-glycoloylneuraminate | 667.094 | 62.680 | (M+K-2H)- | KEGG | 1.26 | 0.64 | ↓* |  | - | - | - |
| 13 | ESI(-) | 3-Hydroxyethylchlorophyllide a | 631.242 | 436.460 | (M-H)- | KEGG | - | - | - |  | 1.49 | 0.53 | ↓ |
| 14 | ESI(-) | I-Urobilinogen | 591.319 | 462.997 | (M-H)- | KEGG | 1.29 | 1.61 | ↑ |  | 1.66 | 1.67 | ↑ |
| 15 | ESI(-) | 3,5-Diiodo-4-hydroxyphenylpyruvate | 450.844 | 45.021 | (M+F)- | KEGG | - | - | - |  | 1.74 | 1.53 | ↑ |
| 16 | ESI(-) | Glycocholate | 446.291 | 432.370 | (M-H2O-H)- | KEGG | 1.68 | 1.71 | ↑ |  | - | - | - |
| 17 | ESI(-) | Chenodeoxycholate | 391.285 | 657.364 | (M-H)- | KEGG | 1.78 | 0.56 | ↓* |  | - | - | - |
| 18 | ESI(-) | Docosahexaenoic acid | 327.233 | 671.838 | (M-H)- | KEGG | - | - | - |  | 2.23 | 0.56 | ↓** |
| 19 | ESI(-) | Estazolam | 315.042 | 66.620 | (M+Na-2H)- | KEGG | 1.52 | 0.60 | ↓ |  | - | - | - |
| 20 | ESI(-) | Norlinolenic acid | 280.237 | 691.326 | (M+NH4-2H)- | KEGG | - | - | - |  | 3.03 | 0.51 | ↓** |
| 21 | ESI(-) | Arachidonic acid | 303.233 | 682.363 | (M-H)- | HMDB | 1.40 | 0.66 | ↓* |  | 2.09 | 0.54 | ↓** |
| 22 | ESI(-) | Oleic acid | 281.249 | 736.704 | (M-H)- | HMDB | - | - | - |  | 7.05 | 0.53 | ↓** |
| 23 | ESI(-) | Bovinic acid | 279.233 | 691.362 | (M-H)- | HMDB | - | - | - |  | 6.71 | 0.51 | ↓** |
| 24 | ESI(-) | Alpha-Linolenic acid | 277.218 | 657.186 | (M-H)- | HMDB | - | - | - |  | 3.21 | 0.41 | ↓** |
| 25 | ESI(-) | Palmitic acid | 255.233 | 726.302 | (M-H)- | HMDB | - | - | - |  | 5.48 | 0.60 | ↓** |
| 26 | ESI(-) | Palmitoleic acid | 253.218 | 678.007 | (M-H)- | HMDB | - | - | - |  | 1.65 | 0.59 | ↓* |

“-”: VIP or FC does not meet the filtering conditions, ↑: up-regulated, ↓: down-regulated, *: P < 0.05, **P < 0.01.

**Supplementary Table S11.** 1 mg QD_Day 7: The identification of differential metabolites based on untargeted metabolomics.

| NO | Ion mode | Identification | mz | rt | Adduct | Database | d7_1h vs d7_0h | | |  | d7_2h vs d7_0h | | |
| --- | --- | --- | --- | --- | --- | --- | --- | --- | --- | --- | --- | --- | --- |
|  |  |  |  |  |  |  | VIP | FC | Trend |  | VIP | FC | Trend |
| 1 | ESI(+) | Ubiquinone-9 | 812.670 | 813.561 | (M+NH4)+ | KEGG | - | - | - |  | 1.78 | 0.66 | ↓ |
| 2 | ESI(+) | Petromyzonol | 811.604 | 691.968 | (2M+Na)+ | KEGG | - | - | - |  | 2.75 | 1.56 | ↑ |
| 3 | ESI(+) | 2,3-Bis-O-(geranylgeranyl)-sn-glycero-1-phospho-L-serine | 804.552 | 808.200 | (M+H)+ | KEGG | 4.49 | 2.36 | ↑ |  | 4.85 | 3.18 | ↑ |
| 4 | ESI(+) | Plastoquinone-9 | 787.604 | 758.464 | (M+K)+ | KEGG | 6.57 | 0.48 | ↓ |  | 5.85 | 0.50 | ↓ |
| 5 | ESI(+) | 15-Demethoxy-epsilon-rhodomycin | 616.176 | 434.554 | (M-H+2Na)+ | KEGG | - | - | - |  | 1.23 | 0.64 | ↓ |
| 6 | ESI(+) | APC | 601.265 | 328.231 | (M+H-H2O)+ | KEGG | 1.74 | 0.48 | ↓ |  | 1.68 | 0.50 | ↓ |
| 7 | ESI(+) | Graphinone | 593.333 | 462.508 | (2M+H)+ | KEGG | - | - | - |  | 1.22 | 1.94 | ↑ |
| 8 | ESI(+) | Cholic acid | 450.321 | 451.330 | (M+CH3CN+H)+ | KEGG | 2.65 | 2.39 | ↑** |  | 2.35 | 2.38 | ↑* |
| 9 | ESI(+) | Propofol | 357.279 | 520.791 | (2M+H)+ | KEGG | - | - | - |  | 1.04 | 1.53 | ↑* |
| 10 | ESI(+) | Erucic acid | 321.316 | 649.828 | (M+H-H2O)+ | KEGG | - | - | - |  | 1.05 | 0.55 | ↓ |
| 11 | ESI(-) | I-Urobilinogen | 591.319 | 462.997 | (M-H)- | KEGG | - | - | - |  | 1.68 | 1.90 | ↑ |
| 12 | ESI(-) | Mupirocin | 499.292 | 441.674 | (M-H)- | KEGG | - | - | - |  | 1.83 | 2.34 | ↑* |
| 13 | ESI(-) | Taurodeoxycholate | 498.289 | 442.703 | (M-H)- | KEGG | 1.75 | 1.82 | ↑* |  | 2.70 | 2.81 | ↑* |
| 14 | ESI(-) | Vindoline | 472.245 | 448.874 | (M+NH4-2H)- | KEGG | - | - | - |  | 1.54 | 1.71 | ↑* |
| 15 | ESI(-) | Cholesterol sulfate | 465.305 | 406.352 | (M-H)- | KEGG | - | - | - |  | 1.88 | 2.32 | ↑ |
| 16 | ESI(-) | Glycocholate | 464.302 | 406.214 | (M-H)- | KEGG | - | - | - |  | 3.45 | 2.36 | ↑ |
| 17 | ESI(-) | Glycodeoxycholate | 448.307 | 451.439 | (M-H)- | KEGG | 6.10 | 1.63 | ↑* |  | 7.45 | 2.33 | ↑* |
| 18 | ESI(-) | Deoxycholic acid | 391.285 | 479.072 | (M-H)- | HMDB | - | - | - |  | 2.23 | 1.63 | ↑* |
| 19 | ESI(-) | Oleic acid | 281.249 | 736.704 | (M-H)- | HMDB | - | - | - |  | 4.60 | 0.66 | ↓** |
| 20 | ESI(-) | Palmitoleic acid | 253.218 | 678.007 | (M-H)- | HMDB | - | - | - |  | 1.32 | 0.62 | ↓** |

“-”: VIP or FC does not meet the filtering conditions, ↑: up-regulated, ↓: down-regulated, *: P < 0.05, **P < 0.01.

**Supplementary Table S12.** 2 mg QD_Day 1: The identification of differential metabolites based on untargeted metabolomics.

| NO | Ion mode | Identification | mz | rt | Adduct | Database | d1_1h vs d1_0h | | |  | d1_2h vs d1_0h | | |
| --- | --- | --- | --- | --- | --- | --- | --- | --- | --- | --- | --- | --- | --- |
|  |  |  |  |  |  |  | VIP | FC | Trend |  | VIP | FC | Trend |
| 1 | ESI(+) | beta-Carotene | 1073.928 | 374.528 | (2M+H)+ | KEGG | - | - | - |  | 1.02 | 3.01 | ↑ |
| 2 | ESI(+) | Ubiquinone-9 | 812.670 | 813.561 | (M+NH4)+ | KEGG | - | - | - |  | 1.69 | 1.78 | ↑ |
| 3 | ESI(+) | Petromyzonol | 811.604 | 734.065 | (2M+Na)+ | KEGG | 3.82 | 1.97 | ↑* |  | 3.74 | 1.78 | ↑ |
| 4 | ESI(+) | Calcidiol | 801.683 | 813.299 | (2M+H)+ | KEGG | - | - | - |  | 1.18 | 1.79 | ↑* |
| 5 | ESI(+) | Plastoquinone-9 | 787.604 | 787.898 | (M+K)+ | KEGG | 3.02 | 0.65 | ↓ |  | 3.69 | 0.66 | ↓ |
| 6 | ESI(+) | Clobenpropit | 617.179 | 434.546 | (2M+H)+ | KEGG | 1.33 | 0.43 | ↓ |  | - | - | - |
| 7 | ESI(+) | 15-Demethoxy-epsilon-rhodomycin | 616.176 | 434.554 | (M-H+2Na)+ | KEGG | 1.92 | 0.47 | ↓ |  | - | - | - |
| 8 | ESI(+) | APC | 601.265 | 328.231 | (M+H-H2O)+ | KEGG | 1.51 | 3.16 | ↑ |  | 2.19 | 3.59 | ↑ |
| 9 | ESI(+) | alpha-Tocopherol acetate | 495.379 | 450.388 | (M+Na)+ | KEGG | 1.17 | 3.20 | ↑* |  | 1.54 | 3.75 | ↑** |
| 10 | ESI(+) | Cholic acid | 450.321 | 451.330 | (M+CH3CN+H)+ | KEGG | 3.98 | 5.03 | ↑* |  | 5.26 | 6.72 | ↑* |
| 11 | ESI(+) | 3alpha,7alpha-Dihydroxy-12-oxo-5beta-cholanate | 448.306 | 432.057 | (M+CH3CN+H)+ | KEGG | - | - | - |  | 1.09 | 2.31 | ↑* |
| 12 | ESI(+) | Propofol | 357.279 | 520.791 | (2M+H)+ | KEGG | - | - | - |  | 2.02 | 3.25 | ↑ |
| 13 | ESI(+) | Erucic acid | 321.316 | 649.828 | (M+H-H2O)+ | KEGG | - | - | - |  | 1.54 | 0.54 | ↓* |
| 14 | ESI(+) | Gamma-Linolenic acid | 279.232 | 656.456 | (M+H)+ | KEGG | - | - | - |  | 1.36 | 0.58 | ↓** |
| 15 | ESI(-) | Ethylmorphine | 625.344 | 398.827 | (2M-H)- | KEGG | - | - | - |  | 1.88 | 1.51 | ↑** |
| 16 | ESI(-) | L-Urobilin | 593.334 | 460.232 | (M-H)- | KEGG | 1.13 | 1.75 | ↑ |  | 1.19 | 1.74 | ↑ |
| 17 | ESI(-) | 6''-O-Carbamoylkanamycin A | 548.232 | 451.017 | (M+Na-2H)- | KEGG | 1.33 | 2.20 | ↑ |  | 1.62 | 2.72 | ↑* |
| 18 | ESI(-) | Taurocholate | 514.284 | 391.766 | (M-H)- | KEGG | - | - | - |  | 1.57 | 1.98 | ↑* |
| 19 | ESI(-) | L-Oleandrosyl-oleandolide | 511.291 | 402.722 | (M-H2O-H)- | KEGG | 1.98 | 1.66 | ↑ |  | 2.20 | 1.84 | ↑ |
| 20 | ESI(-) | Mupirocin | 499.292 | 441.674 | (M-H)- | KEGG | 1.57 | 2.25 | ↑ |  | 1.99 | 3.28 | ↑ |
| 21 | ESI(-) | Taurodeoxycholate | 498.289 | 442.703 | (M-H)- | KEGG | 3.36 | 4.22 | ↑* |  | 4.28 | 6.78 | ↑* |
| 22 | ESI(-) | Cholesterol sulfate | 465.305 | 406.352 | (M-H)- | KEGG | 2.35 | 2.94 | ↑* |  | 3.05 | 4.30 | ↑* |
| 23 | ESI(-) | Glycocholate | 464.302 | 406.214 | (M-H)- | KEGG | 4.67 | 4.79 | ↑* |  | 5.75 | 7.10 | ↑* |
| 24 | ESI(-) | 3,5-Diiodo-4-hydroxyphenylpyruvate | 450.844 | 45.021 | (M+F)- | KEGG | 1.45 | 2.65 | ↑ |  | - | - | - |
| 25 | ESI(-) | Glycodeoxycholate | 448.307 | 451.439 | (M-H)- | KEGG | 11.63 | 5.07 | ↑* |  | 12.38 | 6.15 | ↑* |
| 26 | ESI(-) | Docosahexaenoic acid | 327.233 | 671.838 | (M-H)- | KEGG | 1.35 | 0.63 | ↓ |  | 1.91 | 0.43 | ↓ |
| 27 | ESI(-) | (-)-Menthone | 307.265 | 749.046 | (2M-H)- | KEGG | - | - | - |  | 1.01 | 0.43 | ↓ |
| 28 | ESI(-) | Norlinolenic acid | 280.237 | 691.326 | (M+NH4-2H)- | KEGG | - | - | - |  | 2.35 | 0.57 | ↓** |
| 29 | ESI(-) | Linoleic acid | 559.473 | 691.034 | (2M-H)- | HMDB | - | - | - |  | 1.71 | 0.50 | ↓* |
| 30 | ESI(-) | Allocholic acid | 407.280 | 447.569 | (M-H)- | HMDB | 1.08 | 1.88 | ↑ |  | - | - | - |
| 31 | ESI(-) | Deoxycholic acid | 391.285 | 522.588 | (M-H)- | HMDB | 1.35 | 1.65 | ↑ |  | 2.55 | 2.98 | ↑* |
| 32 | ESI(-) | Arachidonic acid | 303.233 | 682.363 | (M-H)- | HMDB | - | - | - |  | 1.46 | 0.56 | ↓ |
| 33 | ESI(-) | Oleic acid | 281.249 | 736.704 | (M-H)- | HMDB | - | - | - |  | 5.68 | 0.57 | ↓** |
| 34 | ESI(-) | Bovinic acid | 279.233 | 691.362 | (M-H)- | HMDB | - | - | - |  | 5.04 | 0.58 | ↓** |
| 35 | ESI(-) | Alpha-Linolenic acid | 277.218 | 657.186 | (M-H)- | HMDB | - | - | - |  | 1.83 | 0.50 | ↓ |
| 36 | ESI(-) | Palmitic acid | 255.233 | 726.302 | (M-H)- | HMDB | - | - | - |  | 4.28 | 0.57 | ↓** |
| 37 | ESI(-) | Palmitoleic acid | 253.218 | 678.007 | (M-H)- | HMDB | - | - | - |  | 1.37 | 0.49 | ↓ |

“-”: VIP or FC does not meet the filtering conditions, ↑: up-regulated, ↓: down-regulated, *: P < 0.05, **P < 0.01.

**Supplementary Table S13.** 2 mg QD_Day 7: The identification of differential metabolites based on untargeted metabolomics.

| NO | Ion mode | Identification | mz | rt | Adduct | Database | d7_1h vs d7_0h | | |  | d7_2h vs d7_0h | | |
| --- | --- | --- | --- | --- | --- | --- | --- | --- | --- | --- | --- | --- | --- |
|  |  |  |  |  |  |  | VIP | FC | Trend |  | VIP | FC | Trend |
| 1 | ESI(+) | beta-Carotene | 1073.928 | 374.528 | (2M+H)+ | KEGG | 1.66 | 7.85 | ↑ |  | 1.14 | 4.30 | ↑ |
| 2 | ESI(+) | Hyperforin | 1073.802 | 374.435 | (2M+H)+ | KEGG | - | - | - |  | 1.16 | 3.67 | ↑ |
| 3 | ESI(+) | Piperacillin | 1073.302 | 374.426 | (2M+K)+ | KEGG | - | - | - |  | 1.11 | 3.11 | ↑ |
| 4 | ESI(+) | UDP-L-Ara4N | 1071.119 | 385.480 | (2M+H)+ | KEGG | - | - | - |  | 1.08 | 3.71 | ↑ |
| 5 | ESI(+) | dTDP-4-oxo-2-deoxy-beta-L-xylose | 1071.056 | 385.303 | (2M+K)+ | KEGG | - | - | - |  | 1.08 | 3.30 | ↑ |
| 6 | ESI(+) | Undecaprenyl phosphate alpha-L-Ara4N | 1016.675 | 533.074 | (M+K)+ | KEGG | 1.01 | 1.56 | ↑* |  | - | - | - |
| 7 | ESI(+) | Tylosin | 954.492 | 374.377 | (M+K)+ | KEGG | - | - | - |  | 1.10 | 3.98 | ↑ |
| 8 | ESI(+) | Nonanoyl-CoA | 954.269 | 374.386 | (M+HCOO+2H)+ | KEGG | - | - | - |  | 1.12 | 3.68 | ↑ |
| 9 | ESI(+) | 2-Succinylbenzoyl-CoA | 954.158 | 374.356 | (M+H-H2O)+ | KEGG | 1.08 | 3.88 | ↑ |  | 1.08 | 3.93 | ↑ |
| 10 | ESI(+) | 24,25-Dihydrolanosterol | 874.851 | 372.982 | (2M+NH4)+ | KEGG | - | - | - |  | 1.09 | 4.96 | ↑ |
| 11 | ESI(+) | Fortimicin A | 828.552 | 807.493 | (2M+NH4)+ | KEGG | 2.05 | 0.63 | ↓ |  | - | - | - |
| 12 | ESI(+) | Ubiquinone-9 | 812.670 | 813.561 | (M+NH4)+ | KEGG | - | - | - |  | 1.07 | 0.66 | ↓ |
| 13 | ESI(+) | Petromyzonol | 811.603 | 656.376 | (2M+Na)+ | KEGG | 4.56 | 1.67 | ↑ |  | - | - | - |
| 14 | ESI(+) | 2,3-Bis-O-(geranylgeranyl)-sn-glycero-1-phospho-L-serine | 804.552 | 808.200 | (M+H)+ | KEGG | 2.22 | 1.52 | ↑* |  | - | - | - |
| 15 | ESI(+) | Plastoquinone-9 | 787.604 | 758.464 | (M+K)+ | KEGG | - | - | - |  | 5.40 | 0.46 | ↓* |
| 16 | ESI(+) | Graphinone | 593.333 | 462.508 | (2M+H)+ | KEGG | - | - | - |  | 1.36 | 4.15 | ↑* |
| 17 | ESI(+) | alpha-Tocopherol acetate | 495.379 | 450.388 | (M+Na)+ | KEGG | 1.24 | 3.72 | ↑** |  | 1.12 | 3.68 | ↑** |
| 18 | ESI(+) | Cholic acid | 450.321 | 451.330 | (M+CH3CN+H)+ | KEGG | 3.69 | 5.32 | ↑** |  | 3.52 | 6.85 | ↑ |
| 19 | ESI(+) | EDTA | 315.080 | 60.134 | (M+Na)+ | KEGG | 7.15 | 1.66 | ↑* |  | 5.01 | 1.67 | ↑ |
| 20 | ESI(+) | Piperine | 286.144 | 475.909 | (M+H)+ | HMDB | 1.14 | 0.51 | ↓ |  | - | - | - |
| 21 | ESI(-) | Ethylmorphine | 625.344 | 398.827 | (2M-H)- | KEGG | - | - | - |  | 1.52 | 1.56 | ↑** |
| 22 | ESI(-) | L-Urobilin | 593.334 | 460.232 | (M-H)- | KEGG | - | - | - |  | 1.40 | 2.40 | ↑ |
| 23 | ESI(-) | I-Urobilinogen | 591.319 | 462.997 | (M-H)- | KEGG | 1.14 | 1.86 | ↑ |  | 1.99 | 2.76 | ↑* |
| 24 | ESI(-) | 6''-O-Carbamoylkanamycin A | 548.232 | 451.017 | (M+Na-2H)- | KEGG | 1.99 | 3.46 | ↑** |  | 1.68 | 3.43 | ↑* |
| 25 | ESI(-) | L-Olivosyl-oleandolide | 515.287 | 390.278 | (M-H)- | KEGG | - | - | - |  | 1.03 | 2.41 | ↑* |
| 26 | ESI(-) | Taurocholate | 514.284 | 391.766 | (M-H)- | KEGG | 1.81 | 2.01 | ↑** |  | 1.94 | 2.52 | ↑* |
| 27 | ESI(-) | L-Oleandrosyl-oleandolide | 511.291 | 402.722 | (M-H2O-H)- | KEGG | 2.40 | 2.23 | ↑ |  | 2.04 | 2.17 | ↑* |
| 28 | ESI(-) | Mupirocin | 499.292 | 441.674 | (M-H)- | KEGG | 2.10 | 3.26 | ↑** |  | 1.85 | 3.88 | ↑* |
| 29 | ESI(-) | Taurodeoxycholate | 498.289 | 442.703 | (M-H)- | KEGG | 4.32 | 6.21 | ↑** |  | 3.76 | 7.49 | ↑* |
| 30 | ESI(-) | Cholesterol sulfate | 465.305 | 406.352 | (M-H)- | KEGG | 3.59 | 4.74 | ↑* |  | 3.63 | 5.40 | ↑** |
| 31 | ESI(-) | Glycocholate | 464.302 | 406.214 | (M-H)- | KEGG | 6.56 | 5.61 | ↑* |  | 6.59 | 6.35 | ↑** |
| 32 | ESI(-) | 3,5-Diiodo-4-hydroxyphenylpyruvate | 450.844 | 45.021 | (M+F)- | KEGG | - | - | - |  | 1.02 | 0.50 | ↓ |
| 33 | ESI(-) | Glycodeoxycholate | 448.307 | 451.439 | (M-H)- | KEGG | 13.11 | 5.02 | ↑** |  | 11.28 | 5.03 | ↑** |
| 34 | ESI(-) | Alatolide | 369.174 | 447.419 | (M+F)- | KEGG | - | - | - |  | 4.52 | 1.53 | ↑ |
| 35 | ESI(-) | Umbelliferone | 178.051 | 297.040 | (M+NH4-2H)- | KEGG | - | - | - |  | 1.25 | 1.74 | ↑** |
| 36 | ESI(-) | Deoxycholic acid | 391.285 | 522.588 | (M-H)- | HMDB | 1.78 | 1.93 | ↑** |  | 1.97 | 2.67 | ↑** |
| 37 | ESI(-) | Indoxyl sulfate | 212.003 | 292.730 | (M-H)- | HMDB | - | - | - |  | 2.61 | 1.93 | ↑* |

“-”: VIP or FC does not meet the filtering conditions, ↑: up-regulated, ↓: down-regulated, *: P < 0.05, **P < 0.01.
