# Supplementary material for: Effect of SY009, a novel SGLT1 inhibitor, on the plasma metabolome and bile acids in patients with type 2 diabetes mellitus
Source: Front Endocrinol (Lausanne). 2025 Jan 28;16:1487058. doi: 10.3389/fendo.2025.1487058 (PMC11810745; doi:10.3389/fendo.2025.1487058)
Supplement: Supplementary file 2 [file DataSheet2.docx]

**Inclusion and Exclusion Criteria**

Inclusion Criteria:

- body weight of male ≥ 50kg, female ≥ 45kg, and body mass index (BMI) between 18.0 and 35.0 kg / m2 (including the threshold value) at screening;
- Have T2DM prior to entering the trial based on the disease diagnostic criteria (WHO, 1999), and currently being treated with diet and exercise only for at last 12 weeks , or no systemic treatment of diabetes (the cumulative use of antidiabetic drugs in the past 3 months has lasted no more than 2 weeks and no antidiabetic drugs has been used in the past month);
- 7% ≤ HbA1c ≤ 9.5% at screening;
- 7 mmol/L≤FPG ≤ 13.3 mmol/L at baseline;
- During the study period and within 60 days after the end of the study, the subjects has no fertility or sperm / egg donation plan and will voluntarily take effective physical contraceptive measures;
- Have given written informed consent to participate in this study, are well motivated, capable, and willing to communicate with the investigator and complete all the requirements according to the protocol.

Exclusion Criteria:

- Those who are known to be allergic to the test drug (including the auxiliary materials of the test drug) or its analogues, or who are allergic to two or more drugs, food and pollen, or who have taken SGLT1 or SGLT2 inhibitors in the past year;
- It was diagnosed as type 1 diabetes, or gestational diabetes, or other special type diabetes;
- There is enough evidence to show that there is proliferative retinopathy of active diabetes;
- History of severe hypoglycemia (such as consciousness disorder and coma caused by hypoglycemia), or history of severe unconsciousness hypoglycemia;
- Organ transplantation history, or other acquired, congenital immune system diseases, or peripheral vascular diseases with clinical significance;
- Have significant hyperglycemia symptoms, such as polyuria, polydipsia, accidental weight loss or dehydration;
- Habitual diarrhea, irritable bowel syndrome, clinically significant abnormal gastric emptying (such as gastric outlet obstruction), severe chronic gastrointestinal diseases (such as active ulcer within 6 months), long-term medication with direct impact on gastrointestinal peristalsis, or gastrointestinal surgery;
- Have obvious blood system diseases (such as aplastic anemia, myelodysplastic syndrome), or any disease causing hemolysis or red blood cell instability (such as malaria), or accompanied by hemoglobin diseases (such as sickle type red blood cell disease) that may affect the determination of HbA1c level;
- Obvious autonomic neuropathy, such as urinary retention, orthostatic hypotension, diabetic diarrhea or gastroparesis.
- History of heart failure (NYHA class Ⅲ and Ⅳ, Appendix 2), or history of acute myocardial infarction or unstable angina within 6 months before screening, or history of coronary angioplasty, coronary stent implantation or coronary bypass surgery within 6 months before screening, or recent cardiac surgery plan;
- Serious trauma, infection or operation that may affect blood glucose control occurred within one month before screening;
- In the first two months of the screening, the drug with weight control effect was used or the operation that can lead to weight instability was performed, or the drug is currently in the weight-loss plan and is not in the maintenance stage;
- Completed or withdrawn an intervention clinical trial within 3 months before screening, or is currently conducting the intervention clinical trial, or participated in other medical research activities, which is not suitable for the study according to the judgment of the researcher;
- Those who frequently drink alcohol (more than 21 units (male) and 14 units / week (female) (1 unit = 360ml beer; or 150ml wine; or 45ml white wine) in the three months before screening, or who can't stop drinking during the test;
- Those who are addicted to smoking (more than 10 cigarettes per day or the same amount of tobacco) within 3 months before screening or who cannot quit smoking (stop nicotine intake) during the trial;
- Those who lost / donated more than 400 ml blood within 3 months before screening (except female physiological blood loss), received blood transfusion or used blood products, or planned to donate blood within 1 month (30 days) after the end of the trial or during the trial;
- To screen the patients with unstable thyroid function (such as thiourea and thyroid hormone drugs) in the first 6 months, with poor control of hypothyroidism or history of hypothyroidism;
- In the first 6 months of screening, there was a history of diabetic acute metabolic complications (diabetic ketoacidosis, hyperosmotic nonketotic coma, diabetic lactate acidosis);
- In the screening period, when no pacemaker was installed, 12 lead ECG showed second degree or third degree atrioventricular block or qtcb interval prolonged more than 500 ms;
- The results of clinical laboratory examination in screening period meet any of the following criteria:
  1. Hemoglobin (Hgb) < lower limit of normal value (LLN);
  2. Aspartate transaminase (AST) or alanine transaminase (ALT) > 2 times of upper limit of normal value (ULN);
  3. Total bilirubin (TBIL) > 1.5 times the upper limit of normal value (except for known Gilbert syndrome which meets the following requirements, that is, part of bilirubin indicates that the combined bilirubin is less than 35% of total bilirubin);
  4. Triglyceride (TG) ≥ 5.7mmol/l;
  5. Estimated glomerular filtration rate < 60 ml / min (estimated by Cockroft Gault formula);
  6. Fasting C peptide < 1.0 ng / ml (333 pmol / L);
  7. Hepatitis B surface antigen, hepatitis C virus antibody, Treponema pallidum antibody or human immunodeficiency virus antibody were screened positive;
- Poor blood pressure control (SBP ≥ 160mmhg and / or DBP ≥ 100mmhg);
- Patients with history of needle syncope, blood syncope or intolerant of venipuncture;
- Those with a history of drug abuse or positive drug abuse screening;
- Patients with obvious mental disorders, epilepsy and other persons without behavioral or cognitive abilities;
- Female subjects in pregnancy, lactation, or with pregnancy intention, or positive pregnancy test (hCG test); and female subjects of childbearing age who can not take effective contraceptive measures (effective contraceptive measures include abstinence, sterilization, intrauterine device, or diaphragm method stipulated by local laws) during the test period;
- The subject may not complete the study for other reasons or the researcher thinks it should not be included.

**Demographic Data**

| Index | 0.5mgBID | 1mgQD | 1mgBID | 2mgQD | 2mgBID | 试验组总计 | 安慰剂 |
| --- | --- | --- | --- | --- | --- | --- | --- |
| Age(year) | | | | | | | |
| N | 8 | 8 | 8 | 8 | 8 | 40 | 10 |
| Mean±SD | 48.1±6.01 | 52.1±6.40 | 53.8±6.50 | 56.9±4.32 | 51.6±5.18 | 52.5±6.16 | 48.7±9.30 |
| Min～Max | 37～54 | 40～62 | 45～64 | 50～63 | 42～59 | 37～64 | 33～59 |
| Median | 49.5 | 52.5 | 55.5 | 57.5 | 51.5 | 53.0 | 51.5 |
| Q1～Q3 | 45.0～52.5 | 49.5～55.5 | 47.5～57.5 | 53.5～60.0 | 49.5～55.0 | 49.0～57.0 | 44.0～56.0 |
| Height(cm) | | | | | | | |
| N | 8 | 8 | 8 | 8 | 8 | 40 | 10 |
| Mean±SD | 164.69±11.052 | 161.25±5.318 | 162.38±7.352 | 161.44±7.331 | 162.50±5.726 | 162.45±7.333 | 167.40±8.478 |
| Min～Max | 151～178 | 155～169 | 153～170.5 | 147～170 | 157～171 | 147～178 | 151～181.5 |
| Median | 162.50 | 160.25 | 164.25 | 161.25 | 159.50 | 160.50 | 169.00 |
| Q1～Q3 | 155.75～176.00 | 156.50～166.25 | 154.50～169.00 | 158.50～167.50 | 158.25～168.25 | 157.00～168.25 | 165.00～170.00 |
| Weight(kg) | | | | | | | |
| N | 8 | 8 | 8 | 8 | 8 | 40 | 10 |
| Mean±SD | 68.30±10.991 | 63.63±9.508 | 66.24±9.546 | 67.43±8.704 | 67.04±11.844 | 66.53±9.780 | 76.89±10.276 |
| Min～Max | 48.5～88.6 | 51.2～82.5 | 53.6～82 | 51.7～79.1 | 56.2～93 | 48.5～93 | 58.9～90 |
| Median | 68.25 | 62.95 | 68.10 | 67.05 | 63.25 | 66.45 | 80.25 |
| Q1～Q3 | 65.20～71.20 | 57.20～67.50 | 57.95～71.10 | 63.05～74.20 | 59.40～70.90 | 60.25～71.15 | 67.90～83.60 |
| BMI (kg/m^2^) | | | | | | | |
| N | 8 | 8 | 8 | 8 | 8 | 40 | 10 |
| Mean±SD | 25.08±2.870 | 24.40±3.292 | 25.04±2.892 | 25.80±2.732 | 25.23±3.315 | 25.11±2.906 | 27.33±2.814 |
| Min～Max | 21.1～28.3 | 19.8～28.8 | 21.4～29.3 | 22.4～30.1 | 21.4～32.1 | 19.8～32.1 | 22.2～31.8 |
| Median | 25.85 | 23.70 | 24.75 | 25.95 | 24.70 | 24.70 | 27.00 |
| Q1～Q3 | 22.35～27.40 | 21.90～27.70 | 22.55～27.50 | 23.40～27.60 | 23.00～26.45 | 22.55～27.50 | 25.50～29.20 |
| Gender | | | | | | | |
| N | 8 | 8 | 8 | 8 | 8 | 40 | 10 |
| 男 n(%) | 5(62.5) | 4(50.0) | 5(62.5) | 5(62.5) | 5(62.5) | 24(60.0) | 8(80.0) |
| 女 n(%) | 3(37.5) | 4(50.0) | 3(37.5) | 3(37.5) | 3(37.5) | 16(40.0) | 2(20.0) |
| Course of type 2 diabetes(month) | | | | | | | |
| N | 8 | 8 | 8 | 8 | 8 | 40 | 10 |
| Mean±SD | 40.20±44.708 | 43.28±47.123 | 38.23±39.026 | 77.16±32.528 | 30.15±14.196 | 45.80±39.093 | 51.45±72.078 |
| Min～Max | 11.5～147.4 | 4.3～121.5 | 5～124.5 | 18.6～122.4 | 12.6～56.3 | 4.3～147.4 | 5.5～243 |
| Median | 26.90 | 18.00 | 30.15 | 77.20 | 27.45 | 32.65 | 19.50 |
| Q1～Q3 | 14.50～39.95 | 5.50～86.70 | 11.10～46.90 | 60.00～100.95 | 20.20～38.50 | 17.00～66.95 | 8.70～67.40 |
| <3 months n(%) | 0(0) | 0(0) | 0(0) | 0(0) | 0(0) | 0(0) | 0(0) |
| ≥3 months n(%) | 8(100.0) | 8(100.0) | 8(100.0) | 8(100.0) | 8(100.0) | 40(100.0) | 10(100.0) |

Demographic characteristics and baseline characteristics were generally balanced among the groups.
